# Differences between Mice and Humans in Regulation and the Molecular Network of Collagen, Type III, Alpha-1 at the Gene Expression Level: Obstacles that Translational Research Must Overcome

**DOI:** 10.3390/ijms160715031

**Published:** 2015-07-03

**Authors:** Lishi Wang, Hongchao Liu, Yan Jiao, Erjian Wang, Stephen H. Clark, Arnold E. Postlethwaite, Weikuan Gu, Hong Chen

**Affiliations:** 1 Department of Orthopedic Surgery and BME-Campbell Clinic, University of Tennessee Health Science Center, Memphis, TN 38163, USA; E-Mails: lwang37@uthsc.edu (L.W.); yjiao2@uthsc.edu (Y.J.); 2 Department of Basic Research, Inner Mongolia Medical College, Inner Mongolia 010110, China; 3 Integrative Research Center, the first Hospital of Qiqihaer City, Qiqihaer 161005, China; E-Mails: hliu48@uthsc.edu (H.L.); ewang5@uthsc.edu (E.W.); 4 Department of Medicine, Mudanjiang Medical College, Mudanjiang 157001, China; 5 Department of Genetics and Developmental Biology, University of Connecticut Health Center, Farmington, CT 06030, USA; E-Mail: sclark@nso2.uchc.edu; 6 Department of Medicine, University of Tennessee Health Science Center, Memphis, TN 38163, USA; E-Mail: apostlet@uthsc.edu; 7 Research Service, Veterans Affairs Medical Center, Memphis, TN 38104, USA

**Keywords:** *COL3A1*, gene network, human, mouse, lung fibrosis

## Abstract

Collagen, type III, alpha-1 (COL3A1) is essential for normal collagen I fibrillogenesis in many organs. There are differences in phenotypes of mutations in the *COL3A1* gene in humans and mutations in mice. In order to investigate whether the regulation and gene network of *COL3A1* is the same in healthy populations of mice and humans, we compared the quantitative trait loci (QTL) that regulate the expression level of *COL3A1* and the gene network of *COL3A1* pathways between humans and mice using whole genome expression profiles. Our results showed that, for the regulation of expression of *Col3a1* in mice, an eQTL on chromosome (Chr) 12 regulates the expression of *Col3a1*. However, expression of genes in the syntenic region on human Chr 7 has no association with the expression level of *COL3A1*. For the gene network comparison, we identified 44 top genes whose expression levels are strongly associated with that of *Col3a1* in mice. We next identified 41 genes strongly associated with the expression level of *COL3A1* in humans. There are a few but significant differences in the *COL3A1* gene network between humans and mice. Several genes showed opposite association with expression of *COL3A1*. These genes are known to play important roles in development and function of the extracellular matrix of the lung. Difference in the molecular pathway of key genes in the *COL3A1* gene network in humans and mice suggest caution should be used in extrapolating results from models of human lung diseases in mice to clinical lung diseases in humans. These differences may influence the efficacy of drugs in humans whose development employed mouse models.

## 1. Introduction

Rodents, particularly mouse and rat have been widely used for biomedical research in models of human diseases since it is known that almost of all of genes in mouse and rat are similar to that of humans. However, not every genetic pathway or molecular mechanism of diseases or drugs discovered to be efficacious in these models can be extrapolated to human diseases. Thus, while much data from animal studies have been successfully applied to humans, some have not. The present study aims to explore the degrees of differences in the causal pathways for lung fibrosis between humans and mice. In spite of the tremendous development in biotechnology and the rapid accumulation of information from genome sequencing and discovery of mutations, progress in the development of therapeutic applications for human fibrotic lung diseases is slow. Most of the new selected targets and drugs developed from animal models have failed in humans. While the failures may be caused by many factors, we believe that genomic differences between humans and animal models may play an important role.

Collagen, type III, alpha-1 (COL3A1) is a major component of the extracellular matrix in a variety of internal organs in human body. COL3A1 is essential for normal collagen I fibrillogenesis in the cardiovascular system and lung. Mutations of the *COL3A1* gene in humans lead to type IV Ehlers-Danlos syndrome, a disease leading to spontaneous rupture of bowel or large arteries in early adult life [1,2]. In contrast to the severe phenotype in humans, mice that are haploinsufficient for *COL3A1* have no identified phenotype. While most have severe disease, about 10% of the homozygous mutant mice survive to adulthood but have a much shorter life span compared with wild-type mice [3,4]. Recently, *Col3a1* was suggested to play a role in the causation of the tight skin phonotype in a mouse model tight skin 2 (Tsk2/+) [5,6], for systemic sclerosis (SSc). A missense mutation in the procollagen III amino terminal propeptide segment (PIIINP) of *Col3a1* was identified in the Tsk2/+ mouse model [6], indicating its complex role in the different tissues and between humans and mice.

*Col3a1* plays an important role in development and fibrosis of the lung [7,8]. Lung malformation in the Ehlers-Danlos syndrome suggests that *COL3A1* plays a critical role in lung development [8] in humans. SSc-related progressive lung fibrosis is another evidence to suggest that *COL3A1* plays an important role in the lung fibrosis [9,10,11]. Recently, a study of *Col3a1* relevant molecular pathways using gene expression profiles in mouse models has shed light on the detailed function of *Col3a1* in lung [10,11]. These studies revealed the importance of the inflammasome and caspase 1 in innate immune signaling, which contributes to SSc fibrosis [10] and osteopontin in the inflammation, mucin production, and gene expression signatures in a murine model of fibrosis [11]. It is important to further study whether the regulation of *COL3A1* and its molecular pathways in lung is the same in the healthy populations between humans and mice, which provides the foundation of disease prevention and treatment. Thus, we need to understand which gene pathways in humans and mice are similar and different in healthy and disease states.

This study aims to explore the potential pathways of *Col3a1* in lung using data of whole genome expression in mouse models and humans. We will focus on the molecular pathways of *Col3a1* in normal lung tissues to investigate what roles *Col3a1* plays in the normal function of lung, which is a targeted organ in fibrotic disease. Understanding the gene network of *Col3a1* in normal genomic background will provide information on how *Col3a1*is regulated and how *Col3a1* regulates other genes. In this study, we will focus on the identification of the genetic loci that regulate the expression of *Col3a1,* construction of pathways of *Col3a1* under the normal genomic background, and comparing the similarity and differences of molecular pathways between humans and mice.

## 2. Results

### 2.1. Transcriptome Mapping of Expression Quantitative Trait Loci (eQTL) that Regulates Expression Level of Collagen, Type III, alpha-1 (COL3A1)

**Figure 1:**
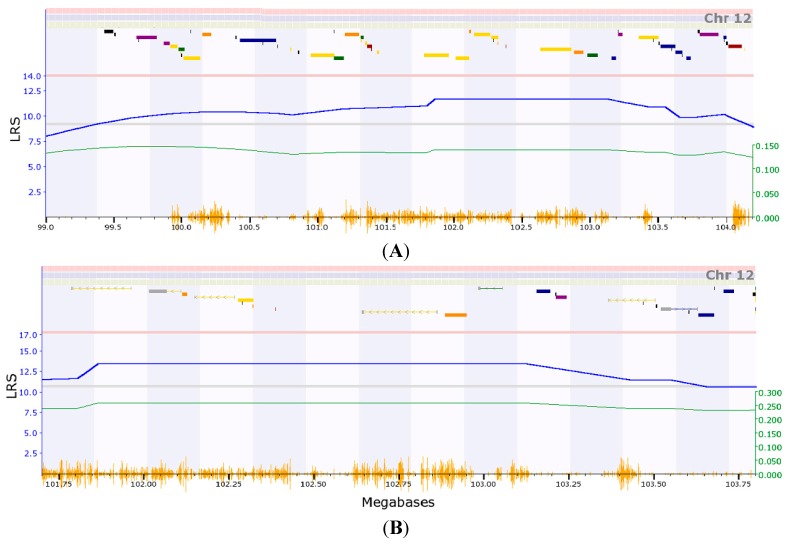
The expression quantitative trait loci (eQTL) that regulates the expression level of Collagen, type III, alpha-1 (*Col3a1*). (**A**) eQTL on chromosome (Chr) 12 located between 99 MB (million bases) and 104 detected with probe 1724883; (**B**) eQTL located on Chr 12 region between 101.7 and 103.8 MB.

We first mapped the expression quantitative trait loci (eQTL) that regulates the expression level of *Col3a1* using data of the mouse model, the HZI Lung M430v2 (Apr08) RMA Database [12]. The dataset has three probes of *Col3a1*. They are from the intron (GeneNetwork ID: 1442977_at), last two exons proximal and mid 3′ UTR (ID: 1427883_a_at) and distal 3′ UTR (ID: 1427884_at). Probe 1427883 was first used for transcriptome mapping. The transcriptome mapping located two eQTL at the suggestive level (Figure S1A). One is located between 99 MB (million bases) and 104 MB on chromosome (Chr) 12 (Figure 1A), which contains 57 genetic elements as candidates (Table S1). The other is located on Chr 11, between 19.3 MB and 28 MB which also contain 57 genetic elements.

Using probe 1427884_at, we mapped tow loci (Figure S1B), one is located on Chr 12, the same genomic region with that mapped by probe 1427883_a_at (Figure 1B). The other is located on Chr 9. The EQTL on Chr 12 was mapped into a small genomic region between 101.7 and 103.8 MB, which contains 24 genetic elements and 16 known genes (Table 1).

Probe 1442977 from intron has a relatively low expression level. Transcriptome mapping showed that a merely suggestive locus on Chr 10 regulates the expression of this probe (Figure S1C). Because of its nature of intron location, low expression level, and uncertainty of eQTL, we used the data from other two probes for the further analysis.

**Table 1:** Candidate genes for eQTL of regulation of *Col3a1* expression level in mice.

Index	SNPS Name	Chr	MB	−log(*p*)	Gene Name	Function Consequence
1	exm-rs2585897	13	21.398979	4.82131	*XPO4*	intron variant
2	exm1564625	21	30.925928	4.69919	*–*	*–*
3	exm2272985	21	42.047446	4.29611	*–*	*–*
4	exm2270333	6	58.344955	4.04571	*CRIM1*	intron variant
5	exm609871	7	23.811795	3.93442	*–*	*–*
6	exm-rs7326068	13	21.209512	3.69486	*IFT88*	intron variant
7	exm-rs10506525	12	65.783378	3.52754	*MSRB3*	intron variant
8	exm1082069	13	114.175038	3.51385	*–*	*–*
9	exm1157141	15	44.093927	3.45730	*ZFPM2*	intron variant
10	exm2266405	7	46.919134	3.44515	*CCDC144A*	intron variant
11	exm2266494	7	153.144983	3.27992	*–*	*–*
12	exm-rs4380451	3	32.329497	3.27737	*CMTM8*	intron variant
13	exm2269464	3	69.422051	3.25987	*PHEX*	intron variant
14	exm2271745	12	65.810174	3.25088	*–*	*–*
15	exm506442	5	176.637576	3.21638	*TLDC1*	intron variant
16	exm869284	11	0.193146	3.17341	*–*	*–*
17	exm2267394	12	117.082846	3.14527	*GTPBP1*	upstream variant 2 KB
18	exm2273373	3	52.874288	3.13365	*GPCPD1*	missense
19	exm882858	11	5.461834	3.11867	*–*	*–*
20	exm-rs2286720	3	42.448471	3.10924	*LYZL4*	synonymous codon
21	exm-rs2763196	9	117.150951	3.10885	*AKNA*	intron variant, upstream variant 2 KB
22	exm-rs11073328	15	38.764843	3.10746	*FAM98B*	intron variant
23	exm397086	4	42.895308	3.09675	*MEGF10*	intron variant
24	exm1156850	15	43.939642	3.03829	*RP11-23B15.1*	intron variant

### 2.2. Initial Analysis of Candidate Genes for Regulation of Expression of Col3a1 on Mouse Chromosome (Chr) 12

Because two probes of *Col3a1* located the eQTL onto the same location on Chr 12, we further examined the genes within the eQTL region to identify the potential candidate genes. We first examined the association of expression levels of these genes in the eQTL with that of *Col3a1*. According to the map based on probe #1427883, there are 57 genetic elements within the eQTL (Table S1), among them, 34 are known genes. (*Galc*, *Gpr65*, *Kcnk10*, *Spata7*, *Ptpn21*, *Zc3h14*, *Eml5*, *Ttc8*, *Foxn3*, *Ttc7b*, *Tdp1*, *Kcnk13*, *Psmc1*, *Calm1*, *Rps6ka5*, *Gpr68*, *Ccdc88c*, *Smek1*, *Trip11*, *Catsperb*, *Tc2n*, *Fbln5*, *Atxn3*, *Cpsf2*, *Slc24a4*, *Gm46*, *Rin3*, *Lgmn*, *Golga5*, *Chga*, *Itpk1*, *Moap1*, *Btbd7*, *Cox8c*). According to the map based on probe #1427884, the eQTL region contains only 24 genetic elements (Table S2), from which we found 16 genes (*Rps6ka5*, *Gpr68*, *Ccdc88c*, *Smek1*, *Trip11*, *Catsperb*, *Tc2n*, *Fbln5*, *Atxn3*, *Cpsf2*, *Slc24a4*, *Gm46*, *Rin3*, *Lgmn*, *Golga5*, *Chga*). All of these 16 genes are among the list for eQTL based on the probe #1427883.

**Figure 2:**
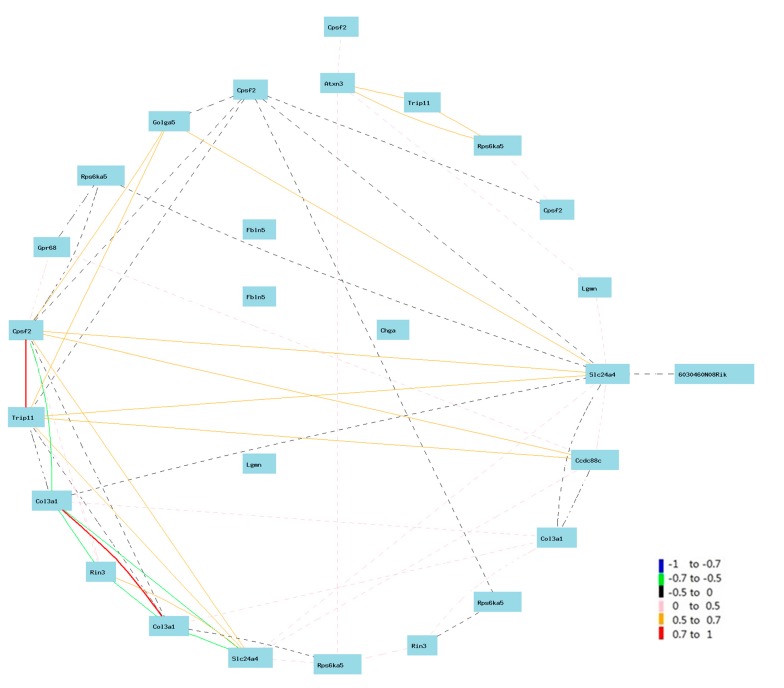
Gene network between *Col3a1* and candidate genes on Chr 12. The 27 nodes in the graph below show the selected traits. All nodes are displayed. The 55 edges between the nodes, filtered from the 351 total edges and drawn as curves, show Pearson correlation coefficients greater than 0.35 or less than −0.35. The graph’s canvas is 40.0 by 40.0 cm, and the node labels are drawn with a 18.0 point font, and the edge labels are drawn with a 16.0 point font.

To make sure we did not miss the real candidates, we used the gene list of eQTL of #1427883 for further analysis. For the convenience of identifying genes linked to *Col3a1*, we used the circular layout model for the building of gene network in this case. The network showed that the expression levels of both probes of *Col3a1* are negatively correlated to *Slc24a4* and *Rin3* (Figure 2). Because both genes are among the candidate list from two eQTLs of two probes, their likelihood of candidacy are strong. In addition, one probe (#1427883) of *Col3a1* showed a weak positive correlation with *Zc3h14.* One probe (#1427884) is negatively correlated to *Cpsf2.*

We next searched the publications that match these candidate genes with *Col3a1* using PGMapper [13]. Our search indicated that none of these genes has been linked to *Col3a1* in the literature.

### 2.3. Narrowing down the Candidate Genes for Regulation of Expression of Col3a1 on Mouse Chr 12

From our initial analysis, we found that expression of four genes are associated with that of *Col3a1*: *Slc24a4* (103.367628 MB), *Rin3* (103.521850 MB), *Cpsf2* (103.214183 MB), and *Zc3h14* (at 99.985177 MB). From data from the gene network analysis, we surprisingly found that membrane targeting (tandem) C2 domain containing 1 (*Mtac2d1*) is positively correlated to the expression of *Col3a1*. It is located on Chr 12, starting on 102.883731 MB, the peak region of the eQTL. These data suggests that most likely the causal gene for the regulation of expression of *Col3a1* is located in the genomic region nearby 103 MB.

We next conducted association analysis of these five genes with the top 44 genes that correlated to the expression level of *Col3a1*. We assumed that if these genes are the regulator of *Col3a1*, they should also influence the expression of some of these top 44 genes that are downstream of *Col3a1*. Gene network analysis indicated that *Mtac2d1, Cpsf2*, and *Slc24a4* interact with each other while *Mtac2d1* influences the expression of more than 20 of these *Col3a1* relevant genes. *Rin3* and *Zc3h14* interact with each other and have influence on only three of the 44 genes. Thus, the candidate gene could be *Mtac2d1*, *Cpsf2*, or *Slc24a4*, within the genomic region between 102.883731 MB and 103.367628 MB, based on the genome information in GeneNetwork.

We then examined the polymorphism of single nucleotide polymorphisms (SNPS) on these genes in each C57BL/6J X DBA/2J (BXD) strain and their expression level of *Col3a1*. We first searched the SNPS between C57BL/6J and DBA/2J mouse strains from MGI (Mouse Genome Informatics) database, (http://www.informatics.jax.org). For each gene, we searched the gene as well as 2 kb up and down stream. *Mtac2d1* has no SNPS. *Cpsf2* has 30 SNPS including five upstream and downstream. *Slc24a4* contains 123 SNPS. According to the Ensembl genome database, these two genes are next to each other on Chr 12. Therefore, these two genes could be acting together or individually in regulation of *Col3a1* expression.

### 2.4. Gene Network of Col3a1-Related Genes in Lung in Mouse Model

**Figure 3:**
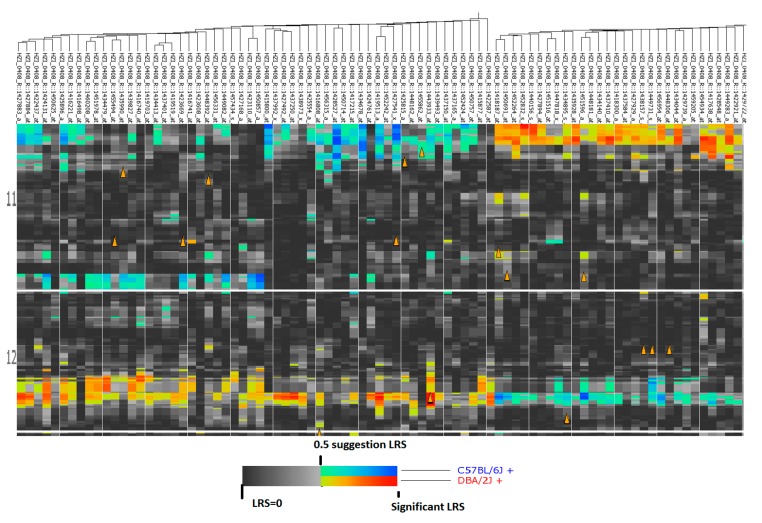
Heatmap showing regulation of genes on Chr 11 and 12 that are closely correlated to *Col3a1*. The influence of these two Chrs. On Chr 11 expression of first part of genes are affected by C57BL/6J (B6) and the last part of the genes are affected by DBA/2J (D2), while on Chr 12, the effect is opposite to that on Chr 11. Orange triangles are genetic elements (probes) that are correlated to the expression level of *Col3a1.*

Using data on the expression level of Probe 1427883 and whole genome expression profiles in the recombinant inbred (RI) strains, we identified the top 100 probes of genes with expression levels most correlated to that of *Col3a1* from the mouse data of HZI Lung M430v2 (Apr08) RMA Database (Table S3 and Figure S2). The expression of mouse *Col3a1* is most positively correlated to procollagen, type V, alpha 2 (*Col5a2*), procollagen, type 1, alpha 2 (*Col1a2*), fibrillin 1 (*Fbn1*), procollagen, type I, alpha 1 (*Col1a1*), a disintegrin-like and metalloprotease with thrombospondin type 1 motif, 12 (*Adamts12*), insulin-like growth factor 1 (*Igf1*), follistatin-like 1(*Fstl1*), lysyl oxidase-like 1 (*Loxl1*), and procollagen, type V, alpha 1(*Col5a1*). It negatively correlated to receptor (calcitonin) activity modifying protein 2 (*Ramp2*), nuclear factor of kappa light chain gene enhancer in B-cells inhibitor, alpha (*Nfkbia*), sphingosine kinase 1 (*Sphk1*), pre-B lymphocyte gene 3 (*Vpreb3*), and tensin like C1 domain-containing phosphatase (*Tenc1*). Heatmap analysis indicated that there is a large cluster effect that significantly influences the expression of these genes (Figure S3). For example, there is a significant difference on the effect of gene expression levels between Chr 11 and Chr 12. Figure 3 shows the influence of these two chromosomes. On Chr 11 expression of the first part of genes are affected by C57BL/6J (B6) and the last part of the genes are affected by DBA/2J (D2), while on Chr 12, the effect is opposite to that on Chr 11.

With probe 1427884, we obtained the second 100 probes of genes (Table S4). Similar to that of probe 1427883, the expression of mouse *Col3a1* is positively correlated to *Col5a2*, *Col1a2*, *Fbn1*, *Col1a1*, *Adamts12*, *Igf1*, *Fstl1*, *Loxl1*, and *Col5a1*. It is negatively correlated to *Ramp2*, *Nfkbia*, *Sphk1*, *Vpreb3*, and *Tenc1*. Heatmap analysis also indicated that there is large cluster effect that significantly influences the expression of these genes (Figure S4).

**Figure 4:**
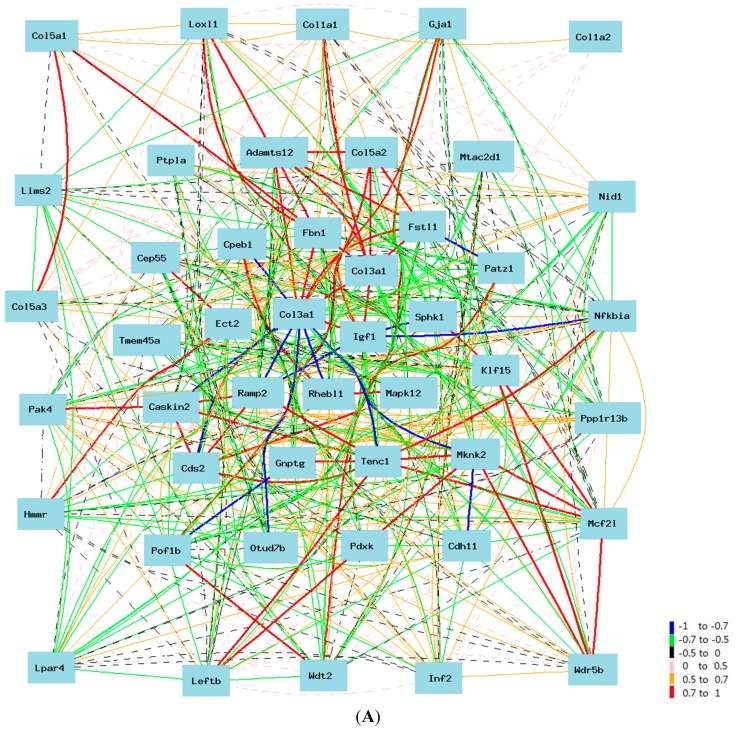

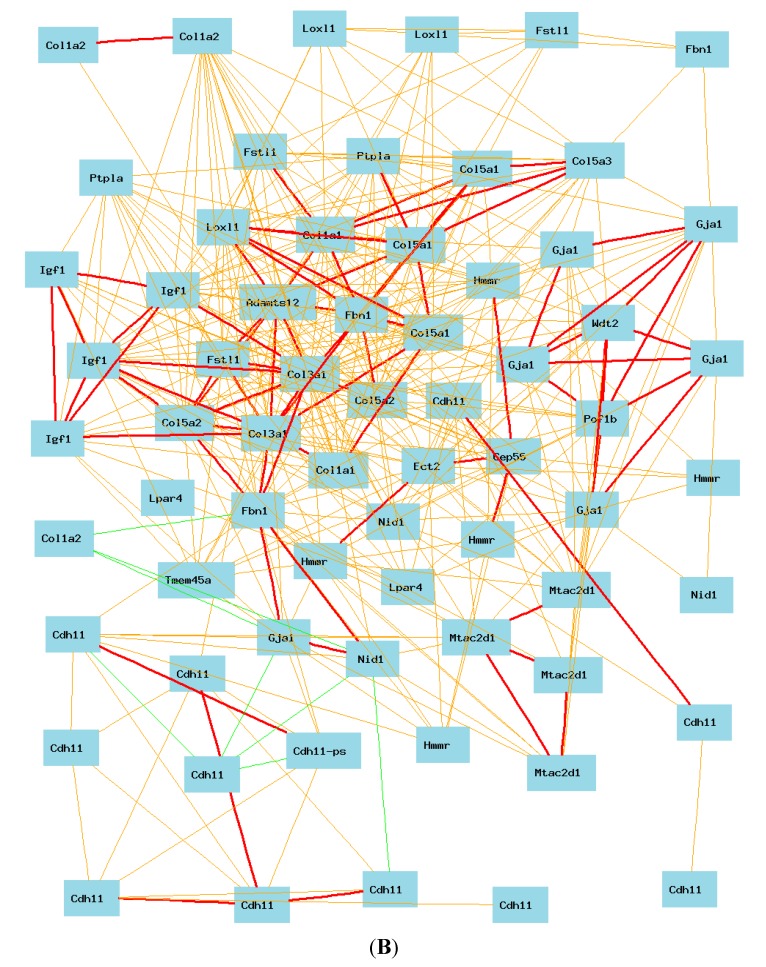
(**A**) Gene network of 44 common genes from two probes. The 46 nodes in the graph below show the selected probes. Only nodes with edges are displayed. The 866 edges between the nodes, filtered from the 1035 total edges and drawn as curves, show Pearson correlation coefficients greater than 0.35 or less than −0.35. The graph’s canvas is 40.0 by 40.0 cm, and the node labels are drawn with a 14.0 point font, and the edge labels are drawn with a 14.0 point font; (**B**) Two probes of *Col3a1* show their positive correlation with 22 genes in mice. The 74 nodes in the graph below show the selected genes. Only nodes with edges are displayed. The 431 edges between the nodes, filtered from the 2701 total edges and drawn as lines, show Pearson correlation coefficients greater than 0.5 or less than −0.5. The graph’s canvas is 40.0 by 40.0 cm, and the node labels are drawn with a 14.0 point font, and the edge labels are drawn with a 14.0 point font.

By comparing the gene list between probe 1427883 and 1427884, we identified 44 genes that are in both lists (*Adamts12*, *Caskin2*, *Cdh11*, *Cds2*, Cep55, *Col1a1*, *Col1a2*, *Col5a1*, *Col5a2*, *Col5a3*, *Cpeb1*, *Ect2*, *Fbn1*, *Fstl1*, *Gja1*, *Gnptg*, *Hmmr*, *Igf1*, *Inf2*, *Klf15*, *Leftb*, *Lims2*, *Loxl1*, *Lpar4*, *Mapk12*, *Mcf2l*, Mknk2, *Mtac2d1*, *Nfkbia*, *Nid1*, *Otud7b*, *Pak4*, *Patz1*, *Pdxk*, *Pof1b*, *Ppp1r13b*, *Ptpla*, *Ramp2*, *Rhebl1*, *Sphk1*, *Tenc1*, *Tmem45a*, *Wdr5b*, *Wdt2*). We used the 44 probes of these genes and two probes of *Col3a1* in the construction of the network of key genes in the *Col3a1* pathway (Figure 4A). During the construction of the network, we selected one probe from each of these genes. In case of multiple probes, one probe with highest expression level and commonly associated with two probes of *Col3a1* is chosen.

**Figure 5:**
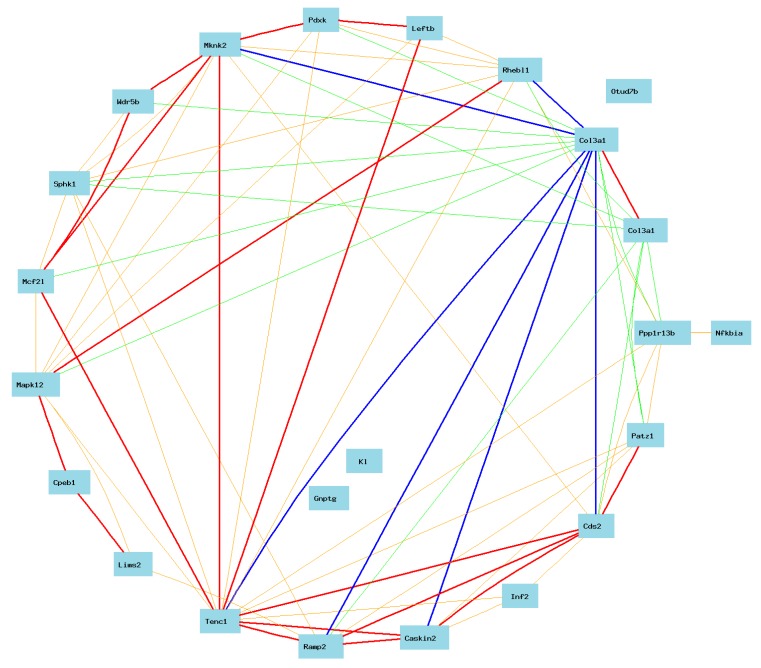
Two probes of *Col3a1* show their negative correlation with 21 genes in mice. The 23 nodes in the graph below show the selected traits. All nodes are displayed. The 69 edges between the nodes, filtered from the 253 total edges and drawn as curves, show Pearson correlation coefficients greater than 0.6 or less than −0.6. The graph’s canvas is 40.0 by 40.0 cm, and the node labels are drawn with a 16.0 point font, and the edge labels are drawn with a 16.0 point font.

According to the final network of these genes, the following genes are up-regulated by *Col3a1*: *Adamts12*, *Cdh11*, Cep55, *Col1a1*, *Col1a2*, *Col5a1*, *Col5a2*, *Col5a3*, *Ect2*, *Fbn1*, *Fstl1*, *Gja1*, *Hmmr*, *Igf1*, *Loxl1*, *Lpar4*, *Mtac2d1*, *Nid1*, *Pof1b*, *Ptpla*, *Tmem45a*, *Wdt2* (Figure 4B). The following genes are down-regulated by *Col3a1*: *Caskin2*, *Cds2*, *Cpeb1*, *Gnptg*, *Inf2*, *Klf15*, *Leftb*, *Lims2*, *Mapk12*, *Mcf2l*, Mknk2, *Nfkbia*, *Otud7b*, *Patz1*, *Pdxk*, *Ppp1r13b*, *Ramp2*, *Rhebl1*, *Sphk1*, *Tenc1*, *Wdr5b* (Figure 5). One probe of *Col3a1* is negatively while the other is positively correlated to that of *Pak4*.

### 2.5. Gene Network of COL3A1 in Lung in Humans

We then analyzed the gene network of *COL3A1* using data in Super Series GSE23546 Whole-Genome GXD Non-Tumorous Human Lung Tissues Affy HuRSTA array (RMA Database [14]). From the data, we found four probes for *COL3A1* (Probe ID: 100149328_TGI_at, 100303661_TGI_at, 100310834_TGI_at, 100312190_TGI_at). They are all located on Chr 2 in the same region (Chr 2: 189.839099). Probe 100312190_TGI_at has the lowest mean expression level. While the expression levels of other three probes are all higher than 11 (relative expression level used in GeneNetwork), the expression level of probe 100312190_TGI_at is 7.6. Correlation analysis indicated that probe 100312190 is not strongly correlated to the expression levels of other three probes (Figure S5). Accordingly, probe 00312190 was not used in the further analysis.

**Figure 6:**
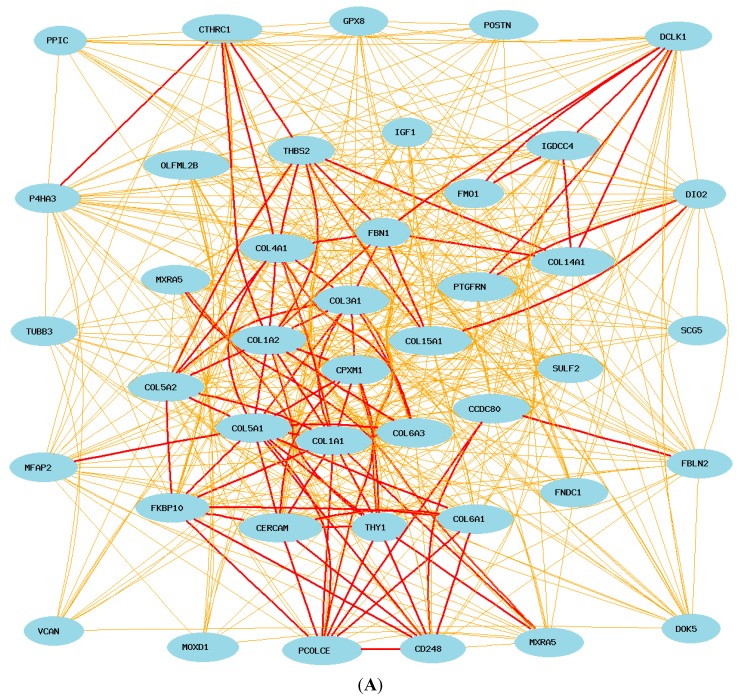

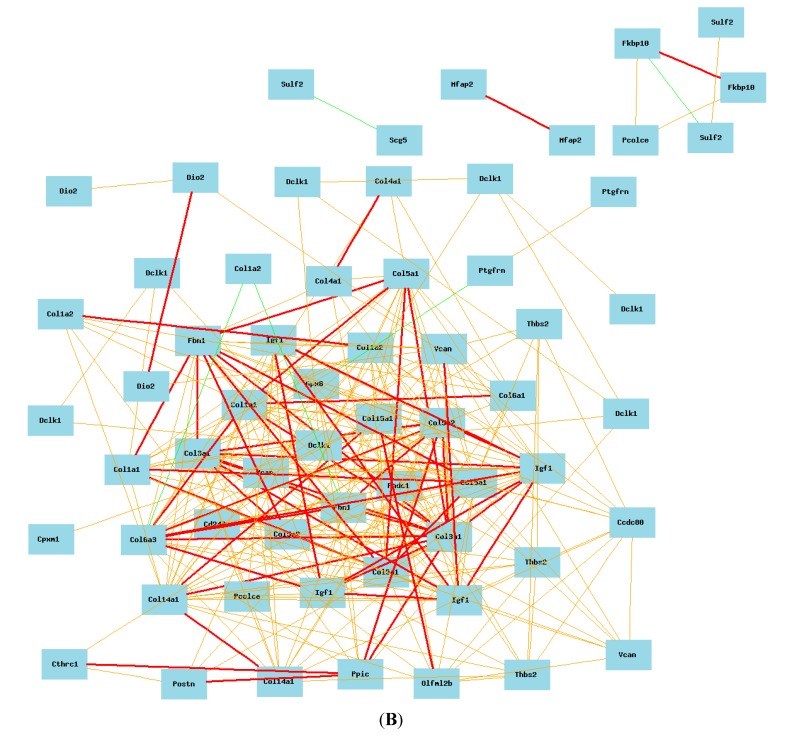
Gene networks of common genes in mice and humans. Each subfigure shows Pearson correlation coefficients greater than 0.5 or less than −0.5. The graph’s canvas is 40.0 by 40.0 cm, and the node labels are drawn with a 14.0 point font, and the edge labels are drawn with a 14.0 point font. (**A**) Gene network of 41 common genes and *COL3A1* in human lung with probe 100149328. The 42 nodes in the graph below show the selected traits. All nodes are displayed. The 566 edges between the nodes, filtered from the 861 total edges and drawn as curves; (**B**) Correlations between the 41 common top human genes with *Col3a1* in mouse lung. The 89 nodes in the graph below show the selected traits. Only nodes with edges are displayed. The 289 edges between the nodes were filtered from the 3916 total edges and drawn as lines.

By comparing the top 100 probes lists (presenting approximately 70 genes) for the association to the three probes (Tables S5–S7 and Figure S6A–C), we identified 41 genes that correlate to the expression of all three probes of *COL3A1*, *CCDC80*, *CD248*, *CERCAM*, *COL14A1*, *COL15A1*, *COL1A1*, *COL1A2*, *COL4A1*, *COL5A1*, *COL5A2*, *COL6A1*, *COL6A3*, *CPXM1*, *CTHRC1*, *DCLK1*, *DIO2*, *DOK5*, *FBLN2*, *FBN1*, *FKBP10*, *FMO1*, FNDC1, *GPX8*, IGDCC4, *IGF1*, *MFAP2*, *MOXD1*, *MXRA5*, *MXRA5*, *OLFML2B*, *P4HA3*, *PCOLCE*, *POSTN*, PPIC, *PTGFRN*, *SCG5*, *SULF2*, *THBS2*, *THY1*, *TUBB3*, *VCAN*). Unlike mouse lung, in human lung these genes are all positively correlated to the expression of *COL3A1* (Figure 6A).

### 2.6. Potential Difference in Regulation of Expression of COL3A1 between Human and Mice

We identified the probes of 16 candidate genes from the human gene expression data, based on the 16 genes from mouse eQTL data. We analyzed the correlation between their expression levels and that of all probes of *COL3A1* in humans. Data showed that none of these genes had expression levels associated to that of *COL3A1* (Figure S7A).

We then examined the association between these same 16 genes with *COL3A1* in the data of RNAseq, which is from a different human population [14,15]. The data again showed that the expression of *COL3A1* does not have correlation with the expression level of any of these 16 genes (Figure S7B).

These data suggest that potentially there is a difference in regulation of *Col3a1* in mice and *COL3A1* in humans.

### 2.7. Gene Network between Mice and Humans

Comparing data from mice and humans, we found that several key genes overlap between mice and humans including *COL1A1*, *COL1A2*, *COL5A1*, *COL5A2*, *FBN1*, and *IGF1*. However, there are apparently some significant differences. In humans, *COL3A1* correlates to *COL14A1*, *COL15A1*, *COL4A1*, *COL6A1*, and *COL6A3*, but these genes are not among the top list in mice. Therefore, we further compared the gene network of mice and humans.

We searched the probes of these 41 human genes in the mouse microarray data and examined their association with *Col3a1*. We obtained a total of 87 probes for these genes. Data showed that although some of these genes are not in the list of the top 100 most closely correlated probes of genes, most of them are positively correlated in mice to the *Col3a1* (*R* ≥ 0.5) (Figure 6B). Exceptions are *Sulf2*, *Mfap2*, *Scg5*, and *Fkbp10*. These four genes are not associated with *Col3a1* in mice. The other difference is that *Gpx8* is negatively correlated to *Ptgfrn*, One probe of *Sulf2* is negatively correlated to *Scg5* while the other is negatively correlated to that of *Fkbp10*. Interestingly, one of the two probes of *Col1a2* is negatively correlated to one of the *Col6a3* and *Fbn1*.

**Figure 7:**
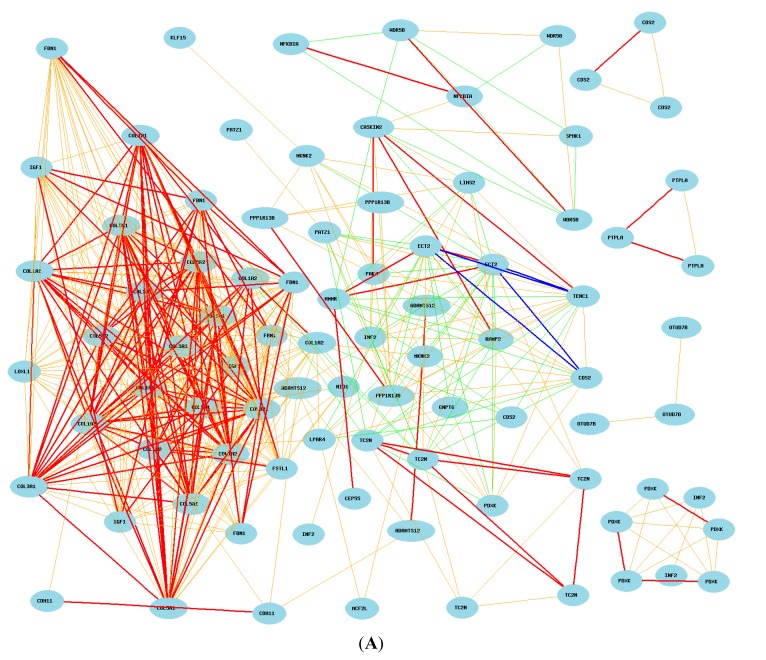

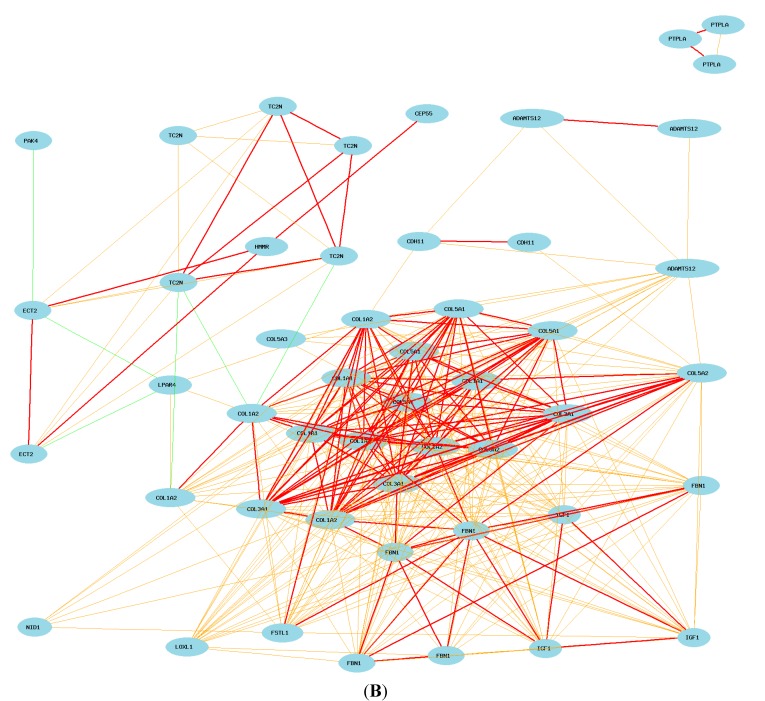

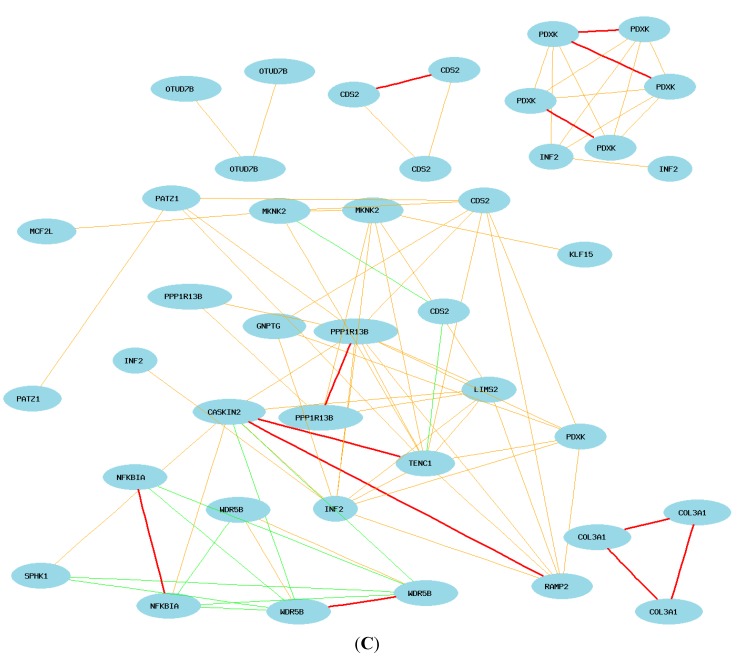
Gene network of top collated genes in mice and humans. Each figure shows Pearson correlation coefficients greater than 0.5 or less than −0.5. The graph’s canvas is 40.0 by 40.0 cm, and the node labels are drawn with a 14.0 point font, and the edge labels are drawn with a 14.0 point font. (**A**) Correlation between *COL3A1* and 104 probes of top 44 genes in mice in human lung. The 107 nodes in the graph below show the selected traits. Only nodes with edges are displayed. The 517 edges between the nodes were filtered from the 5671 total edges and drawn as lines; (**B**) Gene network between *COL3A1* and 22 unregulated genes selected from mice in human lung tissues. The 59 nodes in the graph below show the selected traits. Only nodes with edges are displayed. The 386 edges between the nodes were filtered from the 1711 total edges and drawn as lines; (**C**) The gene network between *COL3A1* and 21 down-regulated genes selected from mice in human lung tissues. The 51 nodes in the graph below show the selected traits. Only nodes with edges are displayed. The 83 edges between the nodes were filtered from the 1275 total edges and drawn as lines.

We then searched the probes of 44 mouse genes from human microarray data and compared them to the expression levels of probes of *COL3A1* in humans. We obtained 104 probes for these 44genes. We found that the majority of genes showed association with *COL3A1* in human lung (Figure 7A). However, *PTPLA* showed no connections to *COL3A1*.

We then further examined whether the 22 genes up-regulated or down-regulated by *Col3a1* in mice are the same in humans [16,17,18]. A total of 56 probes were identified for the 22 up-regulated genes. While most of these genes are positively correlated with each other, a few negative correlations were observed (Figure 7B). *COL1A2* showed a negative correlation to *TC2N*. *LPAR4* showed a negative correlation with *ECT2*, which is positively correlated to *HMMR* and *CEP55*. Thus, the *COL3A1* network down-regulates the expression of *TC2N* through *COL1A2*. The *COL3A1* network down-regulates *ECT2*, *HMMR* and *CEP55* through *LPAR4*.

We next analyzed the 21 genes down-regulated by mouse *Col3a1* in humans. A total of 48 probes were identified from gene expression profiles of human lung. Surprisingly, there is no strong connection (Pearson correlation coefficients greater than 0.5 or less than −0.5) to these genes from any of three probes of *COL3A1* (Figure 7C). When the criteria is lowered to the Pearson correlation coefficients greater than 0.35 or less than −0.35, *COL3A1* showed a weak negative correlation with *OTUD7B* and *CASKIN2*. Combining the information with the genes that are up-regulated, it seems that the *COL3A1* may down-regulate these genes through the up-regulated genes. *COL1A2* seems to play a major role in the negative regulation of these genes. The other pathway may be through *LPAR4*, which is negatively correlated to INF2 and CDS2.

### 2.8. Potential Differences in Gene Network of COL3A1 between Humans and Mice

**Figure 8:**
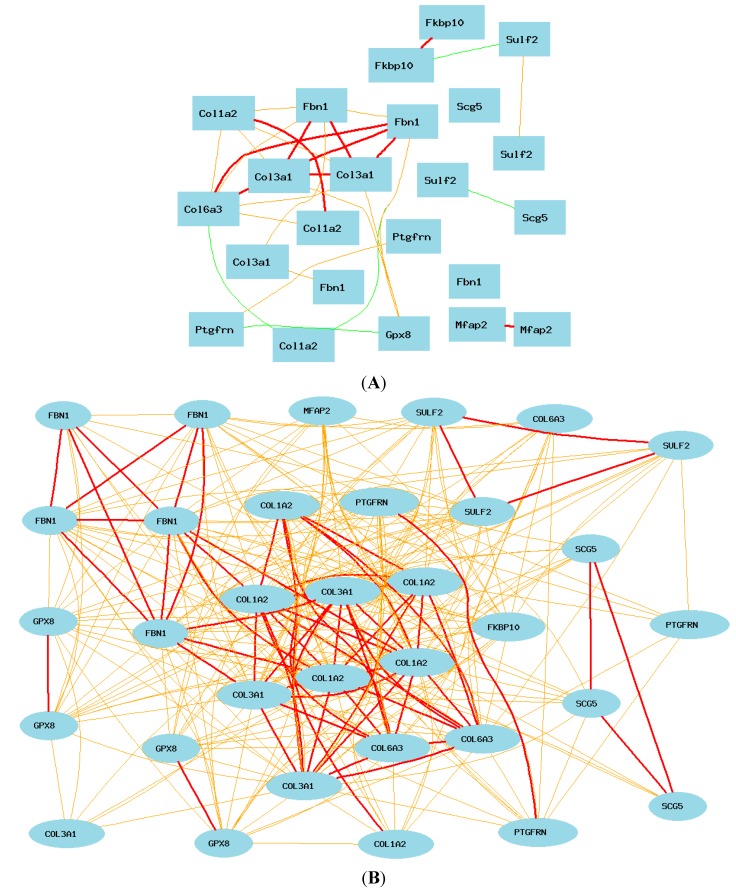

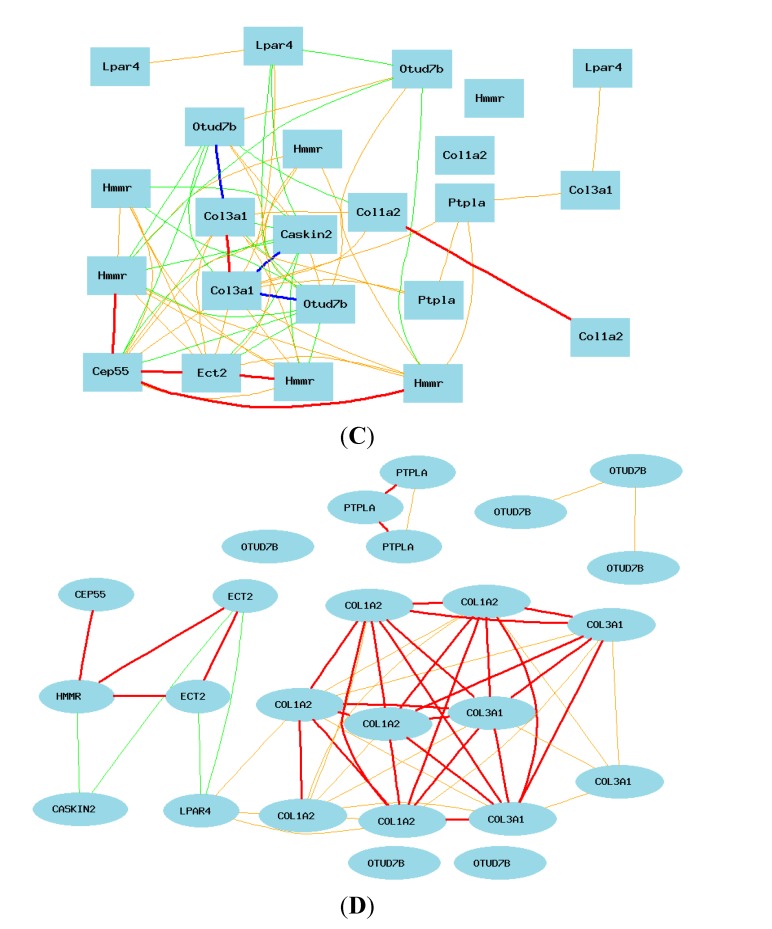
Summary of the gene networks of humans and mice. Each picture shows Pearson correlation coefficients greater than 0.5 or less than −0.5. The graph’s canvas is 40.0 by 40.0 cm, and the node labels are drawn with a 16.0 point font, and the edge labels are drawn with a 16.0 point font. Solid lines represent strong correlation (absolute value: thick line = 0.7–1; thin line = 0.5–0.7), while dashed lines present weak correlation (absolute value: 0–0.5). (**A**) Summary of the network of human-positive genes in mice. The 23 nodes in the graph below show the selected genes. The 30 edges between the nodes were filtered from the 253 total edges and drawn as curves; (**B**) Summary of the network of positive genes in human microarray data. The 33 nodes in the graph below show the selected genes. All nodes are displayed. The 288 edges between the nodes were filtered from the 528 total edges and drawn as curves. The graph’s canvas is 40.0 by 40.0 cm; (**C**) Summary of network of mouse genes: the 23 nodes in the graph below show the selected genes. The 66 edges between the nodes were filtered from the 253 total edges and drawn as curves; (**D**) Summary of gene network genes from mice in human microarray data. The 25 nodes in the graph below show the selected genes. The 55 edges between the nodes were filtered from the 300 total edges and drawn as curves.

The above analyses allowed us to identify potential differences in the gene networks of mice and humans. In order to define the difference in the gene network of *COL3A1* between mice and humans, we further constructed a network using core genes that showed differences between humans and mice. We first used the eight *COL3A1* up-regulated genes in humans, *SULF2*, *FKBP10*, *COL6A3*, *GPX8*, *PTGFRN*, *COL1A2*, *SCG5*, *FGN1*, and *MFAP2* to construct the network in mice. As shown in Figure 8A, in mice, only collagens and *Fbn1* are strongly positively correlated together. The expression of *Sulf2*, *Mfap2*, *Scg5*, and *Fkpb10* are not associated to the expression of *Col3a1*. *Gpx8* is weakly positively associated with *Col3a1*. In humans, the expression of all of these genes is positively associated (Figure 8B). We next examined the difference of 11 key genes originally from analysis of mouse expression profiles (*Ect2*, *Hmmr*, *Cep55*, *Lpar4*, *Col1a2*, *Col3a1*, *Lpar4*, *Cep55*, *Ptpla*, *Otud7b*, or *Caskin2*). We found a total of 23 records from the mouse data (Figure 8C). Most of these genes are negatively associated to the expression level of *Col3a1*. Particularly, the expression of *Col3a1* is strongly negatively correlated to that of *Caskin2* and *Otud7b. Col3a1* is positively associated to *Ptp1a* and *Cep55*. In humans, while the core part of the gene network is similar to that of the mice, probes of *OTUD7B*, *PTPLA*, *CEP55* and *ECT2* are not or not directly associated with other genes (Figure 8D). By GeneSet analysis, we found that, among the differentially regulated genes, four genes belong to the extracellular region, *COL1A2*, *MFAP2*, *CEP55*, and *SULF2*. *COL1A2*, *SULF2*, *PTGFRN*, and *PTPLA* are also located on the endoplasmic reticulum. *LPAR4* and *ECT2* are lipid binding proteins. In summary, the pathways of *Sulf2*, *Mfap2*, *Scg5*, *Fkpb10*, *Gpx8*, *Oud7b*, *Ptpla*, *Cep55* and *Ect2* in relation to *Col3a1* are potentially different between mice and humans.

## 3. Discussion

Our analysis revealed key differences in the regulation of *COL3A1* between humans and mice. While many genetic elements may influence the expression of *Col3a1* in mice, the eQTL on Chr 12 is one of the genetic factors that regulates the expression of *Col3a1*. We found that the expression levels of at least two genes within the eQTL region are negatively associated with that of *Col3a1* in mice. The homologous region on human chromosome 7 does not seem to have any gene expression correlated to the expression of *COL3A1*. Although the eQTL is only at the suggestive level, the same genomic region was mapped by two probes, suggesting that it is a reliable locus; such a difference deserves further investigation. The opposite effect on several genes between mice and humans raises caution on the using mouse model for the study of *COL3A1* relevant lung diseases in humans. Among nine genes that are positively correlated to the expression of *COL3A1* in humans, some are not connected and others are not positively correlated to *Col3a1* in mice. Surprisingly, none of these genes have been studied together with *Col3a1*, based on searching the PubMed database. They apparently have not yet been brought to the attention of investigators studying lung diseases such as lung fibrosis. Some of these genes in fact have been linked to lung fibrosis. For example, *SULF2* is over expressed in idiopathic pulmonary fibrosis [19]. *CEP55* has been reported to contain polymorphisms significantly associated with bleomycin-induced fibrotic lung disease [20]. There is a report on the developmental regulation and coordinate reexpression of *FKBP65* (*Fkpb10*) with extracellular matrix proteins after lung injury [21]. In genome-wide association study and large-scale follow up, *MFAP2* has been identified as one of the candidate genes influencing lung function [22].

We believe that it is important to investigate whether the expression of these genes is altered by *COL3A1*, or other closely related collagen family members, in relevant diseases. Perhaps one of the big misleading concepts resulting from elucidation of the first human and mouse genome sequences is that genes in both are almost the same [23,24,25,26]. While it is true that the majority of sequences of genes between mice and humans are similar, there are many reasons for the failure in clinic trails in humans of the majority of drugs derived from studies using animal models. One of the reasons may be the lack of awareness of the differences in molecular pathways between humans and mice when employing the animal model. Study of these differentially regulated genes is critical in the selection of molecular targets for the diseases influenced by *COL3A1* and its closely related collagen family members.

The difference in these genes may play essential roles in affecting differential development of lung cells between mice and humans. By their function, these differentially regulated genes are important for lung cell development. They are essential for the cellular components and organization. Particularly, *MFAP2* known as the microfibrillar-associated protein is important in structure and function of the extracellular matrix [22]. *Sulf2* mRNA is overexpressed in lung samples from human patients with idiopathic pulmonary fibrosis (IPF), and Sulf2 protein was specifically localized to the hyperplastic type II alveolar epithelial cells (AECs) [27]. Polymorphism of Cep55 has been significantly associated with bleomycin-induced fibrotic lung disease in mice [20]. The differential regulation of these genes by *COL3A1* and *COL1A2* between humans and mice imposes a critical question on how these differences impact the molecular consequence and the disease phenotype.

Our study takes advantage of available data of whole genome gene expression profiles from mouse lung and human lung. These two sets of data have relatively large numbers of samples. The data reliability have been previously tested [12,14]. In addition, the fact that we detected the close correlation between *COL3A1* and its collagen family members and *IGF1* further confirms the reliability of these data sets and these associations are well known [19,22]. For the mouse RI strains, evidently the data is accurate not only because of the homozygousity of the mouse strain but also for the repeatability of multiple mice within each strain [12]. Most of the differences between mice and humans are very clear cut. For example, there is a significant negative correlation between *Col3a1* and *Otud7b* and *Caskin2* in mice. However, in humans, probes of *OTUD7B* and *PTPLA* are not associated with *COL3A1*.

The strength of the signal in microarrays sometimes is influenced by polymorphisms that are located within the sequences of probes. In our study, differentially expressed genes are detected by multiple probes [12,27]. The chance of polymorphisms in multiple probes to have the same influence on the signal level is rare. Therefore, we feel confident of the results on the expression levels of these genes.

A clear limitation in our study is that the mouse RI strains are derived from two parental strains, the C57BL/6J and DBA/2J. Phenotypes of mouse models are known to be strain dependent [28,29]. The molecular pathways from these two strains may not necessarily be the same with that of other strains. Nevertheless, these two strains are amongst the most popular strains used for the study of human diseases. For example, both Tsk1 and Tsk2/+ mouse models for study of human SSc are bred under B6 background [5,6,30]. The human population included data from more than 1000 individuals. Therefore, we believe that the data from this study reflects the important difference between mouse models and humans.

## 4. Experimental Section

### 4.1. Mouse Gene Expression Data Sets for Lung

Gene expression data from the mouse lung in recombinant inbred (RI) strains and standard inbred strains was done with Affy Mouse Genome 430 2.0 (GPL1261) [12] (http://www.genenetwork.org/). The data set contains the whole gene expression profiles from 61 mouse strains, including 47 RI strains from BXD (derived from C57BL/6J and DBA/2J), two parents and two F1s, and 10 standard inbred strains.

### 4.2. Human Gene Expression Data for Lung

Human expression data include two sets from microarray and RNAseq. One set is the Super Series GSE23546 Whole-Genome GXD Non-Tumorous Human Lung Tissues Affy HuRSTA array (RMA Database). This set data from lung includes whole gene expression profiles of 1230 human samples of normal lung tissues processed with Affymetrix HuRSTA array. The gene expression data are available through GSE23546 at the GeneNetwork (http://www.genenetwork.org/).

The whole-genome gene expression profiles of RNAseq data of human lung tissues in GeneNetwork were contributed by the Genotype-Tissue Expression (GTEx) project [14]. The data set of lung RNAseq currently includes RNA sequence data from 119 individuals from three data sets of whole-genome gene expression profiles of non-tumorous human lung tissues: Laval set (GSE23352), UBC set (GSE23529), and GRNG set (GSE23545). RNAseq was performed using the Illumina TruSeq library construction protocol.

### 4.3. eQTL Mapping

The GeneNetwork is capable of mapping the eQTL with conventional QTL methods, such as the Interval mapping in the GeneNetwork. Interval mapping is a process in which the statistical significance of a hypothetical QTL is evaluated at regular points across a chromosome, even in the absence of explicit genotype data at those points. In the case of eQTL, significance is calculated using an efficient and very rapid regression method: the Haley–Knott regression equations [15]. Differences in the expression of mRNA are treated as standard phenotypes. A simple regression method for mapping quantitative trait loci in line crosses using flanking markers [16,17] in which trait values are compared to the known genotype at a marker or to the probability of a specific genotype at a test location between two flanking markers (http://www.genenetwork.org).

### 4.4. Gene Network Construction

The gene network was constructed using tools in GeneNetwork. The gene network was constructed based on the Network Graph in combination with the Correlation Matrix. Network graph is often used to visualize multiple sets of interactions. It consists of nodes and edges connecting nodes. In addition, the network graph can represent the strength of the interaction by the thickness of the edge—that is, the higher the number of interactions between two nodes, the thicker the edge becomes. Different colors and lines were used to illustrate the nature and the strength of correlations between genes. Solid lines represent strong correlation (absolute value: thick line = 0.7–1; thin line = 0.5–0.7), while dashed lines represent weak correlation (absolute value: 0–0.5). Red, pink and yellow colors are for positive correlations, while blue, green and black colors for negative correlations. For the Correlation Matrix, GeneNetwork provides tools to compute both Pearson product-moment correlations (the standard type of correlation) and Spearman rank order correlations. Both the Network Graph and Correlation Matrix were obtained with the same set of parameters or criteria. For example, for the Line Threshold in the Network Graph, absolute values greater than 0.35 were used across all samples. The Spring Model layout (force reduction) and circular model were used for the graphic method for all graphic samples (http://www.genenetwork.org/).

In case of multiple probes, we first used all the probes in the construction of gene network. If they were all highly positively correlated, then one probe (usually the one with the highest expression level) was used for the final construction of gene network.

### 4.5. Statistical Analysis

The top genes shared by two sets of data from mice and two sets of humans on the basis of Pearson correlation, were used for plotting Network Graphs in GeneNetwork. An *r* absolute value >0.50 was considered to indicate connection line threshold (exception otherwise will be noted).

## Supplementary Materials

Supplementary materials can be found at http://www.mdpi.com/1422-0067/16/07/15031/s1.
