# Supplementary material for: Differences between Mice and Humans in Regulation and the Molecular Network of Collagen, Type III, Alpha-1 at the Gene Expression Level: Obstacles that Translational Research Must Overcome
Source: Int J Mol Sci. 2015 Jul 3;16(7):15031–56. doi: 10.3390/ijms160715031 (PMC4519886; doi:10.3390/ijms160715031)
Supplement: Supplementary file 1 [file ijms-16-15031-s001.pdf]

Supplementary Information

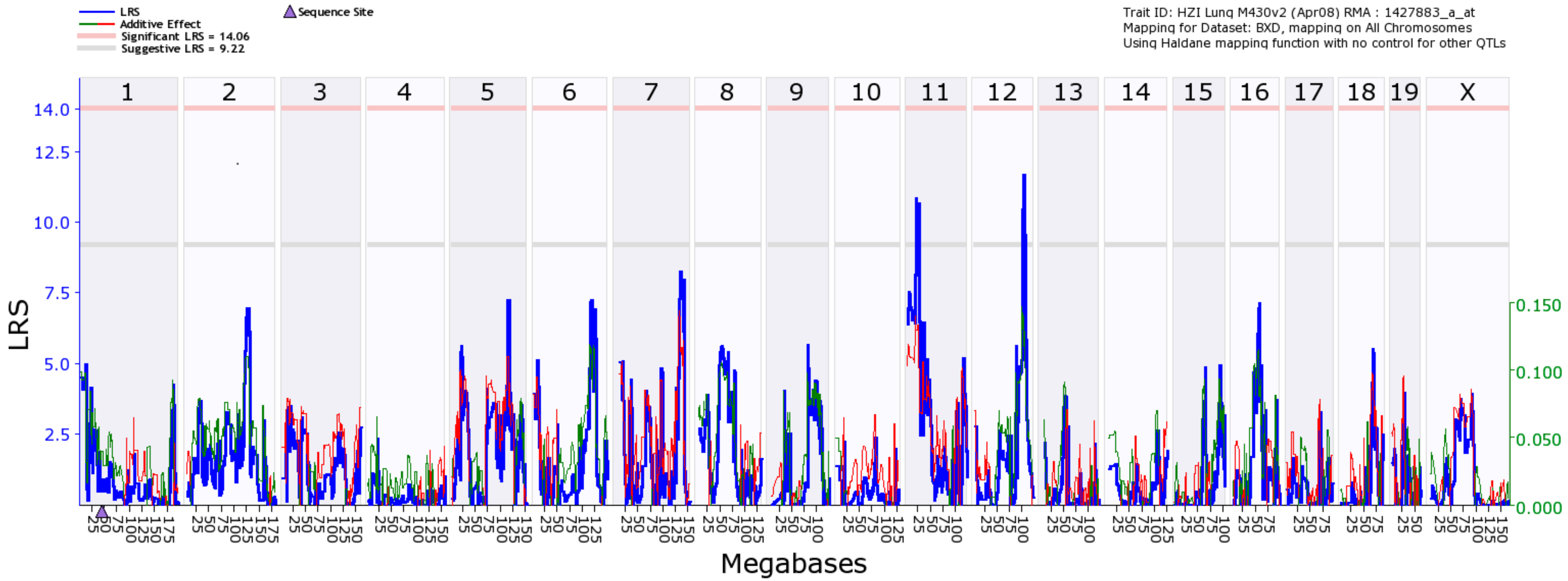

(A)

Figure S1. *Cont.*

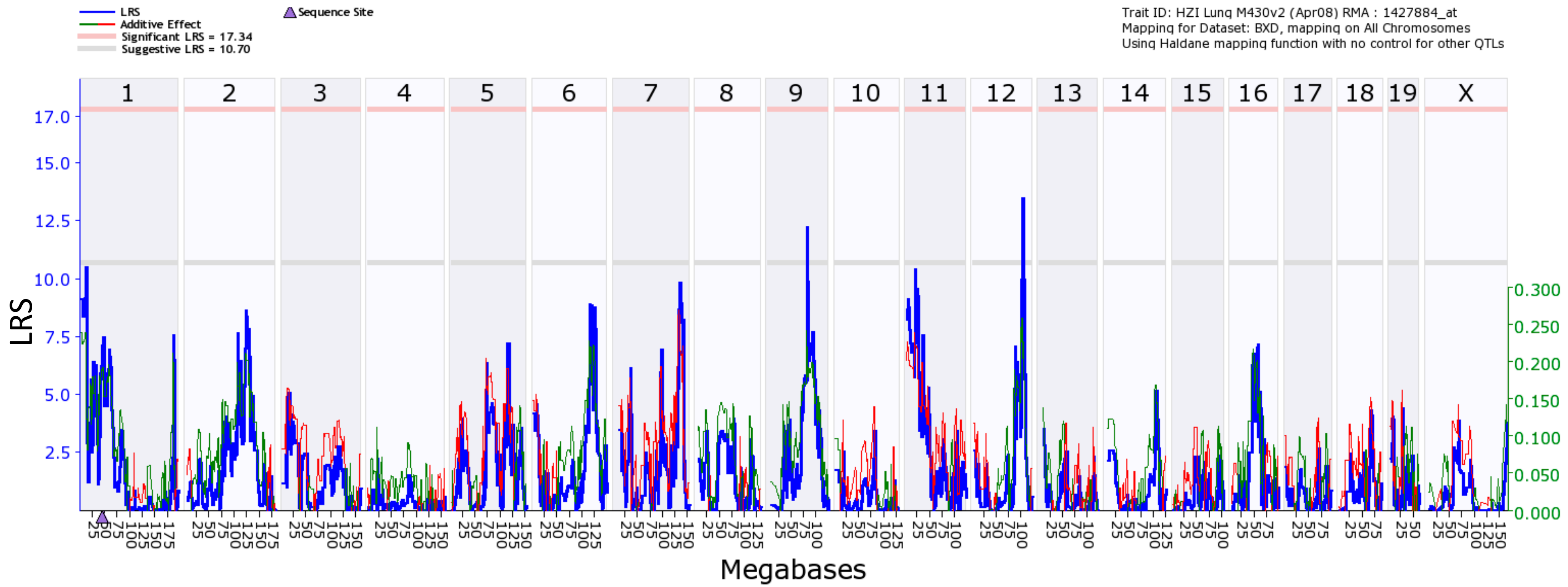

(B)

Figure S1. *Cont.*

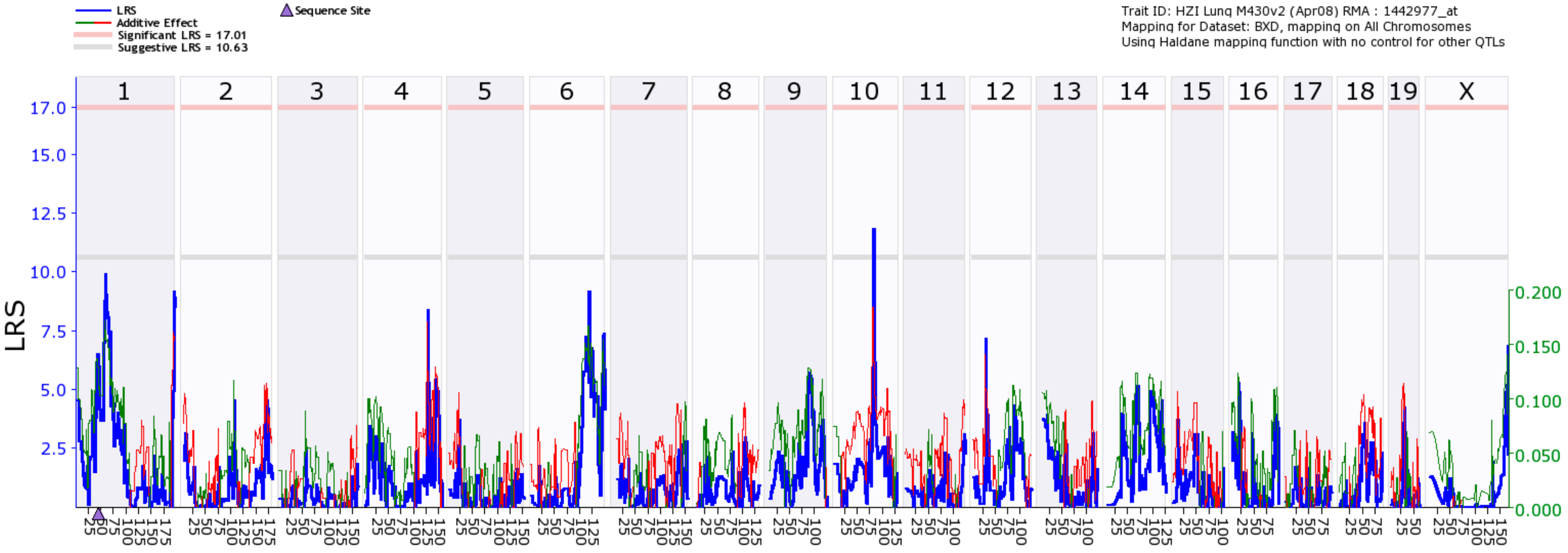

(**C**)

Figure S1. (**A**) eQTL of *Col3a1* mapped based on probe #1427883 using data of HZI Lung M430v2 (Apr08) RMA Database; (**B**) eQTL of *Col3a1* mapped based on probe #1427884 using data of HZI Lung M430v2 (Apr08) RMA Database; (**C**) eQTL of *Col3a1* mapped based on probe #1442977 using data of HZI Lung M430v2 (Apr08) RMA Database.

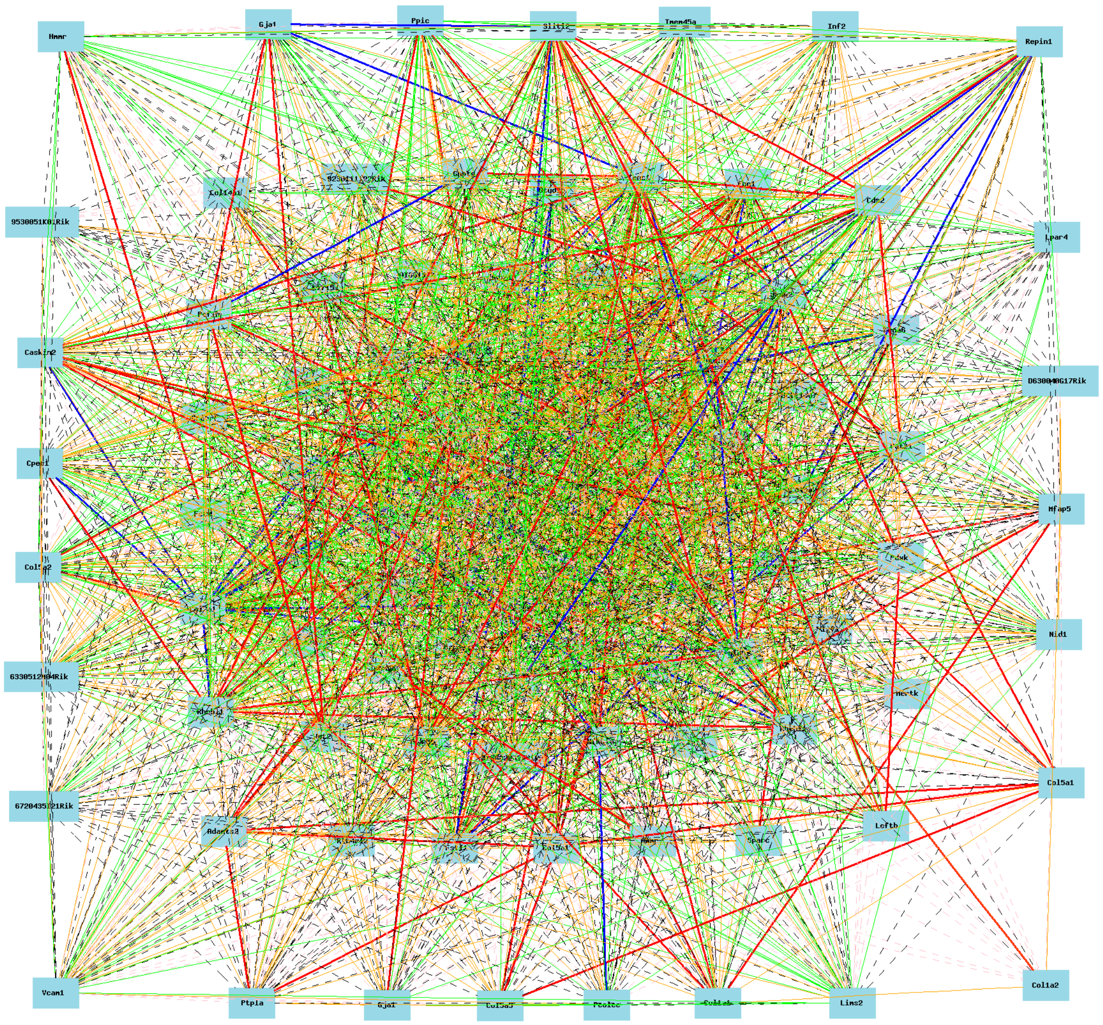

Figure S2. Gene network of top 100 *Col3a1* relevant genes from data of HZI Lung M430v2 (Apr08) RMA Database using probe 1427883. The 100 nodes in the graph below show the selected traits. Only nodes with edges are displayed. The 3756 edges between the nodes, filtered from the 4950 total edges and drawn as lines, show Pearson correlation coefficients greater than 0.35 or less than −0.35. The graph's canvas is 40.0 by 40.0 cm, and the node labels are drawn with a 10.0 point font, and the edge labels are drawn with a 10.0 point font.

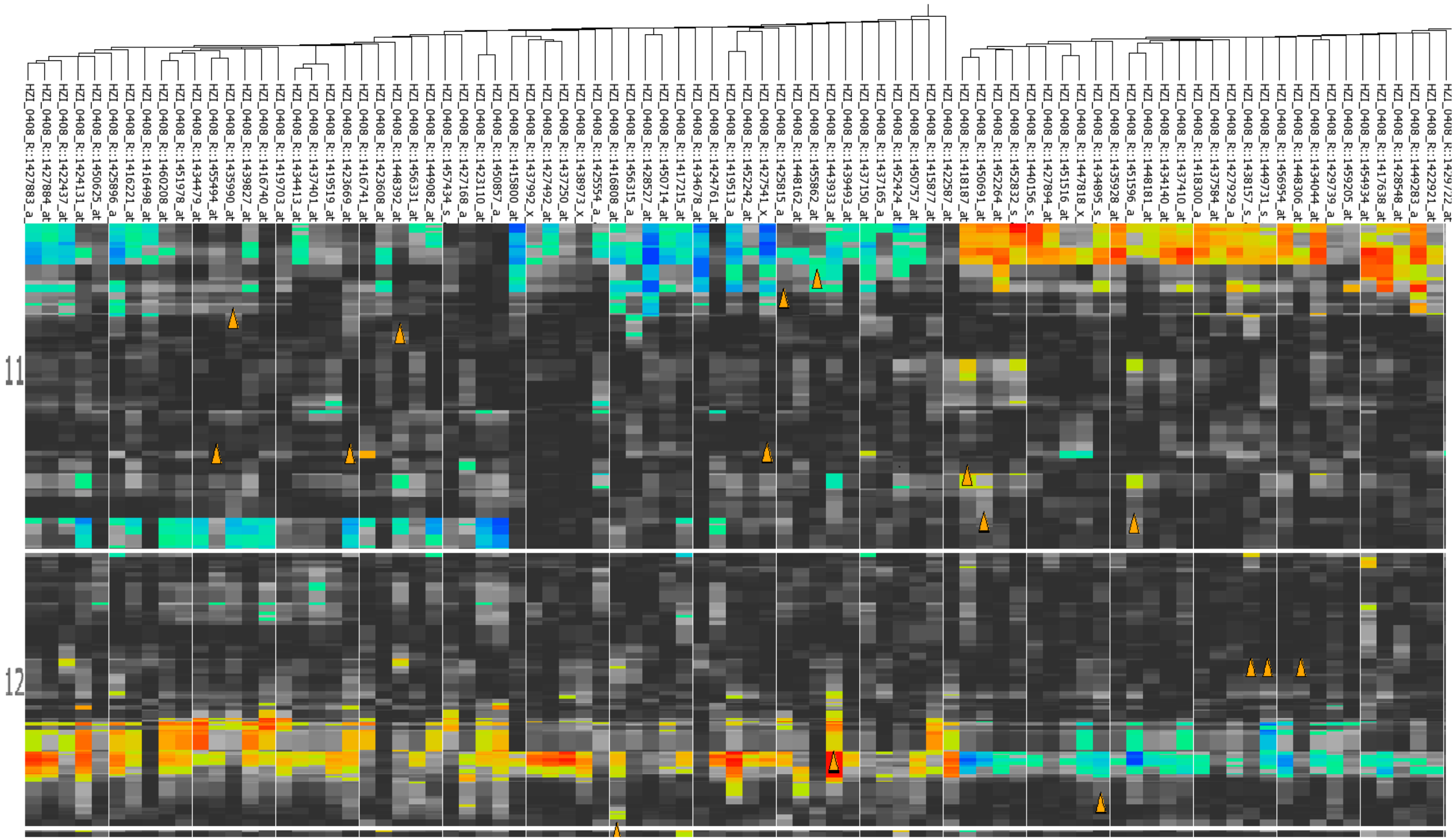

Figure S3. Heatmap of top 100 *Col3a1* relevant genes from data of HZI Lung M430v2 (Apr08) RMA Database. The upper part of this page includes a hierarchical cluster tree of the set of 100 genes. GeneNetwork computes distances between pairs of traits using $(1 - r)$ where r is the Pearson product-moment correlation. The lower part of this page provides a QTL heat map for all 100 members of the Cluster Tree, extending from proximal Chr 1 at the top to distal Chr X at the bottom. Each vertical column or stripe encodes the genome-wide p value computed on the basis of 1000 permutations. Orange triangles mark the approximate location of genes. Blue-green regions are those in which C57BL/6J is associated with higher trait values, whereas red-yellow regions are those in which DBA/2 allele is associated with higher trait values. Grey and black regions have insignfiicant linkage to trait variance.

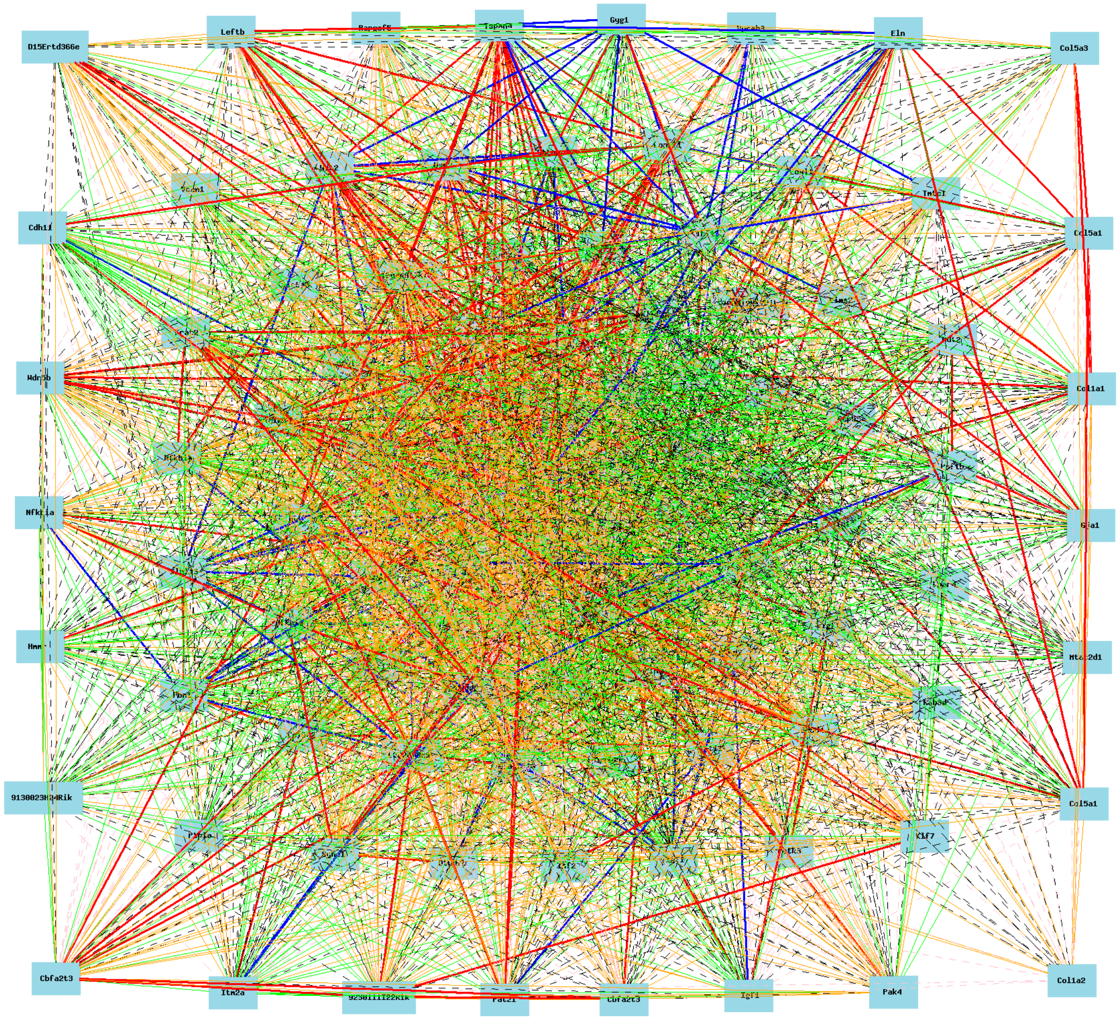

Figure S4. Gene network of top 100 *Col3a1* relevant genes from data of HZI Lung M430v2 (Apr08) RMA Database using probe 1427884. The 100 nodes in the graph below show the selected traits. Only nodes with edges are displayed. The 4317 edges between the nodes, filtered from the 4950 total edges and drawn as lines, show Pearson correlation coefficients greater than 0.35 or less than −0.35. The graph's canvas is 40.0 by 40.0 cm, and the node labels are drawn with a 10.0 point font, and the edge labels are drawn with a 10.0 point font.

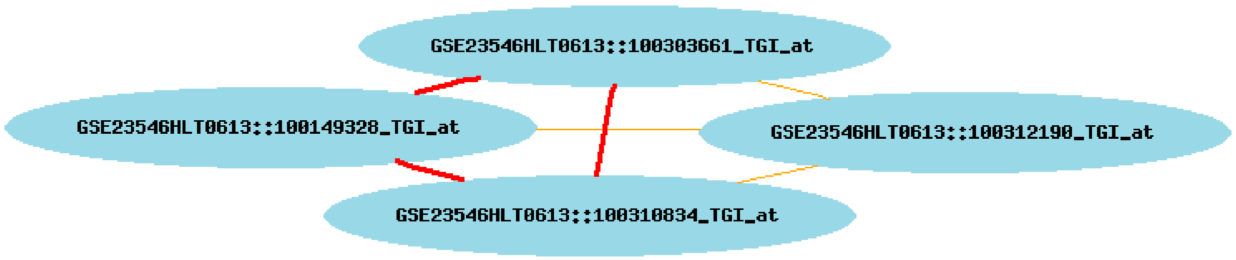

Figure S5. Gene network of four probes of *COL3A1* in human microarray data. The expression levels of three probes 100303661, 100149328, and 100310834 are strongly correlated while the expression of probe 100312190 is not closely correlated to any of these three probes.

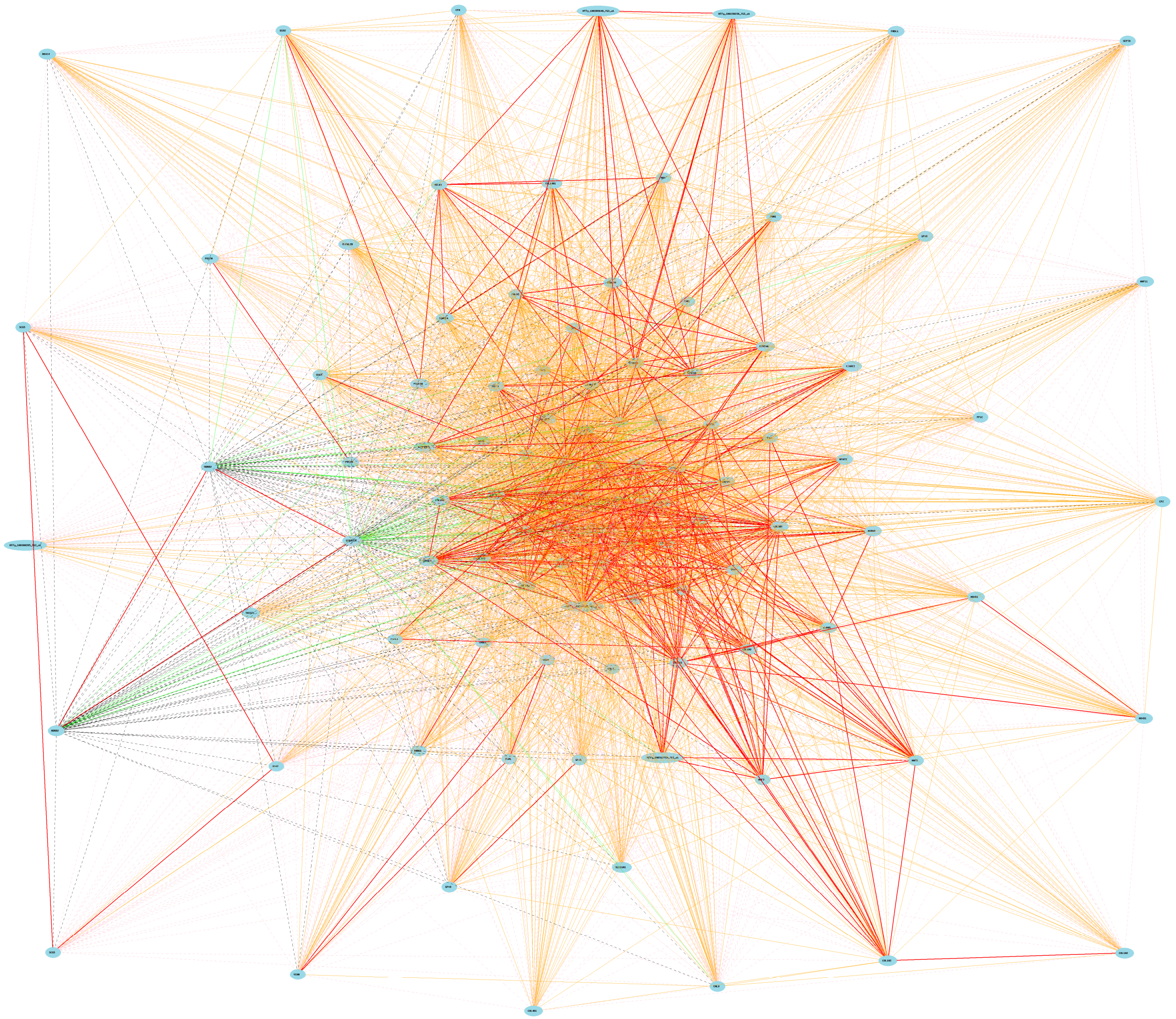

(**A**)

Figure 6. *Cont.*

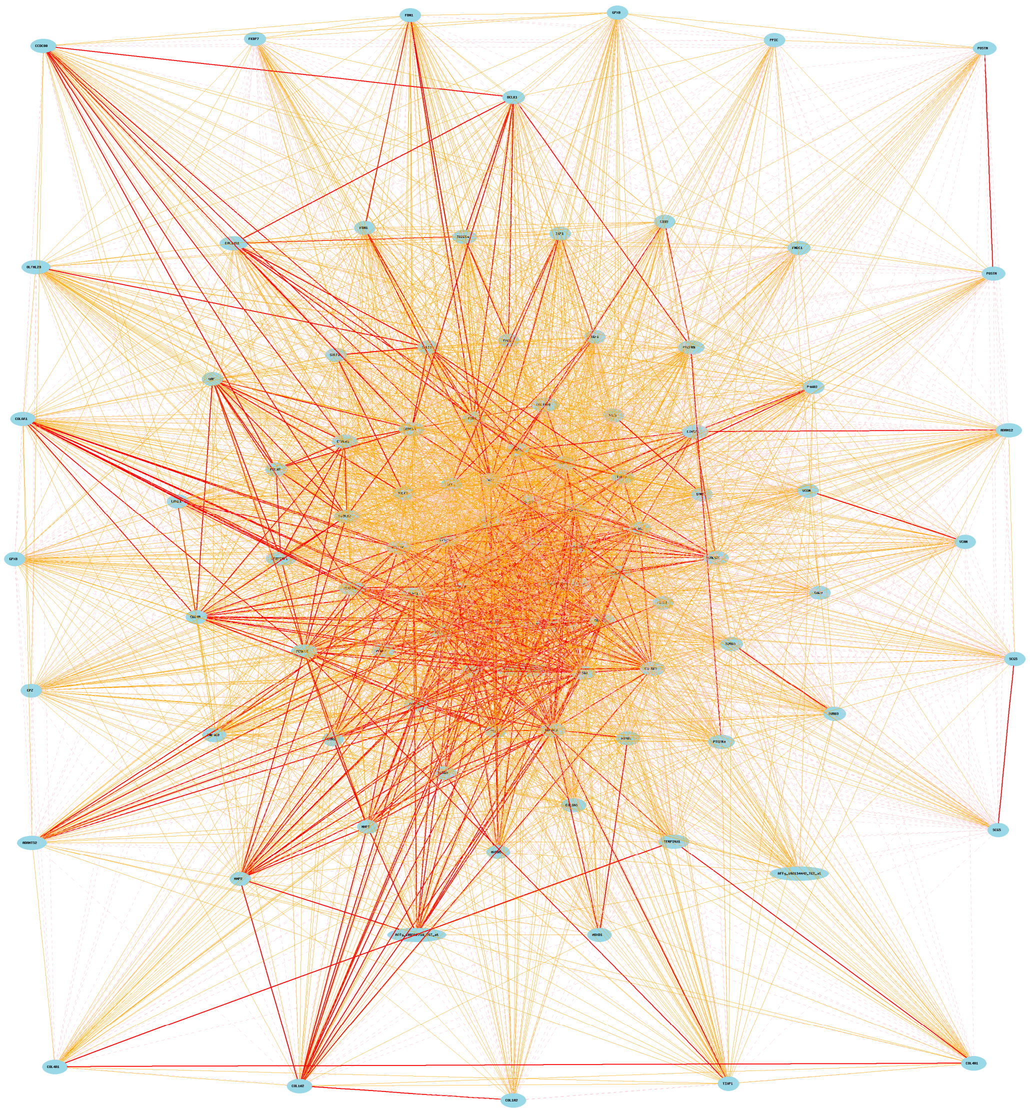

(B)

Figure 6. *Cont.*

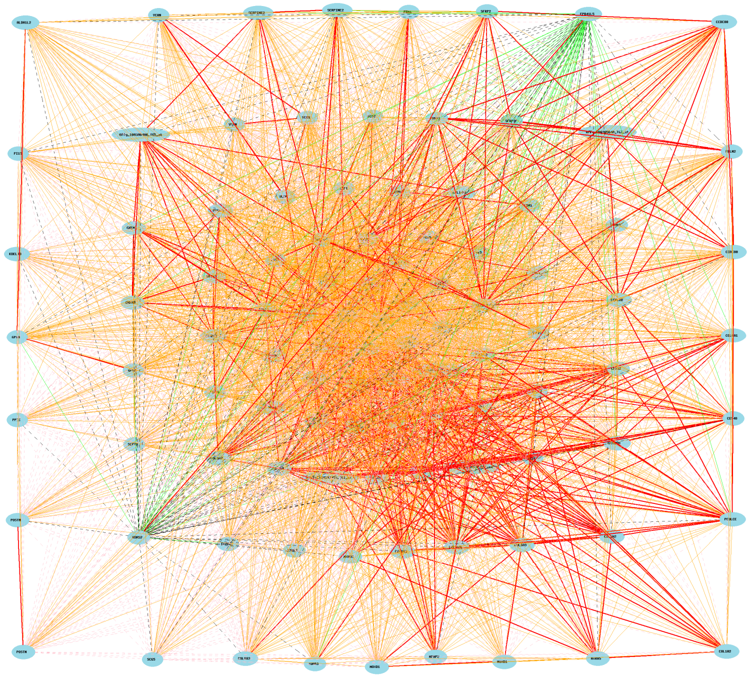

(**C**)

Figure S6. (**A**) Gene network of top 100 probe of *COL3A1* based on human microarray probe 100303661. The 100 nodes in the graph below show the selected traits. Only nodes with edges are displayed. The 4387 edges between the nodes, filtered from the 4950 total edges and drawn as lines, show Pearson correlation coefficients greater than 0.35 or less than −0.35. The graph's canvas is 40.0 by 40.0 cm, and the node labels are drawn with a 10.0 point font, and the edge labels are drawn with a 10.0 point font; (**B**) Gene network of top 100 probe of *COL3A1* based on human microarray probe 100149328. The 100 nodes in the graph below show the selected traits. Only nodes with edges are displayed. The 4465 edges between the nodes, filtered from the 4950 total edges and drawn as lines, show Pearson correlation coefficients greater than 0.35 or less than −0.35. The graph's canvas is 40.0 by 40.0 cm, and the node labels are drawn with a 10.0 point font, and the edge labels are drawn with a 10.0 point font; (**C**) Gene network of top 100 probe of *COL3A1* based on human microarray probe 100310834. The 100 nodes in the graph below show the selected traits. Only nodes with edges are displayed. The 4605 edges between the nodes, filtered from the 4950 total edges and drawn as lines, show Pearson correlation coefficients greater than 0.35 or less than −0.35. The graph's canvas is 40.0 by 40.0 cm, and the node labels are drawn with a 10.0 point font, and the edge labels are drawn with a 10.0 point font.

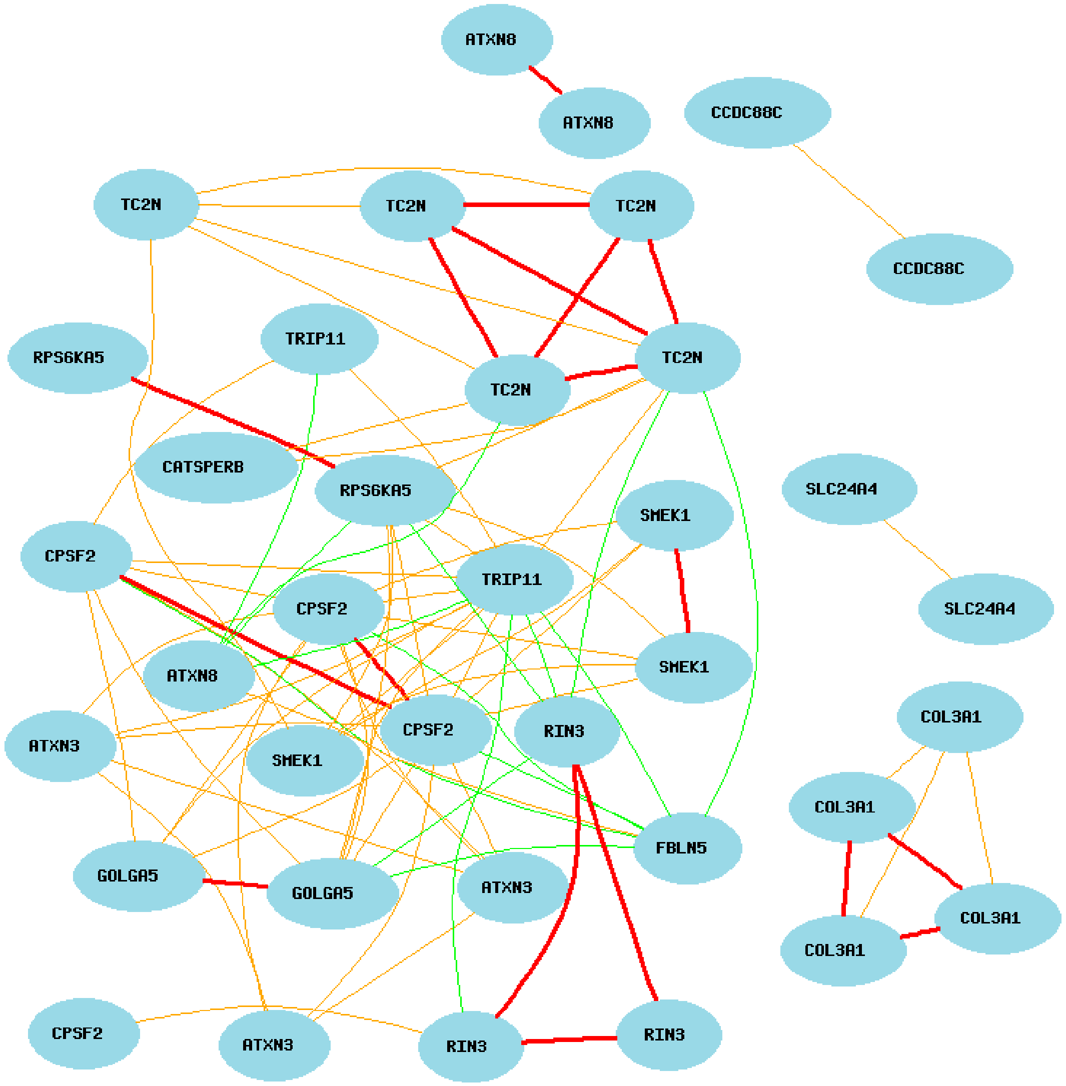

(A)

Figure 7. *Cont.*

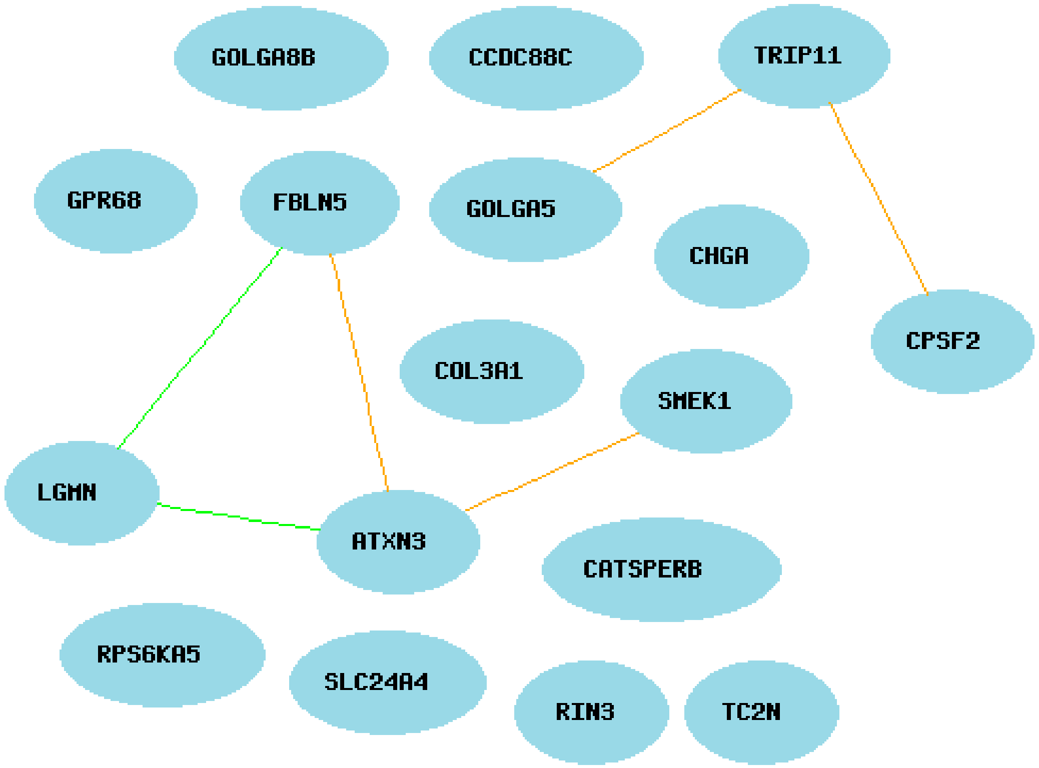

(**B**)

Figure S7. (**A**) None association of probes of 16 candidate genes on chr12 from mice in human microarray data. The 46 nodes in the graph below show the selected traits. Only nodes with edges are displayed. The 86 edges between the nodes, filtered from the 1035 total edges and drawn as curves, show Pearson correlation coefficients greater than 0.5 or less than −0.5. The graph's canvas is 40.0 by 40.0 cm, and the node labels are drawn with a 10.0 point font, and the edge labels are drawn with a 10.0 point font; (**B**) None association of probes of 16 candidate genes on chr12 from mice in human RNAseq data. The 17 nodes in the graph below show the selected traits. All nodes are displayed. The 6 edges between the nodes, filtered from the 136 total edges and drawn as curves, show Pearson correlation coefficients greater than 0.5 or less than −0.5. The graph's canvas is 40.0 by 40.0 cm, and the node labels are drawn with a 10.0 point font, and the edge labels are drawn with a 10.0 point font.

Table S1. Genetic elements within genomic region of eQTL on mouse Chr 12 detected with probe 1427883 of *Col3a1*.

Index	Symbol	Mb Start (mm9)	Length (Kb)	SNP Count	SNP Density	Human Chr	Mb Start (hg19)	Gene Description
1	Glac	99.440509	57.038	0	0	14	87.469110	galactosylceramidase
2	Gpr65	99.506866	7.976	0	0	14	87.541248	G-protein coupled receptor
3	Kcnk10	99.672203	143.947	4	0.027788	14	87.720999	potassium channel, subfami
4	5330409N07Rik	99.683738	2.806	0	0	–	–	RIKEN cDNA 5330409N07 gene
5	Spata7	99.866393	41.631	2	0.048041	14	87.921763	spermatogenesis associated
6	Ptpn21	99.914950	60.665	157	2.587983	14	88.003869	protein tyrosine phosphata
7	Zc3h14	99.985177	40.805	21	0.514643	–	–	zinc finger CCCH type cont
8	9430031K09Rik	100.000257	0.925	2	2.162162	–	–	RIKEN cDNA 9430031K09 gene
9	D230049E03Rik	100.004314	4.121	0	0	–	–	RIKEN cDNA D230049E03 gene
10	Eml5	100.024813	114.881	170	1.479792	14	88.150956	echinoderm microtubule ass
11	A930040O22Rik	100.070022	0.973	0	0	–	–	RIKEN cDNA A930040O22 gene
12	Ttc8	100.158783	62.659	189	3.016326	14	88.360249	tetratricopeptide repeat domain-containing protein 8
13	4930474N09Rik	100.399486	1.433	0	0	–	–	RIKEN cDNA 4930474N09 gene
14	Foxn3	100.433303	254.981	51	0.200015	–	–	forkhead box N3
15	3300002A11Rik	100.598080	2.158	0	0	–	–	RIKEN cDNA 3300002A11 gene
16	Ttc7b	100.702095	0.050	0	0	14	90.076685	tetratricopeptide repeat domain 7B
17	4930556H04Rik	100.807949	1.675	4	2.388060	–	–	RIKEN cDNA 4930556H04 gene
18	1700064M15Rik	100.864281	1.903	0	0	–	–	RIKEN cDNA 1700064M15 gene
19	2610021K21Rik	100.955740	165.912	257	1.549014	–	–	RIKEN cDNA 2610021K21 gene
20	Tdp1	101.122724	70.702	28	0.396028	14	89.491998	tyrosyl-DNA phosphodiester
21	LOC100040041	101.122828	0.528	0	0	–	–	hypothetical protein LOC10
22	Kcnk13	101.204117	96.769	332	3.430851	14	89.597860	potassium channel, subfami
23	4930477G07Rik	101.315117	20.311	7	0.344641	–	–	RIKEN cDNA 4930477G07 gene
24	9530050K03Rik	101.317542	0.431	1	2.320186	–	–	RIKEN cDNA 9530050K03 gene
25	Psmc1	101.350540	11.034	21	1.903208	14	89.792646	protease (prosome, macropa)
26	BC002230	101.363661	34.202	199	5.818373	–	–	cDNA sequence BC002230
27	LOC320288	101.395480	3.153	18	5.708849	–	–	hypothetical LOC320288
28	Calm1	101.437750	10.266	14	1.363725	–	–	calmodulin 1

Table S1. *Cont.*

Index	Symbol	Mb Start (mm9)	Length (Kb)	SNP Count	SNP Density	Human Chr	Mb Start (hg19)	Gene Description
29	Rps6ka5	101.787987	175.251	381	2.174025	14	90.406924	ribosomal protein S6 kinas
30	9030617O03Rik	102.017332	93.488	240	2.567174	–	–	RIKEN cDNA 9030617O03 gene
31	Gpr68	102.114896	11.193	41	3.663004	14	90.769964	G protein-coupled receptor
32	Ccdc88c	102.150909	116.284	207	1.780125	–	–	coiled-coil domain contain
33	Smek1	102.277618	44.294	64	1.444891	–	–	SMEK homolog 1, suppressor
34	Trip11	102.290795	0.048	0	0	14	91.505613	thyroid hormone receptor i
35	D130020L05Rik	102.320722	1.733	5	2.885170	–	–	RIKEN cDNA D130020L05 gene
36	4930463M05Rik	102.387316	0.652	4	6.134969	–	–	RIKEN cDNA 4930463M05 gene
37	Catsperb	102.642891	221.267	564	2.548957	–	–	cation channel, sperm-asso
38	Tc2n	102.886811	62.972	221	3.509496	–	–	tandem C2 domains, nuclear
39	Fbln5	102.984774	72.491	47	0.648356	14	91.405508	fibulin 5
40	Atxn3	103.157763	38.629	6	0.155324	14	91.599682	ataxin 3
41	LOC100040305	103.212525	1.396	0	0	–	–	hypothetical protein LOC10
42	Cpsf2	103.214183	30.020	1	0.033311	14	91.658081	cleavage and polyadenylati
43	Slc24a4	103.367628	137.331	245	1.784011	14	91.858677	solute carrier family 24
44	Gm46	103.468769	0.050	0	0	–	–	gene model 46, (NCBI)
45	C030009J22Rik	103.508600	2.101	0	0	–	–	RIKEN cDNA C030009J22 gene
46	Rin3	103.521850	107.212	12	0.111928	14	92.049877	Ras and Rab interactor 3
47	E030047P09Rik	103.604268	1.066	0	0	–	–	RIKEN cDNA E030047P09 gene
48	Lgmn	103.632307	45.600	9	0.197368	14	92.239909	legumain
49	A930036A04Rik	103.678932	1.050	0	0	–	–	RIKEN cDNA A930036A04 gene
50	Golga5	103.708119	27.998	5	0.178584	14	92.330402	golgi autoantigen, golgin
51	Chga	103.793178	10.059	0	0	14	92.459244	chromogranin A
52	Itpk1	103.806792	136.287	10	0.073375	14	92.475959	inositol 1,3,4-triphosphat
53	Moap1	103.979926	16.956	3	0.176929	14	92.718303	modulator of apoptosis 1
54	D230037D09Rik	103.981969	1.638	0	0	–	–	RIKEN cDNA D230037D09 gene
55	5730410I19Rik	103.996184	19.727	0	0	–	–	RIKEN cDNA 5730410I19 gene

Table S1. *Cont.*

Index	Symbol	Mb Start (mm9)	Length (Kb)	SNP Count	SNP Density	Human Chr	Mb Start (hg19)	Gene Description
56	Btbd7	104.022857	93.759	430	4.586226	14	92.773650	BTB (POZ) domain containin
57	Cox8c	104.137515	1.229	3	2.441009	14	92.883289	cytochrome c oxidase, subu

–: Information are not available.

Table S2. Genetic elements within genomic region of eQTL on mouse Chr 12 detected with probe 1427884 of *Col3a1*.

Index	Symbol	Mb Start (mm9)	Length (Kb)	SNP Count	SNP Density	Human Chr	Mb Start (hg19)	Gene Description
1	Rps6ka5	101.787987	175.251	381	2.174025	14	90.406924	ribosomal protein S6 kinas
2	9030617O03Rik	102.017332	93.488	240	2.567174	–	–	RIKEN cDNA 9030617O03 gene
3	Gpr68	102.114896	11.193	41	3.663004	14	90.769964	G protein-coupled receptor
4	Ccdc88c	102.150909	116.284	207	1.780125	–	–	coiled-coil domain contain
5	Smek1	102.277618	44.294	64	1.444891	–	–	SMEK homolog 1, suppressor
6	Trip11	102.290795	0.048	0	0	14	91.505613	thyroid hormone receptor i
7	D130020L05Rik	102.320722	1.733	5	2.885170	–	–	RIKEN cDNA D130020L05 gene
8	4930463M05Rik	102.387316	0.652	4	6.134969	–	–	RIKEN cDNA 4930463M05 gene
9	Catsperb	102.642891	221.267	564	2.548957	–	–	cation channel, sperm-asso
10	Tc2n	102.886811	62.972	221	3.509496	–	–	tandem C2 domains, nuclear
11	Fbln5	102.984774	72.491	47	0.648356	14	91.405508	fibulin 5
12	Atxn3	103.157763	38.629	6	0.155324	14	91.599682	ataxin 3
13	LOC100040305	103.212525	1.396	0	0	–	–	hypothetical protein LOC10
14	Cpsf2	103.214183	30.020	1	0.033311	14	91.658081	cleavage and polyadenylati
15	Slc24a4	103.367628	137.331	245	1.784011	14	91.858677	solute carrier family 24
16	Gm46	103.468769	0.050	0	0	–	–	gene model 46, (NCBI)
17	C030009J22Rik	103.508600	2.101	0	0	–	–	RIKEN cDNA C030009J22 gene
18	Rin3	103.521850	107.212	12	0.111928	14	92.049877	Ras and Rab interactor 3
19	E030047P09Rik	103.604268	1.066	0	0	–	–	RIKEN cDNA E030047P09 gene
20	Lgmn	103.632307	45.600	9	0.197368	14	92.239909	legumain
21	A930036A04Rik	103.678932	1.050	0	0	–	–	RIKEN cDNA A930036A04 gene

Table S2. *Cont.*

Index	Symbol	Mb Start (mm9)	Length (Kb)	SNP Count	SNP Density	Human Chr	Mb Start (hg19)	Gene Description
22	Golga5	103.708119	27.998	5	0.178584	14	92.330402	golgi autoantigen, golgin
23	Chga	103.793178	10.059	0	0	14	92.459244	chromogranin A

–: Information are not available.

Table S3. Top 100 probes their expression levels are highly correlated to the expression of *Col3a1* using data of HZI Lung M430v2 (Apr08) RMA Database.

Record ID	Gene ID	Homologene ID	Symbol	Description	Location (Chr: Mb)	Mean Expr	Max LRS	Max LRS Location (Chr: Mb)	Sample Rho	*N* Cases	Sample P(rho)	Lit Corr	Tissue Rho	Tissue P(rho)
1427883_a_at	12825	55433	Col3a1	procollagen, type 3, alpha 1 (Ehlers-Danlos syndrome types IV, aortic and arterial aneurysms)	Chr1: 45.404600	13.32673684	11.7	Chr12: 101.866283	1	49	0	1	1	8.88415×10^{-8}
1427884_at	12825	55433	Col3a1	procollagen, type III, alpha 1 (Ehlers-Danlos syndrome types IV, aortic and arterial aneurysms)	Chr1: 45.405965	10.13378947	13.5	Chr12: 101.866283	0.926020408	49	0	1	1	8.88415×10^{-8}
1422437_at	12832	20119	Col5a2	procollagen, type V, alpha 2	Chr1: 45.432766	11.33133333	12.8	Chr6: 9.485705	0.870816327	49	0	0.87	0.870769231	1.83671×10^{-6}
1423110_at	12843	69	Col1a2	procollagen, type 1, alpha 2	Chr6: 4.490998	11.05824561	10.2	Chr11: 114.532621	0.862117347	49	0	0.877	0.867350427	1.85681×10^{-6}
1460208_at	14118	30958	Fbn1	fibrillin 1 (Marfan syndrome)	Chr2: 125.126430	11.54631579	12.2	Chr16: 45.072728	0.860331633	49	0	0.61	0.884444444	1.71952×10^{-6}
1425896_a_at	14118	30958	Fbn1	fibrillin 1	Chr2: 125.127175	10.48815789	10.8	Chr11: 20.853303	0.808469388	49	2.70894×10^{-14}	0.61	0.884444444	1.71952×10^{-6}

Table S3. *Cont.*

Record ID	Gene ID	Homologene ID	Symbol	Description	Location (Chr: Mb)	Mean Expr	Max LRS	Max LRS Location (Chr: Mb)	Sample Rho	*N* Cases	Sample P(rho)	Lit Corr	Tissue Rho	Tissue P(rho)
1455494_at	12842	73874	Col1a1	procollagen, type I, alpha 1 (osteogenesis imperfecta types I–IV, Ehlers-Danlos syndrome type VIIA, Ehlers-Danlos syndrome classical type, Caffey Disease, idiopathic osteoporosis)	Chr11: 94.814020	10.11817544	10.4	Chr18: 68.674127	0.804744898	49	4.70735×10^{-14}	0.81	0.717606838	5.82788×10^{-5}
1439827_at	239337	12808	Adamts12	a disintegrin-like and metalloprotease with thrombospondin type 1 motif, 12	Chr15: 11.278497	8.218105263	15	Chr9: 80.917762	0.797984694	49	1.25233×10^{-13}	0.472	0.773675214	6.84245×10^{-6}
1434413_at	16000	515	Igf1	insulin-like growth factor 1 (somatomedin C)	Chr10: 87.399205	9.974526316	11.9	Chr6: 124.006511	0.787933673	49	4.92273×10^{-13}	0.418	0.305299145	0.129359367
1450625_at	12832	20119	Col5a2	procollagen, type V, alpha 2	Chr1: 45.431498	9.820280702	27.3	Chr1: 43.500859	0.78372449	49	8.50875×10^{-13}	0.87	0.870769231	1.83671×10^{-6}
1437401_at	16000	515	Igf1	insulin-like growth factor 1 (somatomedin C)	Chr10: 87.396551	9.840754386	13.2	Chr6: 117.050638	0.759311224	49	1.5532×10^{-11}	0.418	0.305299145	0.129359367
1416221_at	14314	5144	Fstl1	follistatin-like 1	Chr16: 37.835326	11.57580702	15.6	Chr6: 114.213342	0.75502551	49	2.47848×10^{-11}	0.563	0.793504274	3.38062×10^{-6}
1451978_at	16949	4074	Loxl1	lysyl oxidase-like 1	Chr9: 58.136428	11.50540351	12.7	Chr16: 45.072728	0.728571429	49	3.50001×10^{-10}	0.646	0.727863248	4.05629×10^{-5}
1434479_at	12831	55434	Col5a1	procollagen, type V, alpha 1	Chr2: 27.894230	9.543754386	14.9	Chr18: 69.067889	0.722678571	49	6.00546×10^{-10}	0.892	0.790769231	3.67995×10^{-6}
1418187_at	54409	4274	Ramp2	receptor (calcitonin) activity modifying protein 2	Chr11: 101.108917	13.05791228	15.5	Chr12: 101.866283	0.713647959	49	1.33122×10^{-9}	0.333	0.595213675	0.001644143

Table S3. *Cont.*

Record ID	Gene ID	Homologene ID	Symbol	Description	Location (Chr: Mb)	Mean Expr	Max LRS	Max LRS Location (Chr: Mb)	Sample Rho	*N* Cases	Sample P(rho)	Lit Corr	Tissue Rho	Tissue P(rho)
1419519_at	16000	515	Igf1	insulin-like growth factor 1 (somatomedin C)	Chr10: 87.393985	8.584631579	10.9	Chr5: 119.758226	0.712984694	49	1.40935×10^{-9}	0.418	0.305299145	0.129359367
1435990_at	216725	8597	Adamts2	a disintegrin-like and metalloproteinase with thrombospondin type 1 motif, 2 (procollagen type 1 and 2 *N*-proteinase, Ehlers-Danlos syndrome type VIIC)	Chr11: 50.620520	9.124964912	16.1	Chr18: 69.067889	0.7125	49	1.46915×10^{-9}	0.781	0.777777778	5.83708×10^{-6}
1416808_at	18073	1878	Nid1	nidogen 1	Chr13: 13.603982	11.54373684	12.8	Chr6: 126.754346	0.711632653	49	1.58216×10^{-9}	0.623	0.838632479	1.92381×10^{-6}
1423669_at	12842	73874	Col1a1	procollagen, type 1, alpha 1 (osteogenesis imperfecta types I-IV, Ehlers-Danlos syndrome type VIIA, Ehlers-Danlos syndrome classical type, Caffey Disease, idiopathic osteoporosis)	Chr11: 94.812511	11.68452632	12.6	Chr11: 116.908502	0.702653061	49	3.34484×10^{-9}	0.81	0.717606838	5.82788×10^{-5}
1424131_at	12835	37917	Col6a3	procollagen, type 6, alpha 3 (Bethlem myopathy)	Chr1: 92.663601	11.82814035	10.6	Chr11: 114.532621	0.699566327	49	4.29385×10^{-9}	0.775	0.828376068	1.96459×10^{-6}
1455239_at	320802	–	6330512M04Rik	RIKEN cDNA 6330512M04 gene	Chr7: 149.541434	8.935631579	13.4	Chr11: 26.370844	−0.698104544	49	4.82667×10^{-9}	0	−0.140536846	0.493490632
1449082_at	50530	2599	Mfap5	microfibrillar associated protein 5	Chr6: 122.478725	9.586210526	12.7	Chr11: 114.532621	0.68	49	1.87317×10^{-8}	0.508	0.817575666	3.41772×10^{-7}
1449731_s_at	18035	7863	Nfkbia	nuclear factor of kappa light chain gene enhancer in B-cells inhibitor, alpha	Chr12: 56.590517	11.94147368	13.4	Chr12: 87.446646	−0.679438776	49	1.95147×10^{-8}	0.403	0.315555556	0.116496589

Table S3. *Cont.*

Record ID	Gene ID	Homologene ID	Symbol	Description	Location (Chr: Mb)	Mean Expr	Max LRS	Max LRS Location (Chr: Mb)	Sample Rho	*N* Cases	Sample P(rho)	Lit Corr	Tissue Rho	Tissue P(rho)
1419513_a_at	13605	7298	Ect2	ect2 oncogene	Chr3: 26.996196	6.402894737	17.4	Chr12: 101.866283	0.679183673	49	1.98806×10^{-8}	0.341	−0.262222222	0.194935906
1451596_a_at	20698	39748	Sphk1	sphingosine kinase 1	Chr11: 116.397453	10.09082456	18.9	Chr12: 101.866283	−0.674795918	49	2.7273×10^{-8}	0.438	0.323760684	0.106914267
1422921_at	22364	7598	Vpreb3	pre-B lymphocyte gene 3	Chr10: 75.411885	9.317859649	10.4	Chr5: 23.471050	−0.672959184	49	3.10736×10^{-8}	0.336	−0.122051282	0.551014853
1452424_at	78134	3871	Lpar4	lysophosphatidic acid receptor 4	ChrX: 104.126532	6.065596491	23.3	Chr11: 4.408731	0.669362245	49	3.99925×10^{-8}	0.373	0.745641026	2.083×10^{-5}
1435928_at	234797	8825	Kiaa0513	KIAA0513 protein (neuroplasticity associated)	Chr8: 122.688606	9.620035088	14.3	Chr11: 24.572748	−0.668545918	49	4.23254×10^{-8}	0.296	0	1
1452264_at	209039	37077	Tenc1	tensin like C1 domain-containing phosphatase	Chr15: 101.946167	11.68761404	12.7	Chr11: 11.016916	−0.665535714	49	5.20734×10^{-8}	0.57	0.379145299	0.056960869
1416740_at	12831	55434	Col5a1	procollagen, type V, alpha 1 (Ehlers-Danlos syndrome)	Chr2: 27.892525	10.25282456	13.9	Chr12: 83.974582	0.665178571	49	5.33599×10^{-8}	0.892	0.790769231	3.67995×10^{-6}
1423608_at	16431	31269	Itm2a	integral membrane protein 2A	ChrX: 104.592554	10.73319298	16.3	Chr5: 113.832344	0.65625	49	9.69998×10^{-8}	0.498	0.402393162	0.042528236
1416741_at	12831	55434	Col5a1	procollagen, type V, alpha 1	Chr2: 27.893658	8.612017544	11	Chr16: 51.051259	0.653545918	49	1.15712×10^{-7}	0.892	0.790769231	3.67995×10^{-6}
1448392_at	20692	31132	Sparc	secreted acidic cysteine rich glycoprotein	Chr11: 55.208055	13.3972807	18.8	Chr6: 9.485705	0.652346939	49	1.25042×10^{-7}	0.694	0.617777778	0.000986661
1438157_s_at	18035	7863	Nfkbia	nuclear factor of kappa light chain gene enhancer in B-cells inhibitor, alpha	Chr12: 56.590866	12.43996491	13.1	Chr11: 4.408731	−0.650918367	49	1.37073×10^{-7}	0.403	0.315555556	0.116496589
1437410_at	11669	55480	Aldh2	aldehyde dehydrogenase 2, mitochondrial	Chr5: 122.021169	10.14747368	15.5	Chr1: 11.505582	−0.649311224	49	1.51894×10^{-7}	0.388	0.05982906	0.771191642

Table S3. *Cont.*

Record ID	Gene ID	Homologene ID	Symbol	Description	Location (Chr: Mb)	Mean Expr	Max LRS	Max LRS Location (Chr: Mb)	Sample Rho	*N* Cases	Sample P(rho)	Lit Corr	Tissue Rho	Tissue P(rho)
1443933_at	74413	12560	Mtac2d1	membrane targeting (tandem) C2 domain containing 1	Chr12: 102.883731	9.013087719	62.5	Chr12: 101.866283	0.647882653	49	1.66308×10^{-7}	0.093	0.108376068	0.596847375
1450691_at	140721	32485	Caskin2	cask-interacting protein 2	Chr11: 115.660538	10.76840351	12.5	Chr12: 101.866283	−0.642780612	49	2.2886×10^{-7}	0.147	0.309401709	0.124094013
1451516_at	69159	5477	Rhebl1	Ras homolog enriched in brain like 1	Chr15: 98.708467	8.949789474	12.2	Chr9: 90.299275	−0.641913265	49	2.41457×10^{-7}	0.227	−0.052991453	0.796906099
1449283_at	29857	55705	Mapk12	mitogen-activated protein kinase 12	Chr15: 88.961205	10.60645614	15.1	Chr11: 35.501290	−0.641505102	49	2.47606×10^{-7}	0.365	0.511794872	0.008278946
1448162_at	22329	838	Vcam1	vascular cell adhesion molecule 1	Chr3: 115.813116	10.75026316	10.6	Chr1: 3.482275	0.635892857	49	3.48352×10^{-7}	0.451	−0.271111111	0.17980585
1434044_at	58887	22810	Repin1	replication initiator 1	Chr6: 48.548584	9.823105263	11.2	Chr11: 20.853303	−0.635076531	49	3.6584×10^{-7}	0.469	−0.074188034	0.717994585
1415800_at	14609	136	Gja1	gap junction membrane channel protein alpha 1	Chr10: 56.109688	11.15501754	16.5	Chr11: 8.736727	0.63252551	49	4.25891×10^{-7}	0.403	0.439316239	0.025759879
1448306_at	18035	7863	Nfkbia	nuclear factor of kappa light chain gene enhancer in B-cells inhibitor, alpha	Chr12: 56.591432	11.94278947	15.2	Chr5: 76.574921	−0.629107143	49	5.20772×10^{-7}	0.403	0.315555556	0.116496589
1448181_at	66277	8553	Klf15	Kruppel-like factor 15	Chr6: 90.424644	10.59536842	11.1	Chr6: 9.485705	−0.627755102	49	5.63448×10^{-7}	0.436	0.076239316	0.710493888
1428527_at	76561	22941	Snx7	sorting nexin 7	Chr3: 117.485056	9.894789474	19.5	Chr11: 24.572748	0.626479592	49	6.06663×10^{-7}	0	0.286837607	0.155108075

Table S3. *Cont.*

Record ID	Gene ID	Homologene ID	Symbol	Description	Location (Chr: Mb)	Mean Expr	Max LRS	Max LRS Location (Chr: Mb)	Sample Rho	*N* Cases	Sample P(rho)	Lit Corr	Tissue Rho	Tissue P(rho)
1446440_at	–	–	D830011E08Rik	0 day neonate lung cDNA, RIKEN full-length enriched library, clone: E030006O04 product: unknown EST, full insert sequence	Chr6: 92.613962	10.49980702	15.4	Chr5: 113.832344	−0.626403061	49	6.09352×10^{-7}	0	0	1
1437992_x_at	14609	136	Gja1	gap junction membrane channel protein alpha 1	Chr10: 56.110123	11.78289474	11.4	Chr6: 131.940470	0.625612245	49	6.37786×10^{-7}	0.403	0.439316239	0.025759879
1447818_x_at	69159	5477	Rhebl1	Ras homolog enriched in brain like 1	Chr15: 98.708276	8.328859649	9.6	Chr2: 130.881867	−0.625510204	49	6.41543×10^{-7}	0.227	−0.052991453	0.796906099
1456315_a_at	30963	69153	Ptpla	protein tyrosine phosphatase-like (proline instead of catalytic arginine), member A (3-hydroxyacyl-CoA dehydrates 1)	Chr2: 162.724687	10.33866667	15.8	Chr7: 36.124856	0.615561224	49	1.12428×10^{-6}	0.442	0.528888889	0.006126717
1417215_at	80718	20879	Rab27b	RAB27b, member RAS oncogene family	Chr18: 70.144095	8.126403509	18.3	Chr18: 69.689995	0.612270408	49	1.34682×10^{-6}	0.274	0.415384615	0.035843035
1424408_at	225341	41214	Lims2	LIM and senescent cell antigen like domains 2	Chr18: 32.117769	11.37566667	11.3	Chr9: 47.039894	−0.611658163	49	1.39248×10^{-6}	0.399	0.43042735	0.029191757
1416498_at	19038	727	Ppic	peptidylprolyl isomerase C	Chr18: 53.566316	11.14619298	11.5	Chr5: 113.832344	0.611403061	49	1.41192×10^{-6}	0.474	0.693675214	0.000127713
1457434_s_at	30963	69153	Ptpla	protein tyrosine phosphatase-like (proline instead of catalytic arginine), member A (3-hydroxyacyl-CoA dehydrates 1)	Chr2: 13.948499	10.48963158	13.4	Chr6: 113.995963	0.609617347	49	1.55521×10^{-6}	0.442	0.528888889	0.006126717

Table S3. *Cont.*

Record ID	Gene ID	Homologene ID	Symbol	Description	Location (Chr: Mb)	Mean Expr	Max LRS	Max LRS Location (Chr: Mb)	Sample Rho	*N* Cases	Sample P(rho)	Lit Corr	Tissue Rho	Tissue P(rho)
1435552_at	229603	10624	Otud7b	OTU domain containing 7B	Chr3: 95.960507	10.37605263	12.2	Chr18: 67.157701	−0.609464286	49	1.5681×10^{-6}	0.295	0.312820513	0.11982912
1424761_at	232748	17147	BC011487	cDNA sequence BC011487	Chr6: 42.573173	8.580403509	15.3	Chr5: 118.405615	0.609438776	49	1.57026×10^{-6}	0	0.391452991	0.048903035
1432184_a_at	70435	82406	Inf2	inverted formin 2 (formin inverted 2)	Chr12: 113.853235	8.642333333	12.5	Chr9: 105.676711	−0.608341837	49	1.66568×10^{-6}	0.201	0.29025641	0.150080576
1454934_at	68606	22828	Ppm1f	protein phosphatase 1F (PP2C domain containing)	Chr16: 16.926929	10.73592982	15.4	Chr11: 20.853303	−0.607270408	49	1.76404×10^{-6}	0.253	−0.137777778	0.500443845
1429739_a_at	56218	8636	Patz1	POZ (BTB) and AT hook containing zinc finger 1	Chr11: 3.208489	9.876122807	8.9	Chr2: 121.412768	−0.606581633	49	1.83007×10^{-6}	0.329	-0.045470085	0.82543596
1452242_at	74107	10019	Cep55	centrosomal protein 55	Chr19: 38.148423	7.502210526	10.8	Chr12: 100.818236	0.606020408	49	1.88556×10^{-6}	0.315	0.052991453	0.796906099
1440156_s_at	269389	13155	LOC269389	embryonic retinal HMG-box protein	Chr2: 163.149678	11.17026316	14.3	Chr11: 8.736727	−0.604234694	49	2.07264×10^{-6}	0.344	0	1
1437150_at	242297	17518	1700012H17Rik	RIKEN cDNA 1700012H17	Chr4: 5.726501	8.107719298	12.2	Chr11: 19.879675	0.602336795	49	2.29018×10^{-6}	0.152	0.131623932	0.51994893
1418300_a_at	17347	49674	Mknk2	MAP kinase-interacting serine/threonine kinase 2	Chr10: 80.128102	12.37989474	12.7	Chr11: 8.736727	−0.599285714	49	2.68454×10^{-6}	0.343	0.416752137	0.035191839
1450857_a_at	12843	69	Col1a2	procollagen, type 1, alpha 2	Chr6: 4.489494	12.49684211	9.2	Chr11: 114.532621	0.599158163	49	2.70232×10^{-6}	0.877	0.867350427	1.85681×10^{-6}
1439493_at	244530	–	D630040G17Rik	RIKEN cDNA D630040G17 gene	Chr8: 81.716670	8.895631579	14.1	Chr11: 4.408731	0.598367347	49	2.81498×10^{-6}	0	0.00034188	1
1437165_a_at	18542	1946	Pcolce	procollagen C-proteinase enhancer protein	Chr5: 138.046420	11.9377193	16.3	Chr10: 80.258108	0.598137755	49	2.8485×10^{-6}	0.757	0.514529915	0.007896969

Table S3. *Cont.*

Record ID	Gene ID	Homologene ID	Symbol	Description	Location (Chr: Mb)	Mean Expr	Max LRS	Max LRS Location (Chr: Mb)	Sample Rho	*N* Cases	Sample P(rho)	Lit Corr	Tissue Rho	Tissue P(rho)
1419703_at	53867	9253	Col5a3	procollagen, type V, alpha 3	Chr9: 20.574515	8.048280702	11.8	Chr9: 116.482254	0.597729592	49	2.90899×10^{-6}	0.761	0.606153846	0.001289425
1456954_at	16494	1684	Kcna6	potassium voltage-gated channel, shaker-related, subfamily, member 6	Chr6: 126.658583	6.822315789	13.2	Chr12: 103.658501	−0.596989796	49	3.02166×10^{-6}	0.321	0.241025641	0.234541387
1456331_at	319792	–	9130023H24Rik	RIKEN cDNA 9130023H24 gene	Chr7: 135.378234	8.665438596	10.8	Chr9: 95.050077	0.595229592	49	3.30613×10^{-6}	0	0	1
1437584_at	69544	41307	Wdr5b	WD repeat domain 5B	Chr17: 47.656287	7.817824561	10.4	Chr19: 10.157494	−0.593316327	49	3.64323×10^{-6}	0.297	0.019487179	0.925473078
1427509_at	214505	13047	Gnptg	*N*-acetylglucosamine-1-phosphotransferase, gamma subunit	Chr17: 25.379638	8.564210526	12.2	Chr12: 101.866283	−0.593163265	49	3.67152×10^{-6}	0.459	−0.467350427	0.017031478
1427541_x_at	15366	8271	Hmmr	hyaluronan mediated motility receptor (RHAMM)	Chr11: 92.536885	7.050631579	17.3	Chr11: 19.645819	0.59252551	49	3.7916×10^{-6}	0.526	−0.126837607	0.535373594
1425554_a_at	69957	2899	Cdc16	CDC16 cell division cycle 16 homolog (S. cerevisiae)	Chr8: 13.779240	10.77270175	14.3	Chr7: 124.952037	0.592295918	49	3.83571×10^{-6}	0.222	0.308717949	0.124960341
1417545_at	63873	11003	Trpv4	transient receptor potential cation channel, subfamily V, member 4	Chr5: 115.072343	8.184175439	53.7	Chr5: 113.832344	−0.591811224	49	3.93039×10^{-6}	0.411	0.583589744	0.002109818
1459205_at	12877	7278	Cpeb1	cytoplasmic polyadenylation element binding protein 1	Chr7: 88.577468	7.637350877	16.7	Chr1: 3.482275	−0.591709184	49	3.95059×10^{-6}	0.39	0.344957265	0.084912068
1420223_at	20403	22627	Itsn2	intersectin 2	Chr12: 56.496739	6.564245614	10.9	Chr9: 95.251198	−0.590204082	49	4.25996×10^{-6}	0.32	−0.200683761	0.324072658

Table S3. *Cont.*

Record ID	Gene ID	Homologene ID	Symbol	Description	Location (Chr: Mb)	Mean Expr	Max LRS	Max LRS Location (Chr: Mb)	Sample Rho	*N* Cases	Sample P(rho)	Lit Corr	Tissue Rho	Tissue P(rho)
1452832_s_at	110911	37854	Cds2	CDP-diacylglycerol synthase (phosphatidate cytidylyltransferase) 2	Chr2: 132.137365	11.48801754	17.2	Chr11: 11.016916	−0.589362245	49	4.44259×10^{-6}	0.359	−0.230769231	0.25552815
1415877_at	22240	20361	Dpysl3	dihydropyrimidinase-like 3	Chr18: 43.487992	8.67277193	10.2	Chr12: 91.397267	0.589183673	49	4.48224×10^{-6}	0.406	0.114529915	0.576018247
1440230_at	244152	41045	9530051K01Rik	RIKEN cDNA 9530051K01 gene	Chr7: 105.499354	7.763070175	37.8	Chr7: 105.272767	−0.589056122	49	4.51077×10^{-6}	0.363	0	1
1454967_at	77128	12672	Crebrf	CREB3 regulatory factor	Chr17: 26.912981	11.16222807	9.9	Chr8: 77.650954	−0.586964286	49	5.0031×10^{-6}	0.197	0	1
1417638_at	13590	49231	Leftb	left right determination factor 1 (endometrial bleeding associated factor)	Chr1: 182.867972	8.624175439	17	Chr1: 187.001839	−0.586913265	49	5.0157×10^{-6}	0.334	0	1
1434895_s_at	21981	9090	Ppp1r13b	protein phosphatase 1, regulatory (inhibitor) subunit 13B	Chr12: 113.066695	11.11117544	12.5	Chr2: 121.412768	−0.586902727	49	5.01831×10^{-6}	0.289	−0.265641026	0.189014906
1438973_x_at	14609	136	Gja1	gap junction membrane channel protein alpha 1	Chr10: 56.110073	6.946	14	Chr1: 65.557818	0.586862245	49	5.02833×10^{-6}	0.403	0.439316239	0.025759879
1427168_a_at	12818	18741	Col14a1	procollagen, type XIV, alpha 1	Chr15: 55.351777	10.3007193	11.1	Chr3: 126.116578	0.586683673	49	5.07278×10^{-6}	0.796	0.524786325	0.006594228
1455862_at	327900	15484	Ubtd2	ubiquitin domain containing 2	Chr11: 32.418126	8.808280702	14.5	Chr10: 80.258108	0.584158163	49	5.74142×10^{-6}	0.245	0.435213675	0.027300281
1429139_at	229603	10624	Otud7b	OTU domain containing 7B	Chr3: 95.964567	9.959859649	11.6	Chr16: 60.328963	−0.58380102	49	5.84226×10^{-6}	0.295	0.312820513	0.11982912
1428548_at	70584	4300	Pak4	p21 (CDKN1A)-activated kinase 4	Chr7: 29.343958	10.5287193	14.6	Chr15: 73.370668	−0.582423469	49	6.2467×10^{-6}	0.388	0.128205128	0.530944212
1439573_at	269295	18683	Rtn4rl2	reticulon 4 receptor-like 2	Chr2: 84.711512	7.795859649	14.5	Chr11: 16.853389	−0.581862245	49	6.41874×10^{-6}	0.294	0.001367755	0.994709113

Table S3. *Cont.*

Record ID	Gene ID	Homologene ID	Symbol	Description	Location (Chr: Mb)	Mean Expr	Max LRS	Max LRS Location (Chr: Mb)	Sample Rho	*N* Cases	Sample P(rho)	Lit Corr	Tissue Rho	Tissue P(rho)
1434140_at	17207	11804	Mcf2l	mcf.2 transforming sequence-like	Chr8: 13.020122	10.71173684	11.2	Chr11: 24.572748	−0.580153061	49	6.96991×10^{-6}	0.333	−0.063931624	0.755875442
1429588_at	77734	–	6720435I21Rik	protein LOC55196	Chr14: 56.858707	11.16584211	13.3	Chr4: 34.041018	−0.579234694	49	7.28373×10^{-6}	0	0	1
1450757_at	12552	1361	Cdh11	cadherin 11	Chr8: 105.157629	11.29538596	11.8	Chr19: 10.249020	0.578239796	49	7.63834×10^{-6}	0.54	0.128888889	0.528736191
1422587_at	56277	41215	Tmem45a	transmembrane protein 45a	Chr16: 56.806532	9.58777193	22.9	Chr16: 56.463019	0.578112245	49	7.68493×10^{-6}	0.273	0.660854701	0.000332102
1427929_a_at	216134	2731	Pdxk	pyridoxal (pyridoxine, vitamin B6) kinase	Chr10: 77.899506	10.42145614	15.8	Chr1: 11.505582	−0.577729592	49	7.82628×10^{-6}	0.463	−0.193162393	0.34283969
1427492_at	69693	11785	Pof1b	premature ovarian failure 1B	ChrX: 109.752131	10.20796491	14.1	Chr12: 101.866283	0.577678571	49	7.84531×10^{-6}	0.446	0.426324786	0.030896857
1450714_at	53814	9410	Oazin	ornithine decarboxylase antizyme 3	Chr15: 38.418247	11.51212281	12.1	Chr1: 187.001839	0.57755102	49	7.89306×10^{-6}	0.4	0	1
1434678_at	102927	–	AI661274	expressed sequence AI661274	ChrX: 48.466709	9.714701754	15.2	Chr11: 28.095395	0.575612245	49	8.65259×10^{-6}	0	0	1
1422869_at	17289	4626	Mertk	c-mer proto-oncogene tyrosine kinase	Chr2: 128.627834	10.16119298	80	Chr2: 125.304784	−0.574234694	49	9.23247×10^{-6}	0.506	−0.073504274	0.720500632
1427894_at	246154	36929	Slitl2	Slit-like 2 (Drosophila)	Chr16: 4.650276	9.791596491	11	Chr2: 75.856729	−0.574158163	49	9.2657×10^{-6}	0.461	0	1
1429722_at	77757	–	9230111I22Rik	RIKEN cDNA 9230111I22 gene	Chr11: 69.594158	9.73645614	16.1	Chr7: 136.179208	−0.573903061	49	9.3773×10^{-6}	0	0	1
1425815_a_at	15366	8271	Hmmr	hyaluronan mediated motility receptor (RHAMM)	Chr11: 40.514973	7.142385965	12.5	Chr12: 85.872820	0.573826531	49	9.41102×10^{-6}	0.526	−0.126837607	0.535373594
1437250_at	59002	9857	Wdt2	WD repeat domain 8	Chr1: 72.205833	8.935157895	16.7	Chr12: 101.866283	0.573367347	49	9.61567×10^{-6}	0.322	0	1

Table S4. Top 100 genes most correlated to *Col3a1* (probe 1427884) from Mouse data of HZI Lung M430v2 (Apr08) RMA Database.

Record ID	Gene ID	Homologene ID	Symbol	Description	Location (Chr: Mb)	Mean Expr	Max LRS	Max LRS Location (Chr: Mb)	Sample Rho	*N* Cases	Sample P(rho)	Lit Corr	Tissue Rho	Tissue P(rho)
1427884_at	12825	55433	Col3a1	procollagen, type III, alpha 1 (Ehlers-Danlos syndrome types IV, aortic and arterial aneurysms)	Chr1: 45.405965	10.13378947	13.5	Chr12: 101.866283	1	49	0	1	1	8.8842×10^{-8}
1427883_a_at	12825	55433	Col3a1	procollagen, type 3, alpha 1 (Ehlers-Danlos syndrome types IV, aortic and arterial aneurysms)	Chr1: 45.404600	13.32673684	11.7	Chr12: 101.866283	0.926020408	49	0	1	1	8.8842×10^{-8}
1450625_at	12832	20119	Col5a2	procollagen, type V, alpha 2	Chr1: 45.431498	9.820280702	27.3	Chr1: 43.500859	0.883265306	49	0	0.87	0.870769231	1.8367×10^{-6}
1460208_at	14118	30958	Fbn1	fibrillin 1 (Marfan syndrome)	Chr2: 125.126430	11.54631579	12.2	Chr16: 45.072728	0.850943878	49	0	0.61	0.884444444	1.7195×10^{-6}
1439827_at	239337	12808	Adamts 12	a disintegrin-like and metalloprotease with thrombospondin type 1 motif, 12	Chr15: 11.278497	8.218105263	15	Chr9: 80.917762	0.773443878	49	3.0493×10^{-12}	0.472	0.773675214	6.8425×10^{-6}
1423110_at	12843	69	Col1a2	procollagen, type 1, alpha 2	Chr6: 4.490998	11.05824561	10.2	Chr11: 114.532621	0.771505102	49	3.8443×10^{-12}	0.877	0.867350427	1.8568×10^{-6}
1434413_at	16000	515	Igf1	insulin-like growth factor 1 (somatomedin C)	Chr10: 87.399205	9.974526316	11.9	Chr6: 124.006511	0.768341837	49	5.5773×10^{-12}	0.418	0.305299145	0.12935937
1422437_at	12832	20119	Col5a2	procollagen, type V, alpha 2	Chr1: 45.432766	11.33133333	12.8	Chr6: 9.485705	0.764566327	49	8.616×10^{-12}	0.87	0.870769231	1.8367×10^{-6}
1425896_a_at	14118	30958	Fbn1	fibrillin 1	Chr2: 125.127175	10.48815789	10.8	Chr11: 20.853303	0.740408163	49	1.1226×10^{-10}	0.61	0.884444444	1.7195×10^{-6}
1455494_at	12842	73874	Col1a1	procollagen, type I, alpha 1 (osteogenesis imperfecta types I-IV, Ehlers-Danlos syndrome type VIIA, Ehlers-Danlos syndrome classical type, Caffey Disease, idiopathic osteoporosis)	Chr11: 94.814020	10.11817544	10.4	Chr18: 68.674127	0.73744898	49	1.502×10^{-10}	0.81	0.717606838	5.8279×10^{-5}
1450691_at	140721	32485	Caskin2	cask-interacting protein 2	Chr11: 115.660538	10.76840351	12.5	Chr12: 101.866283	−0.73380102	49	2.1368×10^{-10}	0.147	0.309401709	0.12409401
1452264_at	209039	37077	Tenc1	tensin like C1 domain-containing phosphatase	Chr15: 101.946167	11.68761404	12.7	Chr11: 11.016916	−0.732015306	49	2.5329×10^{-10}	0.57	0.379145299	0.05696087
1418187_at	54409	4274	Ramp2	receptor (calcitonin) activity modifying protein 2	Chr11: 101.108917	13.05791228	15.5	Chr12: 101.866283	−0.729872449	49	3.0997×10^{-10}	0.333	0.595213675	0.00164414

Table S4. *Cont.*

Record ID	Gene ID	Homologene ID	Symbol	Description	Location (Chr: Mb)	Mean Expr	Max LRS	Max LRS Location (Chr: Mb)	Sample Rho	*N* Cases	Sample P(rho)	Lit Corr	Tissue Rho	Tissue P(rho)
1452424_at	78134	3871	Lpar4	lysophosphatidic acid receptor 4	ChrX: 104.126532	6.065596491	23.3	Chr11: 4.408731	0.723647959	49	5.5014×10^{-10}	0.373	0.745641026	2.083×10^{-5}
1451516_at	69159	5477	Rhebl1	Ras homolog enriched in brain like 1	Chr15: 98.708467	8.949789474	12.2	Chr9: 90.299275	−0.719693878	49	7.8449×10^{-10}	0.227	−0.052991453	0.7969061
1451978_at	16949	4074	Loxl1	lysyl oxidase-like 1	Chr9: 58.136428	11.50540351	12.7	Chr16: 45.072728	0.716785714	49	1.0138×10^{-9}	0.646	0.727863248	4.0563×10^{-5}
1422921_at	22364	7598	Vpreb3	pre-B lymphocyte gene 3	Chr10: 75.411885	9.317859649	10.4	Chr5: 23.471050	−0.702959184	49	3.2623×10^{-9}	0.336	−0.122051282	0.55101485
1448429_at	27357	31219	Gyg1	glycogenin 1	Chr3: 20.022210	11.8312807	9.3	Chr19: 10.157494	0.700612245	49	3.9471×10^{-9}	0.569	0	1
1419513_a_at	13605	7298	Ect2	ect2 oncogene	Chr3: 26.996196	6.402894737	17.4	Chr12: 101.866283	0.699897959	49	4.1809×10^{-9}	0.341	−0.262222222	0.19493591
1455239_at	320802	–	6330512 M04Rik	RIKEN cDNA 6330512M04 gene	Chr7: 149.541434	8.935631579	13.4	Chr11: 26.370844	−0.699380086	49	4.3585×10^{-9}	0	−0.140536846	0.49349063
1423608_at	16431	31269	Itm2a	integral membrane protein 2A	ChrX: 104.592554	10.73319298	16.3	Chr5: 113.832344	0.699311224	49	4.3827×10^{-9}	0.498	0.402393162	0.04252824
1437401_at	16000	515	Igf1	insulin-like growth factor 1 (somatomedin C)	Chr10: 87.396551	9.840754386	13.2	Chr6: 117.050638	0.698239796	49	4.7749×10^{-9}	0.418	0.305299145	0.12935937
1418300_a_at	17347	49674	Mknk2	MAP kinase-interacting serine/threonine kinase 2	Chr10: 80.128102	12.37989474	12.7	Chr11: 8.736727	−0.698137755	49	4.8139×10^{-9}	0.343	0.416752137	0.03519184
1451596_a_at	20698	39748	Sphk1	sphingosine kinase 1	Chr11: 116.397453	10.09082456	18.9	Chr12: 101.866283	−0.696913265	49	5.3062×10^{-9}	0.438	0.323760684	0.10691427
1437410_at	11669	55480	Aldh2	aldehyde dehydrogenase 2, mitochondrial	Chr5: 122.021169	10.14747368	15.5	Chr1: 11.505582	−0.695688776	49	5.8454×10^{-9}	0.388	0.05982906	0.77119164
1416221_at	14314	5144	Fstl1	follistatin-like 1	Chr16: 37.835326	11.57580702	15.6	Chr6: 114.213342	0.694056122	49	6.6447×10^{-9}	0.563	0.793504274	3.3806×10^{-6}
1427894_at	246154	36929	Slitl2	Slit-like 2 (Drosophila)	Chr16: 4.650276	9.791596491	11	Chr2: 75.856729	−0.692729592	49	7.3686×10^{-9}	0.461	0	1
1429139_at	229603	10624	Otud7b	OTU domain containing 7B	Chr3: 95.964567	9.959859649	11.6	Chr16: 60.328963	−0.687015306	49	1.1418×10^{-8}	0.295	0.312820513	0.11982912
1440964_s_at	12398	74543	Cbfa2t3	core-binding factor, runt domain, alpha subunit 2, translocated to, 3 homolog (transcriptional repressor, TAL-1 complex)	Chr8: 125.149089	9.149192982	11.1	Chr11: 11.016916	−0.682755102	49	1.5708×10^{-8}	0.325	0.104273504	0.6109123
1419519_at	16000	515	Igf1	insulin-like growth factor 1 (somatomedin C)	Chr10: 87.393985	8.584631579	10.9	Chr5: 119.758226	0.682678571	49	1.5797×10^{-8}	0.418	0.305299145	0.12935937
1428548_at	70584	4300	Pak4	p21 (CDKN1A)-activated kinase 4	Chr7: 29.343958	10.5287193	14.6	Chr15: 73.370668	−0.681811224	49	1.6401×10^{-8}	0.388	0.128205128	0.53094421
1436481_at	11979	20063	Atp7b	ATPase, Cu++ transporting, beta polypeptide	Chr8: 23.103479	9.749	12.7	Chr2: 109.829995	−0.679846939	49	1.8942×10^{-8}	0.484	−0.455042735	0.0204997
1427929_a_at	216134	2731	Pdxk	pyridoxal (pyridoxine, vitamin B6) kinase	Chr10: 77.899506	10.42145614	15.8	Chr1: 11.505582	−0.679413265	49	1.9551×10^{-8}	0.463	−0.193162393	0.34283969
1449151_at	18557	1949	Pctk3	PCTAIRE-motif protein kinase 3	Chr1: 134.010383	9.397754386	14.7	Chr11: 8.736727	−0.67755102	49	2.2379×10^{-8}	0.352	−0.169230769	0.40683049

Table S4. *Cont.*

Record ID	Gene ID	Homologene ID	Symbol	Description	Location (Chr: Mb)	Mean Expr	Max LRS	Max LRS Location (Chr: Mb)	Sample Rho	*N* Cases	Sample P(rho)	Lit Corr	Tissue Rho	Tissue P(rho)
1424408_at	225341	41214	Lims2	LIM and senescent cell antigen like domains 2	Chr18: 32.117769	11.37566667	11.3	Chr9: 47.039894	−0.676403061	49	2.4309×10^{-8}	0.399	0.43042735	0.02919176
1416242_at	67455	23571	Klhl13	kelch-like 13 (Drosophila)	ChrX: 22.796609	7.874403509	9.9	Chr1: 11.505582	0.67619898	49	2.4668×10^{-8}	0	0.48034188	0.01391366
1435928_at	234797	8825	Kiaa0513	KIAA0513 protein (neuroplasticity associated)	Chr8: 122.688606	9.620035088	14.3	Chr11: 24.572748	−0.674362245	49	2.8129×10^{-8}	0.296	0	1
1449283_a_at	29857	55705	Mapk12	mitogen-activated protein kinase 12	Chr15: 88.961205	10.60645614	15.1	Chr11: 35.501290	−0.674234694	49	2.8385×10^{-8}	0.365	0.511794872	0.00827895
1437584_at	69544	41307	Wdr5b	WD repeat domain 5B	Chr17: 47.656287	7.817824561	10.4	Chr19: 10.157494	−0.671045918	49	3.5555×10^{-8}	0.297	0.019487179	0.92547308
1427492_at	69693	11785	Pof1b	premature ovarian failure 1B	ChrX: 109.752131	10.20796491	14.1	Chr12: 101.866283	0.669617347	49	3.9289×10^{-8}	0.446	0.426324786	0.03089686
1459205_at	12877	7278	Cpeb1	cytoplasmic polyadenylation element binding protein 1	Chr7: 88.577468	7.637350877	16.7	Chr1: 3.482275	−0.669566327	49	3.9428×10^{-8}	0.39	0.344957265	0.08491207
1429722_at	77757	–	9230111I22Rik	RIKEN cDNA 9230111I22 gene	Chr11: 69.594158	9.73645614	16.1	Chr7: 136.179208	−0.669362245	49	3.9993×10^{-8}	0	0	1
1452832_s_at	110911	37854	Cds2	CDP-diacylglycerol synthase (phosphatidate cytidylyltransferase) 2	Chr2: 132.137365	11.48801754	17.2	Chr11: 11.016916	−0.669336735	49	4.0064×10^{-8}	0.359	−0.230769231	0.25552815
1424175_at	21685	31140	Tef	thyrotroph embryonic factor	Chr15: 81.656697	11.77547368	17.5	Chr1: 5.008089	−0.668903061	49	4.129×10^{-8}	0.45	0.145982906	0.47502523
1448306_at	18035	7863	Nfkbia	nuclear factor of kappa light chain gene enhancer in B-cells inhibitor, alpha	Chr12: 56.591432	11.94278947	15.2	Chr5: 76.574921	−0.665790816	49	5.1172×10^{-8}	0.403	0.315555556	0.11649659

Table S4. *Cont.*

Record ID	Gene ID	Homologene ID	Symbol	Description	Location (Chr: Mb)	Mean Expr	Max LRS	Max LRS Location (Chr: Mb)	Sample Rho	*N* Cases	Sample P(rho)	Lit Corr	Tissue Rho	Tissue P(rho)
1443933_at	74413	12560	Mtac2d1	membrane targeting (tandem) C2 domain containing 1	Chr12: 102.883731	9.013087719	62.5	Chr12: 101.866283	0.663086735	49	6.1511×10^{-8}	0.093	0.108376068	0.59684737
1448181_at	66277	8553	Klf15	Kruppel-like factor 15	Chr6: 90.424644	10.59536842	11.1	Chr6: 9.485705	−0.662959184	49	6.2044×10^{-8}	0.436	0.076239316	0.71049389
1435727_s_at	65970	9484	D15Ertd366e	DNA segment, Chr 15, ERATO Doi 366, expressed	Chr15: 99.608972	10.71405263	14.5	Chr1: 11.505582	−0.661760204	49	6.7268×10^{-8}	0.391	0	1
1427509_at	214505	13047	Gnptg	*N*-acetylglucosamine-1-phosphotransferase, gamma subunit	Chr17: 25.379638	8.564210526	12.2	Chr12: 101.866283	−0.66127551	49	6.9494×10^{-8}	0.459	−0.467350427	0.01703148
1417169_at	53376	3098	Usp2	ubiquitin specific protease 2	Chr9: 43.903240	9.733982456	13.3	Chr1: 11.505582	−0.658852041	49	8.1694×10^{-8}	0.396	−0.067350427	0.74318122
1415800_at	14609	136	Gja1	gap junction membrane channel protein alpha 1	Chr10: 56.109688	11.15501754	16.5	Chr11: 8.736727	0.656760204	49	9.3803×10^{-8}	0.403	0.439316239	0.02575988
1434479_at	12831	55434	Col5a1	procollagen, type V, alpha 1	Chr2: 27.894230	9.543754386	14.9	Chr18: 69.067889	0.656607143	49	9.4751×10^{-8}	0.892	0.790769231	3.68×10^{-6}
1418192_at	17428	7842	Mnt	max binding protein	Chr11: 74.658710	10.36491228	16.3	Chr11: 8.736727	−0.655459184	49	1.0216×10^{-7}	0.386	−0.234871795	0.24698917
1419329_at	20410	4218	Sh3d4	SH3 domain protein 4	Chr14: 70.580340	11.10742105	10.7	Chr11: 8.736727	−0.653290816	49	1.1764×10^{-7}	0.405	0	1
1448162_at	22329	838	Vcam1	vascular cell adhesion molecule 1	Chr3: 115.813116	10.75026316	10.6	Chr1: 3.482275	0.653086735	49	1.1921×10^{-7}	0.451	−0.271111111	0.17980585
1416808_at	18073	1878	Nid1	nidogen 1	Chr13: 13.603982	11.54373684	12.8	Chr6: 126.754346	0.651938776	49	1.2837×10^{-7}	0.623	0.838632479	1.9238×10^{-6}
1422587_at	56277	41215	Tmem45a	transmembrane protein 45a	Chr16: 56.806532	9.58777193	22.9	Chr16: 56.463019	0.64994898	49	1.4584×10^{-7}	0.273	0.660854701	0.0003321
1449731_s_at	18035	7863	Nfkbia	nuclear factor of kappa light chain gene enhancer in B-cells inhibitor, alpha	Chr12: 56.590517	11.94147368	13.4	Chr12: 87.446646	−0.64880102	49	1.569×10^{-7}	0.403	0.315555556	0.11649659
1416741_at	12831	55434	Col5a1	procollagen, type V, alpha 1	Chr2: 27.893658	8.612017544	11	Chr16: 51.051259	0.648290816	49	1.6207×10^{-7}	0.892	0.790769231	3.68×10^{-6}
1449851_at	18626	1966	Per1	period 1	Chr11: 68.922874	10.46289474	17.1	Chr1: 5.008089	−0.645663265	49	1.9125×10^{-7}	0.317	0.245811966	0.22515742
1440156_s_at	269389	13155	LOC269389	embryonic retinal HMG-box protein	Chr2: 163.149678	11.17026316	14.3	Chr11: 8.736727	−0.644387755	49	2.0712×10^{-7}	0.344	0	1
1455665_at	–	–	Lonrf1	LON peptidase N-terminal domain and ring finger 1	Chr8: 37.279195	10.07385965	18.6	Chr1: 5.008089	−0.643418367	49	2.1999×10^{-7}	0	−0.009230769	0.96536045
1448816_at	19223	37374	Ptgis	prostaglandin I2 (prostacyclin) synthase (coagulation, essential hypertension, also known as CYP8A1)	Chr2: 167.028802	11.87012281	12.6	Chr11: 35.501290	−0.643392857	49	2.2034×10^{-7}	0.552	0.458461538	0.0194825
1451069_at	223775	56955	Pim3	proviral integration site 3	Chr15: 88.695562	10.40852632	14.5	Chr9: 68.191194	−0.641760204	49	2.4375×10^{-7}	0.377	0.234188034	0.24839894

Table S4. *Cont.*

Record ID	Gene ID	Homologene ID	Symbol	Description	Location (Chr: Mb)	Mean Expr	Max LRS	Max LRS Location (Chr: Mb)	Sample Rho	*N* Cases	Sample P(rho)	Lit Corr	Tissue Rho	Tissue P(rho)
1417638_at	13590	49231	Leftb	left right determination factor 1 (endometrial bleeding associated factor)	Chr1: 182.867972	8.624175439	17	Chr1: 187.001839	−0.640586735	49	2.6198×10^{-7}	0.334	0	1
1448269_a_at	67455	23571	Klhl13	kelch-like 13	ChrX: 22.796459	9.212614035	11.4	Chr1: 5.008089	0.640408163	49	2.6486×10^{-7}	0	0.48034188	0.01391366
1427541_x_at	15366	8271	Hmmr	hyaluronan mediated motility receptor (RHAMM)	Chr11: 92.536885	7.050631579	17.3	Chr11: 19.645819	0.639515306	49	2.7972×10^{-7}	0.526	−0.126837607	0.53537359
1432184_a_at	70435	82406	Inf2	inverted formin 2 (formin inverted 2)	Chr12: 113.853235	8.642333333	12.5	Chr9: 105.676711	−0.639311224	49	2.8323×10^{-7}	0.201	0.29025641	0.15008058
1437992_x_at	14609	136	Gja1	gap junction membrane channel protein alpha 1	Chr10: 56.110123	11.78289474	11.4	Chr6: 131.940470	0.638979592	49	2.8901×10^{-7}	0.403	0.439316239	0.02575988
1455137_at	217944	56563	Rapgef5	Rap guanine nucleotide exchange factor (GEF) 5	Chr12: 118.997620	11.97096491	15.3	Chr16: 60.328963	−0.638112245	49	3.0466×10^{-7}	0.239	−0.178803419	0.38046075
1438157_s_at	18035	7863	Nfkbia	nuclear factor of kappa light chain gene enhancer in B-cells inhibitor, alpha	Chr12: 56.590866	12.43996491	13.1	Chr11: 4.408731	−0.637576531	49	3.1471×10^{-7}	0.403	0.315555556	0.11649659
1459522_s_at	27357	31219	Gyg1	glycogenin 1	Chr3: 20.022094	12.54947368	13.3	Chr8: 18.512781	0.636964286	49	3.2658×10^{-7}	0.569	0	1
1417168_a_at	53376	3098	Usp2	ubiquitin specific protease 2	Chr9: 43.900386	8.421894737	16.2	Chr1: 11.505582	−0.636556122	49	3.3472×10^{-7}	0.396	−0.067350427	0.74318122
1434140_at	17207	11804	Mcf2l	mcf.2 transforming sequence-like	Chr8: 13.020122	10.71173684	11.2	Chr11: 24.572748	−0.636020408	49	3.4569×10^{-7}	0.333	−0.063931624	0.75587544
1456331_at	319792		9130023H24Rik	RIKEN cDNA 9130023H24 gene	Chr7: 135.378234	8.665438596	10.8	Chr9: 95.050077	0.634413265	49	3.8065×10^{-7}	0	0	1
1424126_at	11655	55478	Alas1	aminolevulinic acid synthase 1	Chr9: 106.136456	12.35468421	12.5	Chr1: 5.008089	−0.634311224	49	3.8297×10^{-7}	0.475	0.145982906	0.47502523
1424914_at	217732	14124	2310044G17Rik	RIKEN cDNA 2310044G17 gene	Chr12: 88.305731	9.246350877	14.6	Chr11: 8.736727	−0.634260204	49	3.8414×10^{-7}	0	−0.147350427	0.47085555
1456315_a_at	30963	69153	Ptpla	protein tyrosine phosphatase-like (proline instead of catalytic arginine), member A (3-hydroxyacyl-CoA dehydrates 1)	Chr2: 162.724687	10.33866667	15.8	Chr7: 36.124856	0.632219388	49	4.3368×10^{-7}	0.442	0.528888889	0.00612672
1449439_at	93691	2751	Klf7	Kruppel-like factor 7 (ubiquitous)	Chr1: 64.079453	10.55264912	12.3	Chr9: 79.991491	−0.63127551	49	4.5855×10^{-7}	0.384	0.186324786	0.36046256
1434895_s_at	21981	9090	Ppp1r13b	protein phosphatase 1, regulatory (inhibitor) subunit 13B	Chr12: 113.066695	11.11117544	12.5	Chr2: 121.412768	−0.628715018	49	5.3283×10^{-7}	0.289	−0.265641026	0.18901491
1427879_at	69171	9238	1810031K17Rik	RIKEN cDNA 1810031K17 gene	Chr1: 75.132378	10.84263158	15.3	Chr11: 4.408731	−0.628545918	49	5.3811×10^{-7}	0	0.291623932	0.14810306

Table S4. *Cont.*

Record ID	Gene ID	Homologene ID	Symbol	Description	Location (Chr: Mb)	Mean Expr	Max LRS	Max LRS Location (Chr: Mb)	Sample Rho	*N* Cases	Sample P(rho)	Lit Corr	Tissue Rho	Tissue P(rho)
1437150_at	242297	17518	1700012 H17Rik	RIKEN cDNA 1700012H17	Chr4: 5.726501	8.107719298	12.2	Chr11: 19.879675	0.62815378	49	5.5054×10^{-7}	0.152	0.131623932	0.51994893
1435261_at	387314	65299	Tmtc1	transmembrane and tetratricopeptide repeat	Chr6: 148.181012	10.77201754	12.8	Chr9: 68.739814	−0.627729592	49	5.6428×10^{-7}	0	0.394188034	0.04724219
1419703_at	53867	9253	Col5a3	procollagen, type V, alpha 3	Chr9: 20.574515	8.048280702	11.8	Chr9: 116.482254	0.626709184	49	5.9866×10^{-7}	0.761	0.606153846	0.00128943
1452242_at	74107	10019	Cep55	centrosomal protein 55	Chr19: 38.148423	7.502210526	10.8	Chr12: 100.818236	0.626428571	49	6.0845×10^{-7}	0.315	0.052991453	0.7969061
1455220_at	212398	8095	Frat2	frequently rearranged in advanced T-cell lymphomas 2	Chr19: 41.920580	9.031561404	9.1	Chr9: 68.191194	−0.626020408	49	6.2296×10^{-7}	0.306	−0.111794872	0.58523522
1421346_a_at	21366	2291	Slc6a6	solute carrier family 6 (neurotransmitter transporter, taurine), member 6	Chr6: 91.702282	9.739122807	10.5	ChrX: 163.741514	−0.625994898	49	6.2388×10^{-7}	0.502	-0.08034188	0.69557309
1450757_at	12552	1361	Cdh11	cadherin 11	Chr8: 105.157629	11.29538596	11.8	Chr19: 10.249020	0.62505102	49	6.5871×10^{-7}	0.54	0.128888889	0.52873619
1449491_at	105844	8728	Card10	caspase recruitment domain family, member 10	Chr15: 78.605591	10.92507018	13.4	Chr9: 81.753446	−0.624566327	49	6.7729×10^{-7}	0.307	0.277264957	0.16983035
1447818_x_at	69159	5477	Rhebl1	Ras homolog enriched in brain like 1	Chr15: 98.708276	8.328859649	9.6	Chr2: 130.881867	−0.621326531	49	8.1451×10^{-7}	0.227	-0.052991453	0.7969061
1455493_at	64009	52329	Syne1	spectrin repeat containing, nuclear envelope 1 (synaptic nuclear envelope 1, cerebellar ataxia)	Chr10: 5.325868	10.68415789	11.3	Chr11: 11.016916	−0.619923469	49	8.8159×10^{-7}	0.371	0.140512821	0.49189535
1420854_at	13717	73880	Eln	elastin	Chr5: 135.179594	9.945719298	13.9	Chr9: 83.587153	0.619413265	49	9.0723×10^{-7}	0.685	0.763418803	1.0294×10^{-5}
1435435_at	30785	14125	Cttnbp2	cortactin binding protein 2	Chr6: 18.316732	6.90745614	8.7	Chr12: 104.269891	0.619158163	49	9.203×10^{-7}	0.405	0.00034188	1
1418582_at	12398	74543	Cbfa2t3	core-binding factor, runt domain, alpha subunit 2, translocated to, 3 homolog (transcriptional repressor, TAL-1 complex)	Chr8: 125.153919	9.498157895	14.4	Chr9: 103.827402	−0.619107143	49	9.2294×10^{-7}	0.325	0.104273504	0.6109123
1449259_at	19340	20902	Rab3d	RAB3D, member RAS oncogene family	Chr9: 21.712002	9.801561404	14.8	Chr1: 20.774306	−0.618596939	49	9.4969×10^{-7}	0.378	0.487863248	0.01233732
1429739_a_at	56218	8636	Patz1	POZ (BTB) and AT hook containing zinc finger 1	Chr11: 3.208489	9.876122807	8.9	Chr2: 121.412768	−0.618265306	49	9.6746×10^{-7}	0.329	-0.045470085	0.82543596
1437250_at	59002	9857	Wdt2	WD repeat domain 8	Chr1: 72.205833	8.935157895	16.7	Chr12: 101.866283	0.618061224	49	9.7854×10^{-7}	0.322	0	1
1448276_at	64540	2453	Tspan4	tetraspanin 4	Chr7: 148.677933	11.06194737	11.7	Chr1: 5.008089	−0.617857143	49	9.8975×10^{-7}	0.275	0.315951449	0.11585864

Table S4. *Cont.*

Record ID	Gene ID	Homologene ID	Symbol	Description	Location (Chr: Mb)	Mean Expr	Max LRS	Max LRS Location (Chr: Mb)	Sample Rho	*N* Cases	Sample P(rho)	Lit Corr	Tissue Rho	Tissue P(rho)
1425364_a_at	17254	1795	Slc3a2	solute carrier family 3 (activators of dibasic and neutral amino acid transport), member 2 (cysteine-glutamate exchanger component)	Chr19: 8.782197	12.13592982	9.6	Chr11: 16.853389	−0.617091837	49	1.0328×10^{-6}.	0.535	−0.045470085	0.82543596
1452837_at	64898	8769	Lpin2	lipin 2	Chr17: 71.597161	10.30714035	10.8	Chr9: 68.191194	−0.615306122	49	1.1402×10^{-6}.	0.435	−0.525901871	0.00579064

Table S5. Top 100 gene associated with *COL3A1* in human based on microarray using microarray probe ID 100149328.

Record	Gene ID	Homologene ID	Symbol	Description	Location (Chr: Mb)	Mean Expr
100149328_TGI_at	1281	55433	COL3A1	collagen, type III, alpha 1	Chr2: 189.839099	12.23052601
100310834_TGI_at	1281	55433	COL3A1	collagen, type III, alpha 1	Chr2: 189.839099	11.44610894
100303661_TGI_at	1281	55433	COL3A1	collagen, type III, alpha 1	Chr2: 189.839099	11.98108212
100312351_TGI_at	1278	69	COL1A2	collagen, type I, alpha 2	Chr7: 94.023873	11.64942439
100305860_TGI_at	1278	69	COL1A2	collagen, type I, alpha 2	Chr7: 94.023873	11.6883683
100303662_TGI_at	1278	69	COL1A2	collagen, type I, alpha 2	Chr7: 94.023873	11.82141544
100304433_TGI_at	1277	73874	COL1A1	collagen, type I, alpha 1	Chr17: 48.261457	11.98945448
100147092_TGI_at	1277	73874	COL1A1	collagen, type I, alpha 1	Chr17: 48.261457	12.27460325
100122545_TGI_at	1277	73874	COL1A1	collagen, type I, alpha 1	Chr17: 48.261457	9.140987809
100307955_TGI_at	–	–	Affy_100307955_TGI_at	Affymetrix HuRSTA probeset 100307955_TGI_at	ChrUn: 1.000000	8.738569121
100130851_TGI_at	1282	20437	COL4A1	collagen, type IV, alpha 1	Chr13: 110.801310	11.10786341
100304693_TGI_at	1289	55434	COL5A1	collagen, type V, alpha 1	Chr9: 137.533652	10.95065772
100303660_TGI_at	1289	55434	COL5A1	collagen, type V, alpha 1	Chr9: 137.533652	10.76253413
100144650_TGI_at	–	–	Affy_100144650_TGI_at	Affymetrix HuRSTA probeset 100144650_TGI_at	ChrUn: 1.000000	10.74903658
100126306_TGI_at	1293	37917	COL6A3	collagen, type VI, alpha 3	Chr2: 238.232655	12.17726179

Table S5. *Cont.*

Record	Gene ID	Homologene ID	Symbol	Description	Location (Chr: Mb)	Mean Expr
100312654_TGI_at	–	–	Affy_100312654_TGI_at	Affymetrix HuRSTA probeset 100312654_TGI_at	ChrUn: 1.000000	8.06398375
100302533_TGI_at	7070	4580	THY1	Thy-1 cell surface antigen	Chr11: 119.288655	10.19936992
100149915_TGI_at	1289	55434	COL5A1	collagen, type V, alpha 1	Chr9: 137.533652	9.349114643
100159693_TGI_at	1290	20119	COL5A2	collagen, type V, alpha 2	Chr2: 189.896641	9.198343097
100155966_TGI_at	7070	4580	THY1	Thy-1 cell surface antigen	Chr11: 119.288655	9.019458535
100313409_TGI_at	1289	55434	COL5A1	collagen, type V, alpha 1	Chr9: 137.533652	10.68843822
100311405_TGI_at	1278	69	COL1A2	collagen, type I, alpha 2	Chr7: 94.023873	12.60051219
100305505_TGI_at	1293	37917	COL6A3	collagen, type VI, alpha 3	Chr2: 238.232655	11.5714813
100139140_TGI_at	56265	10485	CPXM1	carboxypeptidase X (M14 family), member 1	Chr20: 2.774715	8.148152031
100303626_TGI_at	1290	20119	COL5A2	collagen, type V, alpha 2	Chr2: 189.896641	8.757986166
100157343_TGI_at	2200	30958	FBN1	fibrillin 1	Chr15: 48.700503	11.68255854
100309000_TGI_at	7058	2438	THBS2	thrombospondin 2	Chr6: 169.615875	10.03661707
100146287_TGI_at	115908	16320	CTHRC1	collagen triple helix repeat containing 1	Chr8: 104.383743	8.714510562
100146474_TGI_at	1278	69	COL1A2	collagen, type I, alpha 2	Chr7: 94.023873	12.84999513
100307617_TGI_at	2200	30958	FBN1	fibrillin 1	Chr15: 48.700503	10.23058374
100146924_TGI_at	7058	2438	THBS2	thrombospondin 2	Chr6: 169.615875	11.73337887
100303753_TGI_at	3479	515	IGF1	insulin-like growth factor 1 (somatomedin C)	Chr12: 102.789645	9.312289423
100305420_TGI_at	25878	56704	MXRA5	matrix-remodelling associated 5	ChrX: 3.226606	9.870952857
100153006_TGI_at	60681	7718	FKBP10	FK506 binding protein 10, 65 kDa	Chr17: 39.968962	9.944827644
100124337_TGI_at	51148	22954	CERCAM	cerebral endothelial cell adhesion molecule	Chr9: 131.182759	8.971634156
100142387_TGI_at	5738	7908	PTGFRN	prostaglandin F2 receptor inhibitor	Chr1: 117.452689	10.36817479
100158154_TGI_at	25878	56704	MXRA5	matrix-remodelling associated 5	ChrX: 3.226606	10.9882569
100132178_TGI_at	2326	55520	FMO1	flavin containing monooxygenase 1	Chr1: 171.217663	7.74260569
100131659_TGI_at	1306	1396	COL15A1	collagen, type XV, alpha 1	Chr9: 101.706138	10.4193439
100151531_TGI_at	5118	1946	PCOLCE	procollagen C-endopeptidase enhancer	Chr7: 100.199882	10.64065529
100146430_TGI_at	26002	22904	MOXD1	monooxygenase, DBH-like 1	Chr6: 132.617194	8.68016342

Table S5. *Cont.*

Record	Gene ID	Homologene ID	Symbol	Description	Location (Chr: Mb)	Mean Expr
100159580_TGI_at	57722	10570	IGDCC4	immunoglobulin superfamily, DCC subclass, member 4	Chr15: 65.673825	9.116917871
100129738_TGI_at	3479	515	IGF1	insulin-like growth factor 1 (somatomedin C)	Chr12: 102.789645	9.711926825
100122306_TGI_at	1293	37917	COL6A3	collagen, type VI, alpha 3	Chr2: 238.232655	7.666637405
100313396_TGI_at	151887	12206	CCDC80	coiled-coil domain containing 80	Chr3: 112.323407	10.95636017
100158925_TGI_at	11167	5144	FSTL1	follistatin-like 1	Chr3: 120.113061	12.61614717
100133960_TGI_at	283208	27943	P4HA3	prolyl 4-hydroxylase, alpha polypeptide III	Chr11: 73.977702	6.84583659
100312710_TGI_at	–	–	Affy_100312710_TGI_at	Affymetrix HuRSTA probeset 100312710_TGI_at	ChrUn: 1.000000	12.12667317
100146158_TGI_at	3479	515	IGF1	insulin-like growth factor 1 (somatomedin C)	Chr12: 102.789645	9.12427723
100141956_TGI_at	871	20331	SERPINH1	serpin peptidase inhibitor, clade H (heat shock protein 47), member 1, (collagen binding protein 1)	Chr11: 75.273101	10.32392277
100146562_TGI_at	25903	18546	OLFML2B	olfactomedin-like 2B	Chr1: 161.952982	10.26613983
100133163_TGI_at	4237	1801	MFAP2	microfibrillar-associated protein 2	Chr1: 17.300997	8.585326015
100128692_TGI_at	10381	68503	TUBB3	tubulin, beta 3 class III	Chr16: 89.988417	7.596529269
100137132_TGI_at	7058	2438	THBS2	thrombospondin 2	Chr6: 169.615875	6.706712197
100133589_TGI_at	2200	30958	FBN1	fibrillin 1	Chr15: 48.700503	9.372678868
100124472_TGI_at	84624	19648	FNDC1	fibronectin type III domain containing 1	Chr6: 159.590429	9.183451219
100129535_TGI_at	57124	10699	CD248	CD248 molecule, endosialin	Chr11: 66.081958	9.290394307
100311041_TGI_at	4313	3329	MMP2	matrix metallopeptidase 2 (gelatinase A, 72 kDa gelatinase, 72 kDa type IV collagenase)	Chr16: 55.513081	11.61267236
100141687_TGI_at	9201	74530	DCLK1	doublecortin-like kinase 1	Chr13: 36.342789	9.443137389
100302549_TGI_at	10381	68503	TUBB3	tubulin, beta 3 class III	Chr16: 89.988417	8.094108125
100310333_TGI_at	55959	10313	SULF2	sulfatase 2	Chr20: 46.286150	10.24235202
100311885_TGI_at	151887	12206	CCDC80	coiled-coil domain containing 80	Chr3: 112.323407	11.71957724
100162201_TGI_at	84627	18937	ZNF469	zinc finger protein 469	Chr16: 88.493879	9.23859187

Table S5. *Cont.*

Record	Gene ID	Homologene ID	Symbol	Description	Location (Chr: Mb)	Mean Expr
100307370_TGI_at	1462	3228	VCAN	versican	Chr5: 82.767493	11.71631137
100160133_TGI_at	151887	12206	CCDC80	coiled-coil domain containing 80	Chr3: 112.323407	11.72754309
100152906_TGI_at	8532	2709	CPZ	carboxypeptidase Z	Chr4: 8.594387	8.683573173
100313254_TGI_at	493869	88454	GPX8	glutathione peroxidase 8 (putative)	Chr5: 54.455984	9.449026825
100147093_TGI_at	55816	10195	DOK5	docking protein 5	Chr20: 53.092266	7.30501138
100134891_TGI_at	10631	4730	POSTN	periostin, osteoblast specific factor	Chr13: 38.136719	11.42594389
100134442_TGI_at	–	–	Affy_100134442_TGI_at	Affymetrix HuRSTA probeset 100134442_TGI_at	ChrUn: 1.000000	8.06451626
100308789_TGI_at	26002	22904	MOXD1	monooxygenase, DBH-like 1	Chr6: 132.617194	11.08348699
100138137_TGI_at	1462	3228	VCAN	versican	Chr5: 82.767493	10.94685203
100303044_TGI_at	813	936	CALU	calumenin	Chr7: 128.379346	11.43390569
100155101_TGI_at	6447	37722	SCG5	secretogranin V (7B2 protein)	Chr15: 32.933870	7.987365041
100125027_TGI_at	8038	74862	ADAM12	ADAM metallopeptidase domain 12	Chr10: 127.702898	8.253901633
100143513_TGI_at	5480	727	PPIC	peptidylprolyl isomerase C (cyclophilin C)	Chr5: 122.359078	11.3834244
100126529_TGI_at	1734	621	DIO2	deiodinase, iodothyronine, type II	Chr14: 80.663868	8.752215431
100139809_TGI_at	5176	1965	SERPINF1	serpin peptidase inhibitor, clade F (alpha-2 antiplasmin, pigment epithelium derived factor), member 1	Chr17: 1.665259	12.19294715
100313780_TGI_at	10631	4730	POSTN	periostin, osteoblast specific factor	Chr13: 38.136719	10.94822927
100310073_TGI_at	55816	10195	DOK5	docking protein 5	Chr20: 53.092266	7.519772362
100121726_TGI_at	2199	1514	FBLN2	fibulin 2	Chr3: 13.590625	10.24280812
100129601_TGI_at	9509	8597	ADAMTS2	ADAM metallopeptidase with thrombospondin type 1 motif, 2	Chr5: 178.537852	8.829549595
100158155_TGI_at	1278	69	COL1A2	collagen, type I, alpha 2	Chr7: 94.023873	12.61587723
100156990_TGI_at	7373	18741	COL14A1	collagen, type XIV, alpha 1	Chr8: 121.137352	10.48131707
100306389_TGI_at	6447	37722	SCG5	secretogranin V (7B2 protein)	Chr15: 32.933870	7.821728454
100142596_TGI_at	493869	88454	GPX8	glutathione peroxidase 8 (putative)	Chr5: 54.455984	10.18585854

Table S5. *Cont.*

Record	Gene ID	Homologene ID	Symbol	Description	Location (Chr: Mb)	Mean Expr
100156990_TGI_at	7373	18741	COL14A1	collagen, type XIV, alpha 1	Chr8: 121.137352	10.48131707
100306389_TGI_at	6447	37722	SCG5	secretogranin V (7B2 protein)	Chr15: 32.933870	7.821728454
100142596_TGI_at	493869	88454	GPX8	glutathione peroxidase 8 (putative)	Chr5: 54.455984	10.18585854
100159918_TGI_at	4920	55831	ROR2	receptor tyrosine kinase-like orphan receptor 2	Chr9: 94.484878	8.951102446
100145673_TGI_at	1291	1391	COL6A1	collagen, type VI, alpha 1	Chr21: 47.401663	11.68139025
100312692_TGI_at	2200	30958	FBN1	fibrillin 1	Chr15: 48.700503	7.596117888
100156623_TGI_at	1282	20437	COL4A1	collagen, type IV, alpha 1	Chr13: 110.801310	9.935031695
100145950_TGI_at	7076	36321	TIMP1	TIMP metallopeptidase inhibitor 1	ChrX: 47.441690	12.08160895
100151955_TGI_at	220323	14334	OAF	OAF homolog (Drosophila)	Chr11: 120.081747	9.135436596
100123900_TGI_at	4313	3329	MMP2	matrix metallopeptidase 2 (gelatinase A, 72 kDa gelatinase, 72 kDa type IV collagenase)	Chr16: 55.513081	10.36621788
100134517_TGI_at	4016	4074	LOXL1	lysyl oxidase-like 1	Chr15: 74.218789	8.855014624
100300787_TGI_at	151887	12206	CCDC80	coiled-coil domain containing 80	Chr3: 112.323407	9.472299995
100146341_TGI_at	55959	10313	SULF2	sulfatase 2	Chr20: 46.286150	10.30839025
100313225_TGI_at	51661	22568	FKBP7	FK506 binding protein 7	Chr2: 179.328391	9.450363413
100134262_TGI_at	5738	7908	PTGFRN	prostaglandin F2 receptor inhibitor	Chr1: 117.452689	8.738223571
100303776_TGI_at	1282	20437	COL4A1	collagen, type IV, alpha 1	Chr13: 110.801310	9.443007321
100310393_TGI_at	23213	49408	SULF1	sulfatase 1	Chr8: 70.378859	10.82945204

Table S6. Top 100 gene associated with *COL3A1* in human based on microarray using microarray probe ID 100303661.

Record ID	Gene ID	Homologene ID	Symbol	Description	Location (Chr: Mb)	Mean Expr	Sample Rho	*N* Cases	Sample P(rho)	Lit Corr	Tissue Rho	Tissue P(rho)
100303661_TGI_at	1281	55433	COL3A1	collagen, type III, alpha 1	Chr2: 189.839099	11.98108212	1	1230	0	0	1	8.88415×10^{-8}
100149328_TGI_at	1281	55433	COL3A1	collagen, type III, alpha 1	Chr2: 189.839099	12.23052601	0.950717492	1230	0	0	1	8.88415×10^{-8}
100303662_TGI_at	1278	69	COL1A2	collagen, type I, alpha 2	Chr7: 94.023873	11.82141544	0.940340121	1230	0	0	0.867350427	1.85681×10^{-6}
100312351_TGI_at	1278	69	COL1A2	collagen, type I, alpha 2	Chr7: 94.023873	11.64942439	0.939900002	1230	0	0	0.867350427	1.85681×10^{-6}
100305860_TGI_at	1278	69	COL1A2	collagen, type I, alpha 2	Chr7: 94.023873	11.6883683	0.937478533	1230	0	0	0.867350427	1.85681×10^{-6}
100310834_TGI_at	1281	55433	COL3A1	collagen, type III, alpha 1	Chr2: 189.839099	11.44610894	0.934755038	1230	0	0	1	8.88415×10^{-8}
100304433_TGI_at	1277	73874	COL1A1	collagen, type I, alpha 1	Chr17: 48.261457	11.98945448	0.876182402	1230	0	0	0.717606838	5.82788×10^{-5}
100147092_TGI_at	1277	73874	COL1A1	collagen, type I, alpha 1	Chr17: 48.261457	12.27460325	0.864324334	1230	0	0	0.717606838	5.82788×10^{-5}
100122545_TGI_at	1277	73874	COL1A1	collagen, type I, alpha 1	Chr17: 48.261457	9.140987809	0.840655009	1230	0	0	0.717606838	5.82788×10^{-5}
100307955_TGI_at	–	–	Affy_100307955_TGI_at	Affymetrix HuRSTA probeset 100307955_TGI_at	ChrUn: 1.000000	8.738569121	0.82111831	1230	0	0	0	1
100126306_TGI_at	1293	37917	COL6A3	collagen, type VI, alpha 3	Chr2: 238.232655	12.17726179	0.806747991	1230	0	0	0.828376068	1.96459×10^{-6}
100130851_TGI_at	1282	20437	COL4A1	collagen, type IV, alpha 1	Chr13: 110.801310	11.10786341	0.793477937	1230	0	0	0.6	0.001479817
100304693_TGI_at	1289	55434	COL5A1	collagen, type V, alpha 1	Chr9: 137.533652	10.95065772	0.780537271	1230	0	0	0.790769231	3.67995×10^{-6}
100303660_TGI_at	1289	55434	COL5A1	collagen, type V, alpha 1	Chr9: 137.533652	10.76253413	0.772610127	1230	0	0	0.790769231	3.67995×10^{-6}
100144650_TGI_at	–	–	Affy_100144650_TGI_at	Affymetrix HuRSTA probeset 100144650_TGI_at	ChrUn: 1.000000	10.74903658	0.766315601	1230	0	0	0	1
100311405_TGI_at	1278	69	COL1A2	collagen, type I, alpha 2	Chr7: 94.023873	12.60051219	0.761539391	1230	0	0	0.867350427	1.85681×10^{-6}
100313409_TGI_at	1289	55434	COL5A1	collagen, type V, alpha 1	Chr9: 137.533652	10.68843822	0.747738593	1230	0	0	0.790769231	3.67995×10^{-6}
100303626_TGI_at	1290	20119	COL5A2	collagen, type V, alpha 2	Chr2: 189.896641	8.757986166	0.739062103	1230	0	0	0.870769231	1.83671×10^{-6}
100305505_TGI_at	1293	37917	COL6A3	collagen, type VI, alpha 3	Chr2: 238.232655	11.5714813	0.736204554	1230	0	0	0.828376068	1.96459×10^{-6}
100302533_TGI_at	7070	4580	THY1	Thy-1 cell surface antigen	Chr11: 119.288655	10.19936992	0.735133833	1230	0	0	0.288205128	0.153082676
100155966_TGI_at	7070	4580	THY1	Thy-1 cell surface antigen	Chr11: 119.288655	9.019458535	0.720252888	1230	0	0	0.288205128	0.153082676
100312654_TGI_at	–	–	Affy_100312654_TGI_at	Affymetrix HuRSTA probeset 100312654_TGI_at	ChrUn: 1.000000	8.06398375	0.709035165	1230	0	0	0	1
100146474_TGI_at	1278	69	COL1A2	collagen, type I, alpha 2	Chr7: 94.023873	12.84999513	0.70229549	1230	0	0	0.867350427	1.85681×10^{-6}
100149915_TGI_at	1289	55434	COL5A1	collagen, type V, alpha 1	Chr9: 137.533652	9.349114643	0.701283428	1230	0	0	0.790769231	3.67995×10^{-6}

Table S6. *Cont.*

Record ID	Gene ID	Homologene ID	Symbol	Description	Location (Chr: Mb)	Mean Expr	Sample Rho	*N* Cases	Sample P(rho)	Lit Corr	Tissue Rho	Tissue P(rho)
100159693_TGI_at	1290	20119	COL5A2	collagen, type V, alpha 2	Chr2: 189.896641	9.198343097	0.699539492	1230	0	0	0.870769231	1.83671×10^{-6}
100307617_TGI_at	2200	30958	FBN1	fibrillin 1	Chr15: 48.700503	10.23058374	0.686124094	1230	0	0	0.884444444	1.71952×10^{-6}
100139140_TGI_at	56265	10485	CPXM1	carboxypeptidase X (M14 family), member 1	Chr20: 2.774715	8.148152031	0.677237755	1230	0	0	0.34974359	0.080464331
100157343_TGI_at	2200	30958	FBN1	fibrillin 1	Chr15: 48.700503	11.68255854	0.656005948	1230	0	0	0.884444444	1.71952×10^{-6}
100131659_TGI_at	1306	1396	COL15A1	collagen, type XV, alpha 1	Chr9: 101.706138	10.4193439	0.655547957	1230	0	0	0.809230769	2.34024×10^{-6}
100305420_TGI_at	25878	56704	MXRA5	matrix-remodelling associated 5	ChrX: 3.226606	9.870952857	0.649563672	1230	0	0	0	1
100309000_TGI_at	7058	2438	THBS2	thrombospondin 2	Chr6: 169.615875	10.03661707	0.643484546	1230	0	0	0.730598291	3.67246×10^{-5}
100158154_TGI_at	25878	56704	MXRA5	matrix-remodelling associated 5	ChrX: 3.226606	10.9882569	0.640241391	1230	0	0	0	1
100158155_TGI_at	1278	69	COL1A2	collagen, type I, alpha 2	Chr7: 94.023873	12.61587723	0.637733919	1230	0	0	0.867350427	1.85681×10^{-6}
100146287_TGI_at	–	16320	CTHRC1	collagen triple helix repeat containing 1	Chr8: 104.383743	8.714510562	0.634395314	1230	0	0	0.220892463	0.27818493
100146924_TGI_at	7058	2438	THBS2	thrombospondin 2	Chr6: 169.615875	11.73337887	0.63170264	1230	0	0	0.730598291	3.67246×10^{-5}
100308789_TGI_at	26002	22904	MOXD1	monooxygenase, DBH-like 1	Chr6: 132.617194	11.08348699	0.628521975	1230	0	0	0.456410256	0.020087681
100158925_TGI_at	11167	5144	FSTL1	follistatin-like 1	Chr3: 120.113061	12.61614717	0.610115346	1230	0	0	0.793504274	3.38062×10^{-6}
100124472_TGI_at	84624	19648	FNDC1	fibronectin type III domain containing 1	Chr6: 159.590429	9.183451219	0.599635802	1230	0	0	0.535042735	0.005478277
100142387_TGI_at	5738	7908	PTGFRN	prostaglandin F2 receptor inhibitor	Chr1: 117.452689	10.36817479	0.597482388	1230	0	0	0.418875027	0.033182973
100303753_TGI_at	3479	515	IGF1	insulin-like growth factor 1 (somatomedin C)	Chr12: 102.789645	9.312289423	0.591167199	1230	0	0	0.305299145	0.129359367
100147093_TGI_at	55816	10195	DOK5	docking protein 5	Chr20: 53.092266	7.30501138	0.587990893	1230	0	0	−0.109420415	0.594657396
100143513_TGI_at	5480	727	PPIC	peptidylprolyl isomerase C (cyclophilin C)	Chr5: 122.359078	11.3834244	0.585576298	1230	0	0	0.693675214	0.000127713
100313780_TGI_at	10631	4730	POSTN	periostin, osteoblast specific factor	Chr13: 38.136719	10.94822927	0.583325116	1230	0	0	0.556239316	0.003670959

Table S6. *Cont.*

Record ID	Gene ID	Homologene ID	Symbol	Description	Location (Chr: Mb)	Mean Expr	Sample Rho	*N* Cases	Sample P(rho)	Lit Corr	Tissue Rho	Tissue P(rho)
100134891_TGI_at	10631	4730	POSTN	periostin, osteoblast specific factor	Chr13: 38.136719	11.42594389	0.581321071	1230	0	0	0.556239316	0.003670959
100313254_TGI_at	–	88454	GPX8	glutathione peroxidase 8 (putative)	Chr5: 54.455984	9.449026825	0.579642023	1230	0	0	0	1
100312710_TGI_at	–	–	Affy_100312710_TGI_at	Affymetrix HuRSTA probeset 100312710_TGI_at	ChrUn: 1.000000	12.12667317	0.577637429	1230	0	0	0	1
100310073_TGI_at	55816	10195	DOK5	docking protein 5	Chr20: 53.092266	7.519772362	0.576312429	1230	0	0	−0.109420415	0.594657396
100311041_TGI_at	4313	3329	MMP2	matrix metallopeptidase 2 (gelatinase A, 72 kDa gelatinase, 72 kDa type IV collagenase)	Chr16: 55.513081	11.61267236	0.576227907	1230	0	0	0.833162393	1.93902×10^{-6}
100129738_TGI_at	3479	515	IGF1	insulin-like growth factor 1 (somatomedin C)	Chr12: 102.789645	9.711926825	0.570716125	1230	0	0	0.305299145	0.129359367
100151531_TGI_at	5118	1946	PCOLCE	procollagen C-endopeptidase enhancer	Chr7: 100.199882	10.64065529	0.570421492	1230	0	0	0.514529915	0.007896969
100146430_TGI_at	26002	22904	MOXD1	monooxygenase, DBH-like 1	Chr6: 132.617194	8.68016342	0.567939379	1230	0	0	0.456410256	0.020087681
100133163_TGI_at	4237	1801	MFAP2	microfibrillar-associated protein 2	Chr1: 17.300997	8.585326015	0.567517641	1230	0	0	0.654700855	0.00039206
100132178_TGI_at	2326	55520	FMO1	flavin containing monooxygenase 1	Chr1: 171.217663	7.74260569	0.5670369	1230	0	0	0.23965812	0.237270266
100313396_TGI_at	151887	12206	CCDC80	coiled-coil domain containing 80	Chr3: 112.323407	10.95636017	0.566110683	1230	0	0	0.832478632	1.94181×10^{-6}
100148227_TGI_at	813	936	CALU	calumenin	Chr7: 128.379346	12.02766342	0.565487351	1230	0	0	0.762051282	1.08743×10^{-5}
100302549_TGI_at	10381	68503	TUBB3	tubulin, beta 3 class III	Chr16: 89.988417	8.094108125	0.564637439	1230	0	0	−0.348376068	0.081716339
100146158_TGI_at	3479	515	IGF1	insulin-like growth factor 1 (somatomedin C)	Chr12: 102.789645	9.12427723	0.563632801	1230	0	0	0.305299145	0.129359367
100153006_TGI_at	60681	7718	FKBP10	FK506 binding protein 10, 65 kDa	Chr17: 39.968962	9.944827644	0.563343779	1230	0	0	0.688205128	0.000151113
100159580_TGI_at	57722	10570	IGDCC4	immunoglobulin superfamily, DCC subclass, member 4	Chr15: 65.673825	9.116917871	0.561631241	1230	0	0	0	1
100303044_TGI_at	813	936	CALU	calumenin	Chr7: 128.379346	11.43390569	0.559650168	1230	0	0	0.762051282	1.08743×10^{-5}
100141687_TGI_at	9201	74530	DCLK1	doublecortin-like kinase 1	Chr13: 36.342789	9.443137389	0.552695297	1230	0	0	−0.016068376	0.93875377
100133589_TGI_at	2200	30958	FBN1	fibrillin 1	Chr15: 48.700503	9.372678868	0.551037714	1230	0	0	0.884444444	1.71952×10^{-6}
100126529_TGI_at	1734	621	DIO2	deiodinase, iodothyronine, type II	Chr14: 80.663868	8.752215431	0.545183801	1230	0	0	0.063931624	0.755875442
100139809_TGI_at	5176	1965	SERPINF1	serpin peptidase inhibitor, clade F (alpha-2 antiplasmin, pigment epithelium derived factor), member 1	Chr17: 1.665259	12.19294715	0.544134736	1230	0	0	0.497435897	0.01054977
100311885_TGI_at	151887	12206	CCDC80	coiled-coil domain containing 80	Chr3: 112.323407	11.71957724	0.543474076	1230	0	0	0.832478632	1.94181×10^{-6}

Table S6. *Cont.*

Record ID	Gene ID	Homologene ID	Symbol	Description	Location (Chr: Mb)	Mean Expr	Sample Rho	*N* Cases	Sample P(rho)	Lit Corr	Tissue Rho	Tissue P(rho)
100158069_TGI_at	4320	38116	MMP11	matrix metallopeptidase 11 (stromelysin 3)	Chr22: 24.115036	7.463882932	0.542701228	1230	0	0	0.626666667	0.000798478
100307370_TGI_at	1462	3228	VCAN	versican	Chr5: 82.767493	11.71631137	0.54057693	1230	0	0	0.882393162	1.74077×10^{-6}
100128692_TGI_at	10381	68503	TUBB3	tubulin, beta 3 class III	Chr16: 89.988417	7.596529269	0.540017449	1230	0	0	−0.348376068	0.081716339
100124337_TGI_at	51148	22954	CERCAM	cerebral endothelial cell adhesion molecule	Chr9: 131.182759	8.971634156	0.537968957	1230	0	0	0	1
100121726_TGI_at	2199	1514	FBLN2	fibulin 2	Chr3: 13.590625	10.24280812	0.532448284	1230	0	0	0.878974359	1.77337×10^{-6}
100123900_TGI_at	4313	3329	MMP2	matrix metallopeptidase 2 (gelatinase A, 72kDa gelatinase, 72kDa type IV collagenase)	Chr16: 55.513081	10.36621788	0.532404722	1230	0	0	0.833162393	1.93902×10^{-6}
100160133_TGI_at	151887	12206	CCDC80	coiled-coil domain containing 80	Chr3: 112.323407	11.72754309	0.532313446	1230	0	0	0.832478632	1.94181×10^{-6}
100134707_TGI_at	1462	3228	VCAN	versican	Chr5: 82.767493	11.41867968	0.530812274	1230	0	0	0.882393162	1.74077×10^{-6}
100126539_TGI_at	716	1314	C1S	complement component 1, s subcomponent	Chr12: 7.167980	11.96761625	0.530691921	1230	0	0	0.391452991	0.048903035
100306389_TGI_at	6447	37722	SCG5	secretogranin V (7B2 protein)	Chr15: 32.933870	7.821728454	0.529791234	1230	0	0	−0.323076923	0.10768934
100155101_TGI_at	6447	37722	SCG5	secretogranin V (7B2 protein)	Chr15: 32.933870	7.987365041	0.529218179	1230	0	0	−0.323076923	0.10768934
100142596_TGI_at	–	88454	GPX8	glutathione peroxidase 8 (putative)	Chr5: 54.455984	10.18585854	0.528041166	1230	0	0	0	1
100156990_TGI_at	7373	18741	COL14A1	collagen, type XIV, alpha 1	Chr8: 121.137352	10.48131707	0.527329774	1230	0	0	0.524786325	0.006594228
100300662_TGI_at	1282	20437	COL4A1	collagen, type IV, alpha 1	Chr13: 110.801310	10.8331504	0.526275291	1230	0	0	0.6	0.001479817
100150236_TGI_at	–	–	Affy_100150236_TGI_at	Affymetrix HuRSTA probeset 100150236_TGI_at	ChrUn: 1.000000	8.558981307	0.52580627	1230	0	0	0	1
100138137_TGI_at	1462	3228	VCAN	versican	Chr5: 82.767493	10.94685203	0.524864138	1230	0	0	0.882393162	1.74077×10^{-6}
100146562_TGI_at	25903	18546	OLFML2B	olfactomedin-like 2B	Chr1: 161.952982	10.26613983	0.520006288	1230	0	0	0.794871795	3.24691×10^{-6}
100300787_TGI_at	151887	12206	CCDC80	coiled-coil domain containing 80	Chr3: 112.323407	9.472299995	0.517469559	1230	0	0	0.832478632	1.94181×10^{-6}
100148535_TGI_at	57669	32492	EPB41L5	erythrocyte membrane protein band 4.1 like 5	Chr2: 120.770604	10.06848294	-0.516827438	1230	0	0	0	1
100129535_TGI_at	57124	10699	CD248	CD248 molecule, endosialin	Chr11: 66.081958	9.290394307	0.516017201	1230	0	0	0.885128205	1.71216×10^{-6}
100300285_TGI_at	–	–	Affy_100300285_TGI_at	Affymetrix HuRSTA probeset 100300285_TGI_at	ChrUn: 1.000000	11.18176341	0.514541961	1230	0	0	0	1
100127785_TGI_at	7291	402	TWIST1	twist basic helix-loop-helix transcription factor 1	Chr7: 19.155091	9.024633339	0.511640761	1230	0	0	0.742905983	2.31486×10^{-5}

Table S6. *Cont.*

Record ID	Gene ID	Homologene ID	Symbol	Description	Location (Chr: Mb)	Mean Expr	Sample Rho	*N* Cases	Sample P(rho)	Lit Corr	Tissue Rho	Tissue P(rho)
100143579_TGI_at	493869	88454	GPX8	glutathione peroxidase 8 (putative)	Chr5: 54.455984	8.604664222	0.508859554	1230	0	0	0	1
100312692_TGI_at	2200	30958	FBN1	fibrillin 1	Chr15: 48.700503	7.596117888	0.50837423	1230	0	0	0.884444444	1.71952×10^{-6}
100155982_TGI_at	23176	57205	SEPT8	septin 8	Chr5: 132.086509	9.488988624	0.507285911	1230	0	0	0.638290598	0.000599585
100152906_TGI_at	8532	2709	CPZ	carboxypeptidase Z	Chr4: 8.594387	8.683573173	0.507072974	1230	0	0	0.526837607	0.006356832
100310333_TGI_at	55959	10313	SULF2	sulfatase 2	Chr20: 46.286150	10.24235202	0.50593648	1230	0	0	0.608205128	0.001230833
100305648_TGI_at	–	–	Affy_100305648_TGI_at	Affymetrix HuRSTA probeset 100305648_TGI_at	ChrUn: 1.000000	10.50597805	0.50512366	1230	0	0	0	1
100303669_TGI_at	154	30948	ADRB2	adrenoceptor beta 2, surface	Chr5: 148.206156	10.06234066	−0.504026586	1230	0	0	0.368205128	0.064972362
100155088_TGI_at	154	30948	ADRB2	adrenoceptor beta 2, surface	Chr5: 148.206156	11.44784227	−0.503918964	1230	0	0	0.368205128	0.064972362
100145673_TGI_at	1291	1391	COL6A1	collagen, type VI, alpha 1	Chr21: 47.401663	11.68139025	0.503659269	1230	0	0	0.773675214	6.84245×10^{-6}
100312280_TGI_at	6566	20662	SLC16A1	solute carrier family 16, member 1 (monocarboxylic acid transporter 1)	Chr1: 113.454469	11.09682847	0.502573101	1230	0	0	−0.228034188	0.261328093
100308196_TGI_at	3075	20086	CFH	complement factor H	Chr1: 196.621008	6.472499188	0.502157675	1230	0	0	0.096068376	0.639452776
100305296_TGI_at	6447	37722	SCG5	secretogranin V (7B2 protein)	Chr15: 32.933870	7.305438208	0.500388454	1230	0	0	−0.323076923	0.10768934
100122535_TGI_at	3221	8408	HOXC4	homeobox C4	Chr12: 54.410642	8.189264219	0.496994323	1230	0	0	−0.220512821	0.277721919

Table S7. Top 100 gene associated with *COL3A1* in human based on microarray using Microarray probe ID 100310834_TGI.

Record ID	Gene ID	Homologene ID	Symbol	Description	Location (Chr: Mb)	Mean Expr	Sample Rho	*N* Cases	Sample P(rho)	Lit Corr	Tissue Rho	Tissue P(rho)
100310834_TGI_at	1281	55433	COL3A1	collagen, type III, alpha 1	Chr2: 189.839099	11.44610894	1	1230	0	0	1	8.88415×10^{-8}
100149328_TGI_at	1281	55433	COL3A1	collagen, type III, alpha 1	Chr2: 189.839099	12.23052601	0.965721505	1230	0	0	1	8.88415×10^{-8}
100303661_TGI_at	1281	55433	COL3A1	collagen, type III, alpha 1	Chr2: 189.839099	11.98108212	0.934755038	1230	0	0	1	8.88415×10^{-8}
100303662_TGI_at	1278	69	COL1A2	collagen, type I, alpha 2	Chr7: 94.023873	11.82141544	0.916504179	1230	0	0	0.867350427	1.85681×10^{-6}
100312351_TGI_at	1278	69	COL1A2	collagen, type I, alpha 2	Chr7: 94.023873	11.64942439	0.888562417	1230	0	0	0.867350427	1.85681×10^{-6}
100305860_TGI_at	1278	69	COL1A2	collagen, type I, alpha 2	Chr7: 94.023873	11.6883683	0.881657202	1230	0	0	0.867350427	1.85681×10^{-6}
100304433_TGI_at	1277	73874	COL1A1	collagen, type I, alpha 1	Chr17: 48.261457	11.98945448	0.843020314	1230	0	0	0.717606838	5.82788×10^{-5}
100122545_TGI_at	1277	73874	COL1A1	collagen, type I, alpha 1	Chr17: 48.261457	9.140987809	0.835265946	1230	0	0	0.717606838	5.82788×10^{-5}
100147092_TGI_at	1277	73874	COL1A1	collagen, type I, alpha 1	Chr17: 48.261457	12.27460325	0.828115294	1230	0	0	0.717606838	5.82788×10^{-5}
100130851_TGI_at	1282	20437	COL4A1	collagen, type IV, alpha 1	Chr13: 110.801310	11.10786341	0.820017257	1230	0	0	0.6	0.001479817
100307955_TGI_at	–	–	Affy_100307955_TGI_at	Affymetrix HuRSTA probeset 100307955_TGI_at	ChrUn: 1.000000	8.738569121	0.810800491	1230	0	0	0	1
100126306_TGI_at	1293	37917	COL6A3	collagen, type VI, alpha 3	Chr2: 238.232655	12.17726179	0.757252517	1230	0	0	0.828376068	1.96459×10^{-6}
100159693_TGI_at	1290	20119	COL5A2	collagen, type V, alpha 2	Chr2: 189.896641	9.198343097	0.74965089	1230	0	0	0.870769231	1.83671×10^{-6}
100303626_TGI_at	1290	20119	COL5A2	collagen, type V, alpha 2	Chr2: 189.896641	8.757986166	0.742196856	1230	0	0	0.870769231	1.83671×10^{-6}
100157343_TGI_at	2200	30958	FBN1	fibrillin 1	Chr15: 48.700503	11.68255854	0.738880656	1230	0	0	0.884444444	1.71952×10^{-6}
100304693_TGI_at	1289	55434	COL5A1	collagen, type V, alpha 1	Chr9: 137.533652	10.95065772	0.737976636	1230	0	0	0.790769231	3.67995×10^{-6}
100312654_TGI_at	–	–	Affy_100312654_TGI_at	Affymetrix HuRSTA probeset 100312654_TGI_at	ChrUn: 1.000000	8.06398375	0.728047088	1230	0	0	0	1
100146287_TGI_at	115908	16320	CTHRC1	collagen triple helix repeat containing 1	Chr8: 104.383743	8.714510562	0.723970162	1230	0	0	0.220892463	0.27818493
100303660_TGI_at	1289	55434	COL5A1	collagen, type V, alpha 1	Chr9: 137.533652	10.76253413	0.721551151	1230	0	0	0.790769231	3.67995×10^{-6}
100144650_TGI_at	–	–	Affy_100144650_TGI_at	Affymetrix HuRSTA probeset 100144650_TGI_at	ChrUn: 1.000000	10.74903658	0.715469205	1230	0	0	0	1
100302533_TGI_at	7070	4580	THY1	Thy-1 cell surface antigen	Chr11: 119.288655	10.19936992	0.709637792	1230	0	0	0.288205128	0.153082676
100155966_TGI_at	7070	4580	THY1	Thy-1 cell surface antigen	Chr11: 119.288655	9.019458535	0.707799082	1230	0	0	0.288205128	0.153082676
100309000_TGI_at	7058	2438	THBS2	thrombospondin 2	Chr6: 169.615875	10.03661707	0.704421242	1230	0	0	0.730598291	3.67246×10^{-5}
100313409_TGI_at	1289	55434	COL5A1	collagen, type V, alpha 1	Chr9: 137.533652	10.68843822	0.703224395	1230	0	0	0.790769231	3.67995×10^{-6}

Table S7. *Cont.*

Record ID	Gene ID	Homologene ID	Symbol	Description	Location (Chr: Mb)	Mean Expr	Sample Rho	*N* Cases	Sample P(rho)	Lit Corr	Tissue Rho	Tissue P(rho)
100146924_TGI_at	7058	2438	THBS2	thrombospondin 2	Chr6: 169.615875	11.73337887	0.69803536	1230	0	0	0.730598291	3.67246×10^{-6}
100307617_TGI_at	2200	30958	FBN1	fibrillin 1	Chr15: 48.700503	10.23058374	0.698031483	1230	0	0	0.884444444	1.71952×10^{-6}
100149915_TGI_at	1289	55434	COL5A1	collagen, type V, alpha 1	Chr9: 137.533652	9.349114643	0.687593838	1230	0	0	0.790769231	3.67995×10^{-6}
100139140_TGI_at	56265	10485	CPXM1	carboxypeptidase X (M14 family), member 1	Chr20: 2.774715	8.148152031	0.67453842	1230	0	0	0.34974359	0.080464331
100131659_TGI_at	1306	1396	COL15A1	collagen, type XV, alpha 1	Chr9: 101.706138	10.4193439	0.667933276	1230	0	0	0.809230769	2.34024×10^{-6}
100311405_TGI_at	1278	69	COL1A2	collagen, type I, alpha 2	Chr7: 94.023873	12.60051219	0.656128279	1230	0	0	0.867350427	1.85681×10^{-6}
100305505_TGI_at	1293	37917	COL6A3	collagen, type VI, alpha 3	Chr2: 238.232655	11.5714813	0.649493716	1230	0	0	0.828376068	1.96459×10^{-6}
100142387_TGI_at	5738	7908	PTGFRN	prostaglandin F2 receptor inhibitor	Chr1: 117.452689	10.36817479	0.637194921	1230	0	0	0.418875027	0.033182973
100303753_TGI_at	3479	515	IGF1	insulin-like growth factor 1 (somatomedin C)	Chr12: 102.789645	9.312289423	0.632189917	1230	0	0	0.305299145	0.129359367
100133589_TGI_at	2200	30958	FBN1	fibrillin 1	Chr15: 48.700503	9.372678868	0.631016193	1230	0	0	0.884444444	1.71952×10^{-6}
100146158_TGI_at	3479	515	IGF1	insulin-like growth factor 1 (somatomedin C)	Chr12: 102.789645	9.12427723	0.629820282	1230	0	0	0.305299145	0.129359367
100159580_TGI_at	57722	10570	IGDCC4	immunoglobulin superfamily, DCC subclass, member 4	Chr15: 65.673825	9.116917871	0.629570955	1230	0	0	0	1
100141687_TGI_at	9201	74530	DCLK1	doublecortin-like kinase 1	Chr13: 36.342789	9.443137389	0.625439401	1230	0	0	-0.016068376	0.93875377
100132178_TGI_at	2326	55520	FMO1	flavin containing monooxygenase 1	Chr1: 171.217663	7.74260569	0.625040931	1230	0	0	0.23965812	0.237270266
100129738_TGI_at	3479	515	IGF1	insulin-like growth factor 1 (somatomedin C)	Chr12: 102.789645	9.711926825	0.620376961	1230	0	0	0.305299145	0.129359367
100143513_TGI_at	5480	727	PPIC	peptidylprolyl isomerase C (cyclophilin C)	Chr5: 122.359078	11.3834244	0.617880188	1230	0	0	0.693675214	0.000127713
100156990_TGI_at	7373	18741	COL14A1	collagen, type XIV, alpha 1	Chr8: 121.137352	10.48131707	0.613587189	1230	0	0	0.524786325	0.006594228
100146562_TGI_at	25903	18546	OLFML2B	olfactomedin-like 2B	Chr1: 161.952982	10.26613983	0.613469332	1230	0	0	0.794871795	3.24691×10^{-6}
100313396_TGI_at	151887	12206	CCDC80	coiled-coil domain containing 80	Chr3: 112.323407	10.95636017	0.610815102	1230	0	0	0.832478632	1.94181×10^{-6}
100153006_TGI_at	60681	7718	FKBP10	FK506 binding protein 10, 65 kDa	Chr17: 39.968962	9.944827644	0.604393506	1230	0	0	0.688205128	0.000151113
100312692_TGI_at	2200	30958	FBN1	fibrillin 1	Chr15: 48.700503	7.596117888	0.600962908	1230	0	0	0.884444444	1.71952×10^{-6}
100134891_TGI_at	10631	4730	POSTN	periostin, osteoblast specific factor	Chr13: 38.136719	11.42594389	0.59695848	1230	0	0	0.556239316	0.003670959
100142596_TGI_at	493869	88454	GPX8	glutathione peroxidase 8 (putative)	Chr5: 54.455984	10.18585854	0.596869984	1230	0	0	0	1
100307370_TGI_at	1462	3228	VCAN	versican	Chr5: 82.767493	11.71631137	0.593947868	1230	0	0	0.882393162	1.74077×10^{-6}
100305420_TGI_at	25878	56704	MXRA5	matrix-remodelling associated 5	ChrX: 3.226606	9.870952857	0.592242893	1230	0	0	0	1
100126529_TGI_at	1734	621	DIO2	deiodinase, iodothyronine, type II	Chr14: 80.663868	8.752215431	0.591728949	1230	0	0	0.063931624	0.755875442
100313780_TGI_at	10631	4730	POSTN	periostin, osteoblast specific factor	Chr13: 38.136719	10.94822927	0.591719627	1230	0	0	0.556239316	0.003670959
100137132_TGI_at	7058	2438	THBS2	thrombospondin 2	Chr6: 169.615875	6.706712197	0.587881903	1230	0	0	0.730598291	3.67246×10^{-5}

Table S7. *Cont.*

Record ID	Gene ID	Homologene ID	Symbol	Description	Location (Chr: Mb)	Mean Expr	Sample Rho	*N* Cases	Sample P(rho)	Lit Corr	Tissue Rho	Tissue P(rho)
100124472_TGI_at	84624	19648	FNDC1	fibronectin type III domain containing 1	Chr6: 159.590429	9.183451219	0.585111858	1230	0	0	0.535042735	0.005478277
100133960_TGI_at	283208	27943	P4HA3	prolyl 4-hydroxylase, alpha polypeptide III	Chr11: 73.977702	6.84583659	0.583396344	1230	0	0	0.099487179	0.627495875
100134707_TGI_at	1462	3228	VCAN	versican	Chr5: 82.767493	11.41867968	0.58026522	1230	0	0	0.882393162	1.74077×10^{-6}
100302549_TGI_at	10381	68503	TUBB3	tubulin, beta 3 class III	Chr16: 89.988417	8.094108125	0.576955752	1230	0	0	−0.348376068	0.081716339
100147093_TGI_at	55816	10195	DOK5	docking protein 5	Chr20: 53.092266	7.30501138	0.576200744	1230	0	0	−0.109420415	0.594657396
100140394_TGI_at	26872	8256	STEAP1	six transmembrane epithelial antigen of the prostate 1	Chr7: 89.783689	9.195988613	0.575426721	1230	0	0	0.128205128	0.530944212
100300406_TGI_at	–	–	Affy_100300406_TGI_at	Affymetrix HuRSTA probeset 100300406_TGI_at	ChrUn: 1.000000	7.195769106	0.572782231	1230	0	0	0	1
100138137_TGI_at	1462	3228	VCAN	versican	Chr5: 82.767493	10.94685203	0.571628036	1230	0	0	0.882393162	1.74077×10^{-6}
100311921_TGI_at	493869	88454	GPX8	glutathione peroxidase 8 (putative)	Chr5: 54.455984	9.92086829	0.567793558	1230	0	0	0	1
100310393_TGI_at	23213	49408	SULF1	sulfatase 1	Chr8: 70.378859	10.82945204	0.564092143	1230	0	0	0.587008547	0.001962396
100145673_TGI_at	1291	1391	COL6A1	collagen, type VI, alpha 1	Chr21: 47.401663	11.68139025	0.562777998	1230	0	0	0.773675214	6.84245×10^{-6}
100155982_TGI_at	23176	57205	SEPT8	septin 8	Chr5: 132.086509	9.488988624	0.561521053	1230	0	0	0.638290598	0.000599585
100139729_TGI_at	5270	21247	SERPINE2	serpin peptidase inhibitor, clade E (nexin, plasminogen activator inhibitor type 1), member 2	Chr2: 224.839765	10.23939106	0.558145386	1230	0	0	0.288888889	0.152077187
100311885_TGI_at	151887	12206	CCDC80	coiled-coil domain containing 80	Chr3: 112.323407	11.71957724	0.555137287	1230	0	0	0.832478632	1.94181×10^{-6}
100310073_TGI_at	55816	10195	DOK5	docking protein 5	Chr20: 53.092266	7.519772362	0.5551014	1230	0	0	−0.109420415	0.594657396
100147235_TGI_at	2200	30958	FBN1	fibrillin 1	Chr15: 48.700503	8.87112357	0.55414862	1230	0	0	0.884444444	1.71952×10^{-6}
100121726_TGI_at	2199	1514	FBLN2	fibulin 2	Chr3: 13.590625	10.24280812	0.553720267	1230	0	0	0.878974359	1.77337×10^{-6}
100122306_TGI_at	1293	37917	COL6A3	collagen, type VI, alpha 3	Chr2: 238.232655	7.666637405	0.552979956	1230	0	0	0.828376068	1.96459×10^{-6}
100124337_TGI_at	51148	22954	CERCAM	cerebral endothelial cell adhesion molecule	Chr9: 131.182759	8.971634156	0.552234671	1230	0	0	0	1
100305648_TGI_at	–	–	Affy_100305648_TGI_at	Affymetrix HuRSTA probeset 100305648_TGI_at	ChrUn: 1.000000	10.50597805	0.548843668	1230	0	0	0	1
100151083_TGI_at	26585	8022	GREM1	gremlin 1, DAN family BMP antagonist	Chr15: 33.010205	7.15522927	0.548437786	1230	0	0	0.071452991	0.72803588

Table S7. *Cont.*

Record ID	Gene ID	Homologene ID	Symbol	Description	Location (Chr: Mb)	Mean Expr	Sample Rho	*N* Cases	Sample P(rho)	Lit Corr	Tissue Rho	Tissue P(rho)
100300787_TGI_at	151887	12206	CCDC80	coiled-coil domain containing 80	Chr3: 112.323407	9.472299995	0.546620729	1230	0	0	0.832478632	1.94181×10^{-6}
100158154_TGI_at	25878	56704	MXRA5	matrix-remodelling associated 5	ChrX: 3.226606	10.9882569	0.546606372	1230	0	0	0	1
100308789_TGI_at	26002	22904	MOXD1	monooxygenase, DBH-like 1	Chr6: 132.617194	11.08348699	0.546508546	1230	0	0	0.456410256	0.020087681
100160133_TGI_at	151887	12206	CCDC80	coiled-coil domain containing 80	Chr3: 112.323407	11.72754309	0.546093238	1230	0	0	0.832478632	1.94181×10^{-6}
100143692_TGI_at	55203	10048	LGI2	leucine-rich repeat LGI family, member 2	Chr4: 25.000471	7.654302441	0.54572423	1230	0	0	0.303931624	0.131150639
100128692_TGI_at	10381	68503	TUBB3	tubulin, beta 3 class III	Chr16: 89.988417	7.596529269	0.545303688	1230	0	0	−0.348376068	0.081716339
100155088_TGI_at	154	30948	ADRB2	adrenoceptor beta 2, surface	Chr5: 148.206156	11.44784227	-0.54184013	1230	0	0	0.368205128	0.064972362
100129535_TGI_at	57124	10699	CD248	CD248 molecule, endosialin	Chr11: 66.081958	9.290394307	0.541750923	1230	0	0	0.885128205	1.71216×10^{-6}
100308551_TGI_at	1462	3228	VCAN	versican	Chr5: 82.767493	9.506466661	0.540724641	1230	0	0	0.882393162	1.74077×10^{-6}
100148535_TGI_at	57669	32492	EPB41L5	erythrocyte membrane protein band 4.1 like 5	Chr2: 120.770604	10.06848294	−0.538634331	1230	0	0	0	1
100160215_TGI_at	5396	7896	PRRX1	paired related homeobox 1	Chr1: 170.633313	10.7848626	0.537544562	1230	0	0	0.647863248	0.000469407
100312296_TGI_at	5270	21247	SERPINE2	serpin peptidase inhibitor, clade E (nexin, plasminogen activator inhibitor type 1), member 2	Chr2: 224.839765	9.605503241	0.537293953	1230	0	0	0.288888889	0.152077187
100146341_TGI_at	55959	10313	SULF2	sulfatase 2	Chr20: 46.286150	10.30839025	0.53703007	1230	0	0	0.608205128	0.001230833
100155101_TGI_at	6447	37722	SCG5	secretogranin V (7B2 protein)	Chr15: 32.933870	7.987365041	0.537027276	1230	0	0	−0.323076923	0.10768934
100123350_TGI_at	6423	56438	SFRP2	secreted frizzled-related protein 2	Chr4: 154.701742	10.18993983	0.536767751	1230	0	0	0.69982906	0.000105192
100144664_TGI_at	51050	22935	PI15	peptidase inhibitor 15	Chr8: 75.736772	7.788428463	0.535685074	1230	0	0	0.051623932	0.802075272
100146430_TGI_at	26002	22904	MOXD1	monooxygenase, DBH-like 1	Chr6: 132.617194	8.68016342	0.535000166	1230	0	0	0.456410256	0.020087681
100160138_TGI_at	26585	8022	GREM1	gremlin 1, DAN family BMP antagonist	Chr15: 33.010205	6.906791867	0.534366128	1230	0	0	0.071452991	0.72803588
100133163_TGI_at	4237	1801	MFAP2	microfibrillar-associated protein 2	Chr1: 17.300997	8.585326015	0.533090564	1230	0	0	0.654700855	0.00039206
100302370_TGI_at	26585	8022	GREM1	gremlin 1, DAN family BMP antagonist	Chr15: 33.010205	7.52176098	0.532697496	1230	0	0	0.071452991	0.72803588
100310333_TGI_at	55959	10313	SULF2	sulfatase 2	Chr20: 46.286150	10.24235202	0.531803132	1230	0	0	0.608205128	0.001230833
100151937_TGI_at	160428	51942	ALDH1L2	aldehyde dehydrogenase 1 family, member L2	Chr12: 105.413562	7.986523584	0.526527103	1230	0	0	0.466666667	0.017210444
100302479_TGI_at	11015	68533	KDELR3	KDEL (Lys-Asp-Glu-Leu) endoplasmic reticulum protein retention receptor 3	Chr22: 38.864083	8.928736581	0.526187015	1230	0	0	0.67042735	0.00025454

Table S7. *Cont.*

Record ID	Gene ID	Homologene ID	Symbol	Description	Location (Chr: Mb)	Mean Expr	Sample Rho	*N* Cases	Sample P(rho)	Lit Corr	Tissue Rho	Tissue P(rho)
100151531_TGI_at	5118	1946	PCOLCE	procollagen C-endopeptidase enhancer	Chr7: 100.199882	10.64065529	0.525377426	1230	0	0	0.514529915	0.007896969
100146474_TGI_at	1278	69	COL1A2	collagen, type I, alpha 2	Chr7: 94.023873	12.84999513	0.524738433	1230	0	0	0.867350427	1.85681E-06
100313361_TGI_at	2048	37925	EPHB2	EPH receptor B2	Chr1: 23.037331	8.057699184	0.524357414	1230	0	0	0.252649573	0.212200036
100302862_TGI_at	6423	56438	SFRP2	secreted frizzled-related protein 2	Chr4: 154.701742	10.47754716	0.524357161	1230	0	0	0.69982906	0.000105192
